# Mendelian randomization study of interleukin (IL)-1 family and lung cancer

**DOI:** 10.1038/s41598-021-97099-5

**Published:** 2021-09-02

**Authors:** Zhao Yang, C. Mary Schooling, Man Ki Kwok

**Affiliations:** 1 School of Public Health, Li Ka Shing, Faculty of Medicine, The University of Hong Kong, 7 Sassoon Road, Pokfulam, Hong Kong Special Administrative Region China; 2 Graduate School of Public Health and Health Policy, City University of New York, New York, USA

**Keywords:** Cancer epidemiology, Lung cancer

## Abstract

The role of interleukin (IL)-1 family members/receptors in lung cancer remains uncertain due to the susceptibility of observed associations to confounding. We appraised the association of IL-1 family members/receptors with lung cancer and its subtypes [lung adenocarcinoma (LUAD) and squamous cell lung cancer (LUSC)] using two-sample Mendelian randomization. This study found that no IL-1 family members/receptors were significantly associated with lung cancer and its subtypes risk after correction for multiple testing. However, suggestive total effects of increased risk were noted for genetically predicted IL-1Racp with lung cancer (*P* = 0.006), IL-1α with LUAD (*P* = 0.027), and IL-1Racp with LUSC (*P* = 0.008). Suggestive direct effects were also noted for IL-1β, IL-1Ra, IL-36γ with lung cancer, IL-1α/β, IL-1Ra with LUAD, and IL-1β, IL-18BP with LUSC, after adjusting for genetically predicted effects of other IL-1 family members/receptors. Taken together, our findings suggest that interventions decreasing IL-1Racp might protect against lung cancer, perhaps via IL-1α/β or IL-1Ra.

## Introduction

Lung cancer, comprised primarily of lung adenocarcinoma (LUAD) and squamous cell lung cancer (LUSC), is the leading cause of global cancer incidence and mortality with 2.1 million new cases comprising nearly one-fifth of (18.4%) all-cancer deaths in 2018^1^. A recent trial suggested that Canakinumab, a fully human monoclonal antibody targeting interleukin (IL)-1β, reduced lung cancer incidence by 67% and mortality by 77%^2^. However, lung cancer was not a pre-specified trial outcome so this could be a chance finding. To date, definitive evidence about the role of IL-1β in lung cancer is limited, especially in the general population^3–5^. Additionally, observational studies investigating the association of IL-1 inhibitors with lung cancer are open to bias from confounding and selection bias and may also be underpowered^6–9^.

Interleukin-1β, a soluble form of IL-1, is a highly active pro-inflammatory cytokine in nearly all human cells and is thought to play a role in autoinflammatory, autoimmune, infectious, cardiovascular diseases, and cancer along with other IL-1 family members and their receptors/coreceptors (Supplementary Figure S1)^2,10–17^. A previous Mendelian randomization (MR) study, using the randomly allocated genetic variants as instruments to diminish the likelihood to be affected by the commonly encountered confounders (e.g., lifestyle and socioeconomic position)^18^, has shown that long-term dual IL-1α/β inhibition with IL-1Ra may increase risk of coronary heart disease, and conversely, reduce the risk of development of rheumatoid arthritis^14^. Furthermore, a randomized controlled trial showed that Canakinumab lowers IL-1β among patients with a previous myocardial infarction, leading to a reduced risk of experiencing cardiovascular events^10^ and of lung cancer incident and mortality^2^. As well as IL-1β, many other IL-1 family members or receptors (e.g., IL-1α) have been shown to possess similar properties but with diverse effects. For example, MABp1, lowering IL-1α, appears to increase the risk of symptoms in patients with advanced colorectal cancer^9,19^. IL-18 is reported to be a candidate causal marker for lung cancer, as it confers a lower risk of developing lung cancer^20^. Furthermore, dysregulation of IL-36 signaling may have a role in cancer development, including lung, kidneys, and intestines, although few studies were conducted and suggested limited anti-tumor effects^21^.

Nowadays, MR studies are increasingly being used to foreshadow the effects of trials and elucidate causal pathways^18^. Moreover, MR gives clear information about the direction of effect^22^, and thus can provide reliable evidence about the role of IL-1 family members/receptors in lung cancer^2^. To our knowledge, limited evidence from both randomized controlled trials and MR studies investigating the effects of IL-1 family members/receptors on lung cancer and its subtypes exists, especially in the general population.

Several IL-1 family members/receptors are pharmacologically modifiable so that assessing their potential as targets of intervention for lung cancer prevention and investigating their etiological roles in lung cancer are important. To clarify, we conducted an MR study using two genome-wide association studies (GWAS, n = 11,594) of proteomics to provide genetic predictors of the IL-1 family^23,24^ and the International Lung Cancer Consortium (ILCCO, n = 11,348 cases, and n = 15,861 controls) for lung cancer in people of European ancestry^25^. We primarily assessed the effects of genetically predicted IL-1α, IL-1β, IL-1Ra, IL-1Racp, IL-18, and IL-18BP with lung cancer and its subtypes of LUAD and LUSC, because LUAD is more common than LUSC in people of European ancestry^26,27^. We secondarily examined the associations of IL-1R1, IL-18Rα, IL-37, IL-36α, IL-36β, and IL-36γ with lung cancer and its subtypes, because those exposures have seldom been investigated previously, possibly with limited anti-tumor effects.

## Methods

### Study design and participants

This study used two-sample MR to assess the effects of circulating levels of IL-1 family members/receptors on lung cancer and its subtypes (LUAD and LUSC). Supplementary Figure S2 shows the flowchart of the study design. Genetic predictors of *cis* protein quantitative trait loci (*cis-*pQTLs, i.e. the functional genetic variants that affect protein abundance with little or no attenuated effect on messenger RNA or ribosome levels concerning *trans*-pQTLs^24,28^) for IL-1 family were obtained from summary statistics of up to 11,594 European participants in two proteomics GWAS^23,24^ with an average age at around 47 years. The proteomics GWAS was adjusted for age, sex, body mass index^23^ and time between blood draw and processing^24^, and the first three or ten genetic principal components. We applied these identified instruments to summary statistics in ILCCO, which is the largest available lung cancer GWAS^25^. The ILCCO recruited 27,209 participants of ~ 95.8% European ancestry, with more than 66% aged 50 + years and ~ 26.3% women. Table 1 presents a detailed summary of the included studies, their participants, the assays, the genotyping platforms, and genomic control used in each study.

**Table 1:** Study details for the two genome-wide association studies (GWAS) for proteomics and the International Lung Cancer Consortium (ILCCO) study.

Study	*N* (% of European descent)	*N* (proteins assessed or lung cancer cases)	Phenotype definition	Mean age of years	Sex (female, %)	Adjusted for	Genotyping platform	*cis*-pQTLs	Data source
YFS and FINRISK survey^23^	Up to 8293 (100)	48	Bio-Plex Pro Human Cytokine 27/21-plex Assay, with SD unit, including IL-1β, IL-1Ra, IL-18	~ 49	N.A	Age, sex, body mass index, and the first ten genetic principal components	Illumina HumanHT-12 v.4 Expression BeadChip	GWAS catalog (July 6, 2015) for complex trait-associated variants with 1 Mb window from the cytokine lead variants with $P < 1.2 \times 10^{ - 9}$	https://grasp.nhlbi.nih.gov/FullResults.aspx
INTERVAL (Healthy participants)^24^	3301 (100)	3622	SOMAscan platform, with log-transformed relative fluorescent unit, including IL-1α, IL-1R1, IL-1Racp, IL-18BP, IL-18Rα, IL-36α, IL-36β, IL-36γ, IL-37	~ 44	1,614 (~ 48.9)	Age, sex, duration between blood draw and processing, and the first three principal components	Affymetrix Axiom UK Biobank genotyping array at Affymetrix	The leading genetic variant in the region located within 1 Mb of the gene's canonical transcription start site of the genes encoding the protein. pQTLs lying outside of this region were defined as *trans*	http://www.phpc.cam.ac.uk/ceu/proteins/
ILCCO^25^	27,209 (~ 95.8)	11,348	Usually based on ICD-O-2 or ICD-O-3	50 + (> 66.3%)	~ 26.3%	Study-specific covariates	Illumina platform	N.A.	Ilcco.iarc.fr
IARC GWAS	6324 (100)	2533			1875 (29.7)				
NCI GWAS	11,449 (100)	5713			2148 (18.8)				
MDACC GWAS	2284 (~ 50)	1150			985 (43.1)				
ICR GWAS	7152 (100)	1952			N.A.				

*GWAS* genome-wide association study, *NA* not applicable, *IL-1β* interleukin-1β, *IL-1Ra* interleukin-1 receptor antagonist, *IL-18* interleukin-18, *IL-1α* interleukin-1α, *IL-1R1* interleukin-1 receptor type 1, *IL-1Racp* interleukin-1 receptor accessary protein, *IL-18BP* interleukin-18 binding protein, *IL-18Rα* interleukin-18 receptor 1, *IL-36α/β/γ* interleukin-36 α/β/γ, *IL-37* interleukin-37, *cis-pQTLs* circulating protein quantitative trait loci.

### Genetic predictors of IL-1 family members/receptors

We selected pQLTs strongly associated with 12 IL-1 family members [i.e., IL-1α (log-transformed relative fluorescent unit, log(RFU)^29^; i.e., RFU is the proportional to the amount of target protein in the initial sample, as informed by a standard curve generated for each protein-SOMAmer pair)], IL-1β [standard-derivation (SD, ~ 2.2 pg/ml in YFS)], IL-1Ra [SD, ~ 3.5 ng/ml in FINRISK2002], IL-18 [SD, ~ 38.0 pg/ml in YFS], IL-36α [log(RFU)], IL-36β [log(RFU)], IL-36γ [log(RFU)], and IL-37 [log(RFU)]) and IL-1 receptors (IL-1R1 [log(RFU)], IL-1Racp [log(RFU)], IL-18Rα [log(RFU)], and IL-18 binding protein [IL-18BP, log(RFU)] at a threshold of $P < 5 \times 10^{ - 6}$, which is often used to highlight those “suggestive” variants (as shown in Supplementary Table S1)^30^. Only annotated pQTLs (i.e., with accurate identification and description^31^) in RegulomeDB (https://regulomedb.org/regulome-search/)^32^ database were included to reduce random variability. We further identified *cis*-pQTLs by excluding pQTLs with expression quantitative trait loci (eQTLs)^28^ in either RegulomeDB or PhenoScanner (http://www.phenoscanner.medschl.cam.ac.uk/)^33^ to avoid unknown pleiotropy, although surrogate instruments for exposures are valid in MR^22^. We selected independent *cis*-pQTLs ($r^{2} < 0.05$) based on the 1000 genomes European reference panel obtained from LDlink (https://ldlink.nci.nih.gov/). To ensure we selected unconfounded instruments only affected lung cancer via the relevant exposure, we excluded *cis*-pQTLs associated with possible exposure-outcome confounders (e.g. age, smoking, socioeconomic position and platelets^34^), targeting proteins of drugs for lung cancer treatment (e.g., tyrosine-protein kinase, as shown in Supplementary Table S2) and competing events related to death in the curated genotype to phenotype cross-reference PhenoScanner at a conventional genome-wide significance threshold of $5 \times 10^{ - 8}$. Noticeably, *cis*-pQLTs associated with proteins targeted by drugs for lung cancer treatment based on previously published studies would be excluded as the alternative pathways to lung cancer might be open among survivors, resulting in invalid instruments. The resulting list of these instrumental *cis*-pQTLs for each of the IL-1 family members/receptors are given in Supplementary Table S3.

### Genetic associations with lung cancer

We used summary effect estimates and corresponding standard errors of the genetic association with lung cancer in ILCCO^25^, in which lung cancer was classified based on the international classification of diseases for oncology (ICD-O-2 or ICD-O-3) as LUAD, LUSC or mixed cancers (i.e., overlapping histological types). Of these, 3442 LUAD cases with 14,894 controls and 3275 LUSC cases with 15,038 controls were identified. Genetic variants with poor quality (e.g., RSQR < 0.30 with MaCH or an information measure is < 0.40 with IMPUTE2) were excluded from MR analyses as described elsewhere^25^. We replaced them with available proxy variants, identified as a high-linkage disequilibrium ($r^{2} > 0.80$) variant using LDlink.

### Statistical analysis

#### Effects of IL-1 family members/receptors on lung cancer

We excluded *cis-*pQTLs with an F-statistic less than the rule of thumb of 10 to avoid weak instrument bias. F-statistics were approximated by the square of SNP on exposure divided by the square of its standard error^35^. We harmonized summary estimates for each IL-1 family member/receptor and lung cancer by flipping for non-palindromic strand variants (i.e., *cis*-pQTLs with different letters of a pair of G/T or C/A for exposure and outcome data) and removing palindromic and incompatible variants (i.e., *cis*-pQTLs with different alleles for both exposure and outcome, e.g., A/G alleles for the exposure and A/C alleles for the outcome) via TwoSampleMR::harmonise_data(), and then constructed equally weighted polygenic risk scores (PRS). Compared with PRS constructed using the individual-level data, the equally weighted PRS constructed based on summary-level data is an equivalent approach to avoid weak instruments (Supplementary Materials)^36^. We estimated PRS-specific Wald estimates with the standard error derived via the delta method^22^. Cochran’s Q-statistic with *P* < 0.05 was considered to be heterogeneity for the causal effect estimates^37^.

In sensitivity analyses, we used fixed- and random-effect with inverse-variance weighting^38^, a weighted median^39^, and MR Egger^40^ for estimating total effects. A weighted median method allows up to 50% of the information to be from invalid instruments and provides a consistent effect estimate^39^. MR Egger, assuming the instrument strength is independent of direct effects (InSIDE), allows for heterogeneity in causal effects. A nonzero MR Egger intercept with *P* < 0.05 indicates that some identified *cis*-pQTLs may be acting other than via the exposure^40^.

Finally, we used multivariable MR based on robust weighted linear regression to estimate the direct effects of IL-1 family members/receptors on lung cancer risk (overall, LUAD and LUSC), after adjusting for genetically predicted effects of other IL-1 family members/receptors^41^, considered as IL-1, IL-18, IL-36 and IL-1Rs. We assessed multivariable instrument strength using the conditional F-statistic, which gives a lower bound assuming no correlation between exposures, and conducted sensitivity analysis by removing those exposures with lowest pairwise conditional F-statistics to evaluate the robustness of the estimated effects^42,43^. Similar to F-statistic in univariable MR, *cis*-pQTLs with a conditional F-statistic less than 10 is considered as a weak instrument^42,43^. To assess multivariable instrument pleiotropy, we used multivariable MR Egger orientated on the exposure of interest and the modified Q-statistic^42,43^, which gives an upper bound assuming no correlation between exposures.

### Power analysis

We used the online calculator for power calculation of MR studies (http://cnsgenomics.com/shiny/mRnd/)^44^. In the original GWAS for proteomics^23,24^, the median variation explained in protein levels by pQTLs was 5.8% (interquartile range: 2.6–12.4%). Assuming all identified *cis*-pQTLs only explained the median value of the total variance (i.e., 5.8%), an odds ratio of 0.87 could be detected at 80% power and 0.05 alpha.

### Statistical software

We performed analyses using R version 3.6.2 software platform (R Foundation for Statistical Computing) with the TwoSampleMR (https://mrcieu.github.io/TwoSampleMR/index.html), robustMVMR (https://cran.r-project.org/web/packages/robustMVMR/index.html), ieugwasr (https://mrcieu.github.io/ieugwasr/index.html), and HalopR (https://github.com/cluoma/haloR) packages. Software codes and data are available on request. We reported two-sided *P* values at the Bonferroni-corrected threshold of 0.05/No. of exposure/No. of outcomes = 0.05/12/3 = 0.0014 to address multiple testing issues, and *P* value between 0.0014 and 0.05 was considered as suggestive of a potential association. We adhered STROBE-MR: Guidelines for strengthening the reporting of Mendelian randomization studies for reporting our results^45^.

### Ethics approval and consent to participate

This analysis of publicly available data does not require ethical approval.

## Results

We obtained 220 candidates pQTLs (including two overlap variants: rs61335305 for IL-1β and IL-1Ra and rs74480769 for IL-36α and IL-37, Supplementary Table S1) as instruments for 12 IL-1 family members/receptors from the two proteomics GWAS, and excluded 9 pQTLs that were not well-annotated in the RegulomeDB database (Supplementary Table S4). Of the remaining 211 pQTLs, 81 were associated with an expression quantitative trait loci (eQTL) in either the RegulomeDB (Supplementary Table S5) or PhenoScanner (Supplementary Table S6) databases and were excluded, leaving 130 *cis*-pQTLs. We further excluded 3 *cis*-pQTLs that were associated with competing events related to death (Supplementary Table S7). No *cis*-pQTLs associated with target proteins of drugs or the potential confounders (e.g., smoking) for lung cancer were found at a conventional genome-wide association threshold of $5 \times 10^{ - 8}$. Of these 127 *cis*-pQTLs (including an overlap *cis*-pQLT of rs61335305 for both IL-1β and IL-1Ra, which was only used for robust multivariable MR analyses), we excluded 23 palindromic and 47 incompatible instruments concerning those for lung cancer in ILCCO. No *cis*-pQTLs due to poor quality in ILCCO were excluded. Thus, a total of 79 valid *cis*-pQTLs were included in the final analysis, as shown in Supplementary Table S3. The average F-statistic varied from 15.15 to 37.86, indicating instrument strength.

### Total effects of IL-1 and IL-1Rs on lung cancer

**Figure 1:**
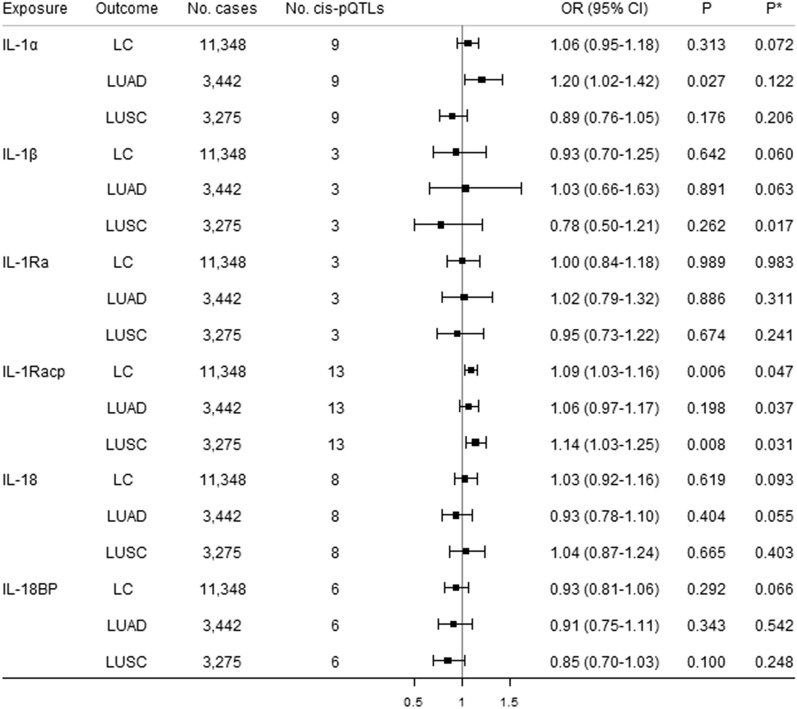
Causal estimates of genetically predicted IL-1α, IL-1β, IL-1Ra, IL-1Racp, IL-18, and IL-18BP with lung cancer (LC) and its subtypes [lung adenocarcinoma (LUAD) and squamous cell lung cancer (LUSC)] using univariable MR analyses. *Cis*-pQTLs is *cis* protein quantitative loci. P* indicates the Q-statistic test.

**Figure 2:**
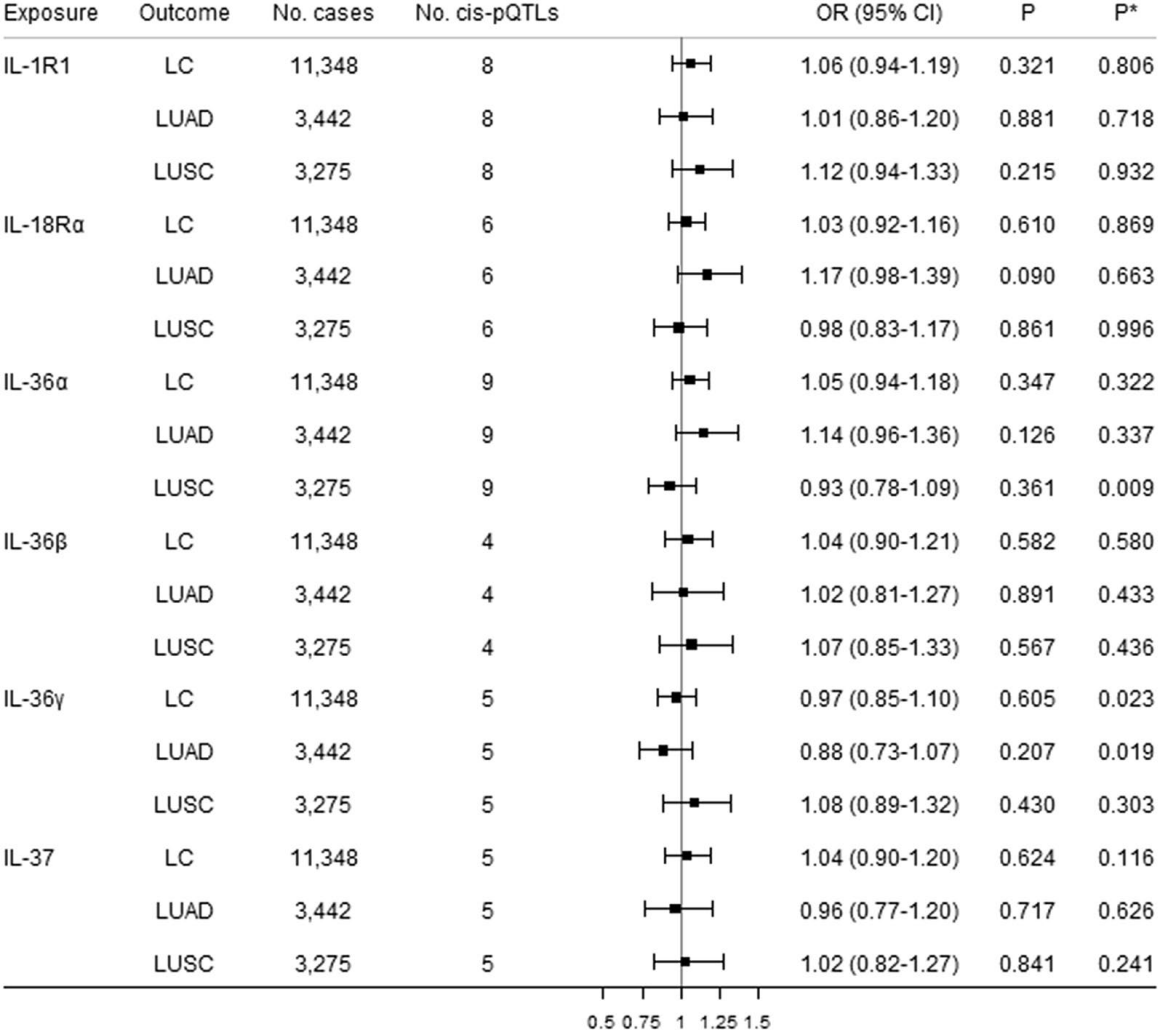
Causal estimates of genetically predicted IL-1R1, IL-18Rα, IL-36a, IL-36β, IL-36γ, and IL-37 with lung cancer (LC) and its subtypes [lung adenocarcinoma (LUAD) and squamous cell lung cancer (LUSC)] using univariable MR analyses. *Cis*-pQTLs is *cis* protein quantitative loci. P* indicates the Q-statistic test.

Figures 1 and 2 show the causal estimates for the 12 genetically predicted IL-1 family members/receptors with lung cancer using univariable MR. No significant associations of IL-1 family members/receptors and lung cancer were observed after correction for multiple testing. We noted suggestive associations of genetically predicted higher circulating IL-1Racp with a 9% increased risk of lung cancer, and a 14% increased risk of LUSC. However, the Q-statistic indicated possible heterogeneity (*P* = 0.047 for lung cancer and *P* = 0.031 for LUSC). Leave-one-out analysis showed that the *cis*-pQTLs rs67249092 had a relatively strong influence on the causal estimates for IL-1Racp on both lung cancer and LUSC (Supplementary Figures S3 and S4). There was a suggestive association of genetically predicted higher circulating IL-1α with a 20% increased risk of LUAD, without heterogeneity identified. No suggestive associations were observed for other IL-1 family members/receptors with lung cancer and its subtypes. The MR Egger intercepts showed no evidence of directional pleiotropy (Supplementary Table S8). Sensitivity analyses yielded consistent results (Supplementary Table S8).

### Direct effects of IL-1 and IL-1Rs on lung cancer

**Figure 3:**
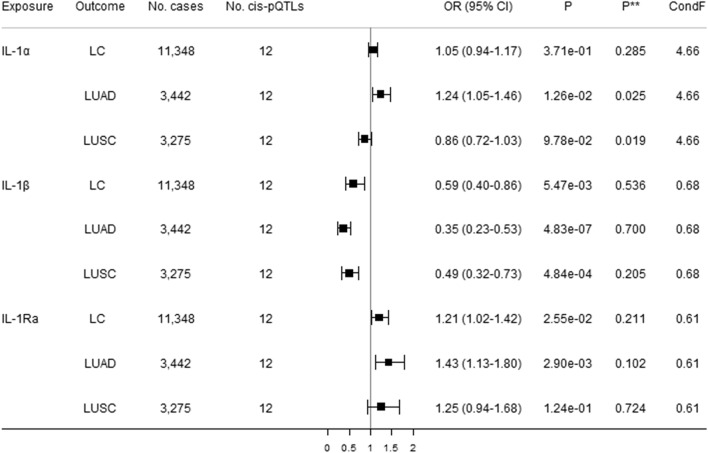
Direct causal estimates of genetically predicted IL-1α, IL-1β, and IL-1Ra with lung cancer (LC) and its subtypes [lung adenocarcinoma (LUAD) and squamous cell lung cancer (LUSC)] using robust multivariable MR analyses. *Cis*-pQTLs is *cis* protein quantitative loci. P** indicates a pleiotropy test based on the MVMR Egger. ConF indicates the overall conditional F-statistic. The modified Q-statistic for LC, LUAD, and LUSC are respectively 8.27 (*P* = 0.607), 6.32 (*P* = 0.788), and 9.27 (*P* = 0.507).

**Figure 4:**
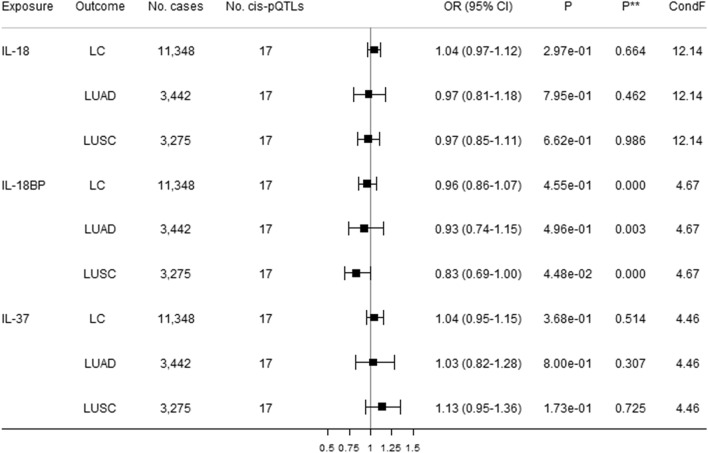
Direct causal estimates of genetically predicted IL-18, IL-18BP, and IL-37 with lung cancer (LC) and its subtypes [lung adenocarcinoma (LUAD) and squamous cell lung cancer (LUSC)] using robust multivariable MR analyses. *Cis*-pQTLs is *cis* protein quantitative loci. P** indicates a pleiotropy test based on the MVMR Egger. ConF indicates the overall conditional F-statistic. The modified Q-statistic for LC, LUAD, and LUSC are respectively 28.00 (*P* = 0.022), 13.53 (*P* = 0.562), and 18.99 (*P* = 0.214).

**Figure 5:**
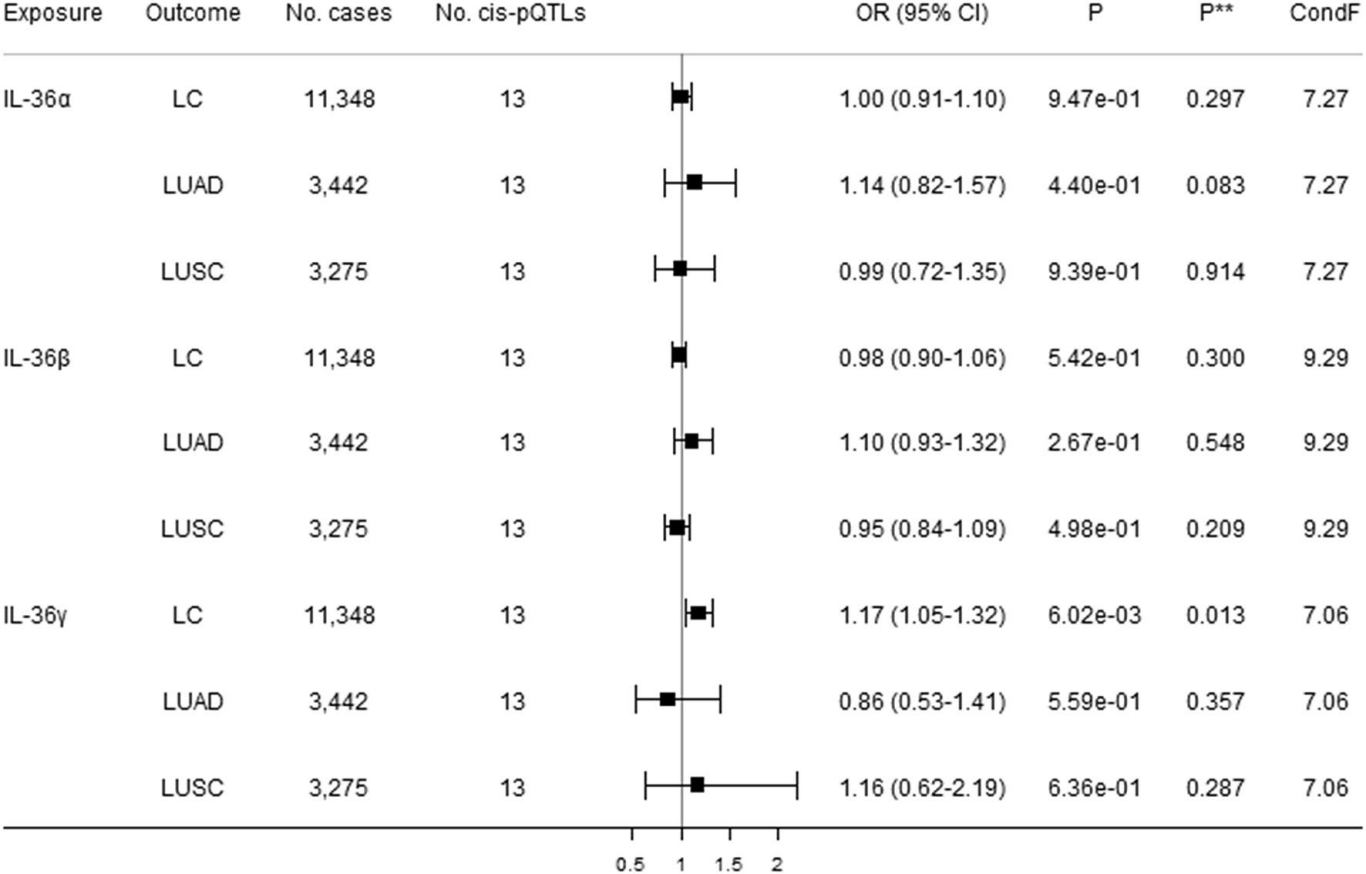
Direct causal estimates of genetically predicted IL-36α, IL-36β, and IL-36γ with lung cancer (LC) and its subtypes [lung adenocarcinoma (LUAD) and squamous cell lung cancer (LUSC)] using robust multivariable MR analyses. *Cis*-pQTLs is *cis* protein quantitative loci. P** indicates a pleiotropy test based on the MVMR Egger. ConF indicates the overall conditional F-statistic. The modified Q-statistic for LC, LUAD, and LUSC are respectively 20.16 (*P* = 0.043), 14.09 (*P* = 0.228), and 6.14 (*P* = 0.864).

Figure 3 shows direct causal estimates of genetically predicted higher circulating IL-1α, IL-1β, and IL-1Ra with lung cancer and its subtypes (i.e., LUAD and LUSC) using robust multivariable MR. There was a suggestive association of IL-1α with a 24% increased risk of LUAD after adjusting for genetically predicted effects of IL-1β and IL-1Ra. MVMR Egger indicated possible pleiotropy but not the modified Q statistic. Genetically predicted higher circulating IL-1β was inversely associated with LUAD and LUSC, and was suggestively associated with decreased lung cancer risk after adjusting for genetically predicted effects of IL-1α and IL-Ra, without pleiotropy identified. Genetically predicted higher circulating IL-1Ra was suggestively positively associated with increased risk of both lung cancer and LUAD after adjusting for genetically predicted effects of IL-1α and IL-1β. MVMR Egger and the modified Q-statistic did not indicate pleiotropy. Figures 4 and 5 show direct causal estimates of genetically predicted higher circulating IL-18 and IL-36 subfamily members with lung cancer and its subtype using robust MVMR analyses. There were suggestive associations of increased IL-36γ with increased 17% increased lung cancer, and increased IL-18BP with a 17% decreased LUAD. The MVMR Egger and modified Q-statistic suggested possible pleiotropy.

### Direct effects of 12 IL-1 family members/receptors on lung cancer

Supplementary Figures S5–S7 show direct causal estimates of genetically predicted high circulating IL-1 family members/receptors with lung cancer and its subtypes separately using robust MVMR analyses after adjusting for genetically predicted effects of all available other IL-1 family members/receptors. A suggestive positive association of IL-1α with LUAD was observed after adjusting for genetically predicted effects of other IL-1 family members/receptors, with possible pleiotropy identified (*P* = 0.027). No other associations were observed of IL-1 family with lung cancer (including LUAD and LUSC). The MVMR Egger and modified Q-statistic did not indicate pleiotropy.

Supplementary Table S9 presents the pairwise conditional F-statistic for testing instrument strength under each model using robust multivariable MR analyses. The sensitivity analysis after removing IL-1β (overall conditional F-statistic = 0.68 in Model 1 and 0.62 in Model 4), IL-36α (overall conditional F-statistic = 7.27 in Model 3 and 1.10 in Model 4), and IL-37 (overall conditional F-statistic = 4.46 in Model 2 and 1.80 in Model 4) yielded similar results, as shown in Supplementary Table S10. MVMR Egger and the modified Q-statistic suggested possible pleiotropy.

## Discussion

### Principal findings

This first MR study comprehensively assessing the causal roles of IL-1 family members/receptors in lung cancer and its subtypes, provided suggestive evidence for associations of increased IL-1Racp with increased lung cancer/LUSC, and increased IL-1α with increased LUAD. Our findings show little genetic evidence for the association of IL-1β and lung cancer, inconsistent with the randomized controlled trial^2^. There was also suggestive evidence for possible associations of IL-1β, IL-1Ra, IL-36γ with lung cancer, IL-1α/β, IL-1Ra with LUAD, and IL-1β, IL-18BP with LUSC, perhaps via other IL-1 family members/receptors. This adds additional randomized evidence showing that IL-1Racp may partially operate on lung cancer via especially IL-1α/β and IL-1Ra possibly by suppressing innate responses^46,47^.

### Comparison with other studies

Our finding on IL-1β is inconsistent with the CANTOs trial^2^, where suppressing IL-1β reduced lung cancer incidence and mortality. However, it was an exploratory finding and was likely to be driven by treatment related competing risk of other events during follow-up, especially in patients with previous myocardial infarction and an inflammatory state. The interleukin-1 signaling pathway is activated by the binding of IL-1 family members to their receptors, triggering a cascade of inflammatory mediators, chemokines, and cytokines^46,47^. These have diverse functions, ranging from anti-tumorigenesis to pro-tumorigenesis, anti-atherosclerosis, remodeling left ventricular function, and boosting innate immune response, which further leads to the development of corresponding therapies for several diseases, including cancer, cardiovascular diseases, and autoimmune diseases^9,11–13,48–50^. Our findings are also inconsistent with a previous MR study showing increasing IL-18 appears to protect against lung cancer^51^. The discrepancy might be due to including genetic predictors of both *cis*-pQLTs and *trans*-pQTLs as instruments, in which *trans*-pQLTs may affect lung cancer via unknown pleiotropy, although pQTLs only make a small difference to lifetime IL-18^28^. IL-1Ra is a target of the rheumatoid arthritis treatment anakinra, possibly acting by raising testosterone in men^52–54^. However, genetic instruments for IL-1Ra which is drugged by anakinra were associated with lung cancer/LUAD only after adjustment for the genetically predicted effects of IL-1α and IL-1β in this study. The genetic variants previously used to mimic effects of anakinra did not cause lung cancer^14^.

### Strengths and limitations

Our current study has two notable strengths. First, our findings are less likely to suffer from selection bias due to the enrolled participants with an average age at ~ 50 years (i.e., few deaths would have occurred before the recruitment) and the stringent selection of the *cis*-pQLTs as instruments. Second, we identified the possible source of conditionally weak instrument bias via the pairwise conditional F-statistic based on a robust multivariable MR method (which accounts for heteroskedasticity, autocorrelation, and the presences of outliers)^41,42,55,56^, and found that its effect seemed minimal.

Nevertheless, our study has several limitations. First, MR requires stringent assumptions (i.e., relevance, independence, and exclusion-restriction). To satisfy these assumptions, we identified *cis*-pQTLs that strongly predicted IL-1 family members/receptors in two large-scale GWAS for proteomics^23,24^. We also restricted genetic variants to well-genotyped *cis*-pQTLs, and then searched curated genotype to phenotype databases comprehensively to identify potential pleiotropy, and finally excluded the corresponding *cis*-pQLTs to reduce possible bias. We additionally examined the validation of these selected *cis*-pQTLs using the well-established IL-1Ra-rheumatoid arthritis association based on the latest GWAS studies. Supplementary Table S11 provides that genetically predicted IL-1Ra was associated with a decreased risk of rheumatoid arthritis. Even though heterogeneity was observed in a few analyses, no pleiotropy was indicated by MR Egger suggesting findings are less likely to be biased estimates^57^. In addition, we limited our study to people of European descant using GWAS with genetic control so that the estimated effects were less likely to be affected by population stratification. As a consequence, our findings reflect the population-level effects of European descent, which may not apply to other populations, although causes are usually consistent they may not be relevant in different populations^58^. Second, winner’s curse may arise as we identified the independent *cis*-pQTLs as instruments for IL-1 family members/receptors at a suggestive significance level of $5 \times 10^{ - 6}$ with either the log-transformed or standard deviation unit. In this case, the observed results would bias towards the null due to the overestimated *cis*-pQTLs-exposure associations^59^. Nevertheless, we could repeat the analysis in the UK Biobank once it accumulates enough lung cancer cases^60^. Third, despite selecting strongly associated *cis*-pQTLs, these identified instruments did not explain much total variation in IL-1 family members/receptors, yielding underpowered results even if equally weighted PRS was employed and had been shown to be an equivalent approach to that in individual-level data^36^. Fourth, due to the limited understanding of the function of *cis-*pQLTs for predicting IL-1 family members/receptors, unknown pleiotropic effects may exist. Fifth, canalization buffering genetic factors may happen; however, the effect of this is unknown. Sixth, due to using summary statistics we could not conduct analysis by sex.

### Public health implications

Our findings suggest that decreasing IL-1Racp, with relevance to pharmaceutical interventions, might reduce the risk of lung cancer in the general population, perhaps via other IL-1 family members/receptors, especially IL-1α/β and IL-1Ra. However, from a public health perspective, our findings should be interpreted cautiously. First, the suggestive association between increased IL-1Racp and increased lung cancer could be a chance finding at *P* = 0.05 level, although consistent results were obtained using different MR techniques with a relatively large effect. Second, the underlying interleukin-1 signaling pathway remains unclear. The effect of IL-1Racp in lung cancer may work via IL-1α/β and IL-1Ra (Supplementary Figure S1) by sharping the tumor microenvironment, leading to an suppressed immune responses^47^. Third, histological type-specific effects of IL-1α/β, IL-1Ra, and IL-1Racp were observed especially after adjusting for genetically predicted effects of other IL-1 family members/receptors (Figs. 3, 4, 5 and Supplementary Figures S3–S5), even if there is no known reason to expect such effect modification especially in the prevention of lung cancer. However, clarifying the role of IL-1Racp and its interplay with other IL-1 family members/receptors by histological types of lung cancer using network analyses when large-scale GWAS become available may provide additional insights^61,62^. Furthermore, better understanding with randomized controlled trials is further required to decipher the biological pathways underpinning associations of IL-1 family members/receptors with lung cancer.

## Conclusion

Our comprehensive MR study suggests that IL-1Racp might cause lung cancer, perhaps via IL-1α/β and IL-1Ra. Clarifying the role of IL-1 family members/receptors and their underlying mechanisms concerning histological types of lung cancer, with relevance to pharmaceutical interventions or identifying those effective interventions for lung cancer, is worthwhile.

## Supplementary Information

Supplementary Figures.

Supplementary Information.

Supplementary Tables.

## Data Availability

The summary statistics for genetic associations of IL-1α, IL-1R1, IL-1Racp, IL18BP, IL18Rα, IL-36α, IL-36β, IL-36γ, IL-37 are available in the INTERVAL study (http://www.phpc.cam.ac.uk/ceu/proteins/). The summary statistics for genetic associations of IL-1β, IL-1Ra, IL-18 are available in YFS and FINRISK survey (https://grasp.nhlbi.nih.gov/FullResults.aspx). The summary statistics for genetic associations of lung cancer and its subtypes (lung adenocarcinoma and squamous cell lung cancer) are available in the International Lung Cancer Consortium (Ilcco.iarc.fr). The annotated QTLs are available in RegulomeDB (https://regulomedb.org/regulome-search/) and PhenoScanner (http://www.phenoscanner.medschl.cam.ac.uk/). The 1000 genomes European reference panel is available via LDlink (https://ldlink.nci.nih.gov/).

## Code availability

Software codes and data are available on request.
