# Supplementary material for: Mendelian randomization study of interleukin (IL)-1 family and lung cancer
Source: Sci Rep. 2021 Sep 2;11:17606. doi: 10.1038/s41598-021-97099-5 (PMC8413403; doi:10.1038/s41598-021-97099-5)
Supplement: Supplementary file 1 — Supplementary Figures. [file 41598_2021_97099_MOESM1_ESM.docx]

**Supplementary Figures of “Mendelian randomization study of interleukin (IL)-1 family and lung cancer”**

Zhao Yang, C. Mary Schooling, Man Ki Kwok

Contents

**Figure S1.** The putative biological mechanism linking available interleukin (IL)-1 family members/receptors with lung cancer. 2

**Figure** S**2.** Study design flowchart. *Cis*-pQTLs: *cis* protein quantitative trait loci; LC: lung cancer; LUAD: lung adenocarcinoma; LUSC: squamous cell lung cancer; MVMR: multivariable Mendelian randomization. 3

**Figure S3.** Leave-one-out analyses on causal estimates of genetically predicted IL-1Racp with lung cancer based on random-effects inverse-variance weighting approach 4

**Figure S4.** Leave-one-out analyses on causal estimates of genetically predicted IL-1Racp with lung squamous cell lung cancer based on random-effects inverse-variance weighting approach 5

**Figure S5.** Direct causal estimates of genetically predicted IL-1 family members/receptors with lung cancer using robust multivariable MR analyses. *Cis*-pQTLs is *cis* protein quantitative loci. *P*** indicates a pleiotropy test based on the MVMR Egger. ConF indicates the overall conditional F-statistic. The modified Q-statistic is 76.15 with *P*=0.078. 6

**Figure S6.** Direct causal estimates of genetically predicted IL-1 family members/receptors with lung adenocarcinoma using robust multivariable MR analyses. *Cis*-pQTLs is *cis* protein quantitative loci. *P*** indicates a pleiotropy test based on the MVMR Egger. ConF indicates the overall conditional F-statistic. The modified Q-statistic is 49.55 with *P*=0.830. 7

**Figure S7.** Direct causal estimates of genetically predicted IL-1 family members/receptors with squamous cell lung cancer using robust multivariable MR analyses. *Cis*-pQTLs is *cis* protein quantitative loci. *P*** indicates a pleiotropy test based on the MVMR Egger. ConF indicates the overall conditional F-statistic. The modified Q-statistic is 64.87 with *P*=0.311.

**Supplementary Figures**

IL-1β (Canakinumab)

+

IL-36α

IL-36β

IL-18Rα

IL-18Rβ

+

IL-1Racp

Toll-IL1-receptor domain + MyD88

↓

Activation of transcription factors (e.g., Ap-1 and NF-κB)

IL-37

Signalling in lung cancer

IL-1α (MABp1)

IL-1Ra (Anakinra)

IL-36γ

IL-18BP

IL-18

IL-1R2

IL-1R1

IL-36R

+

Cytokines

Receptors

Receptor antagonists

Co-receptors

# **Figure S1.** The putative biological mechanism linking available interleukin (IL)-1 family members/receptors with lung cancer.

Identify pQTLs that associated with 12 IL-1 family members/receptors

- YFS and FINRISK study (up to 8,293)
- INTERVAL study (n = 3,301)

Exclude *cis*-pQTLs that associated

- weak instruments (F < 10)
- exposure-outcome confounders (e.g., smoking, socioeconomic position, platelet, et al.)
- targeted proteins of drugs for lung cancer treatment (e.g., programmed death protein 1, et al)
- competing events caused death

Lung cancer genotyping data

- ILCCO study
- LC: 11,348 cases and 15,861 controls
- LUAD: 3,442 cases and 14,894 controls
- LUSC: 3,275 cases and 15,038 controls

Harmonize those identified *cis*-pQTLs by

- removing poor quality variants
- removing incompatible variants
- flipping strand-ambiguous variants
- identifying proxy variants

Estimate causal effects

- Total effect: PRS-specific Wald estimate
- Direct effect: Robust multivariable MR

Conduct sensitivity analyses

- Inverse-variance weighting with fixed and random effects
- Weighted median
- MR Egger regression

Test for pleiotropy

- MR Egger intercept
- MVMR Egger intercept
- Modified Q-statistic

Identified well-annotated pQTLs

- RegulomeDB database

Identify *cis*-pQTLs

- RegulomeDB and PhenoScanner database

# **Figure** S**2.** Study design flowchart. *Cis*-pQTLs: *cis* protein quantitative trait loci; LC: lung cancer; LUAD: lung adenocarcinoma; LUSC: squamous cell lung cancer; MVMR: multivariable Mendelian randomization.


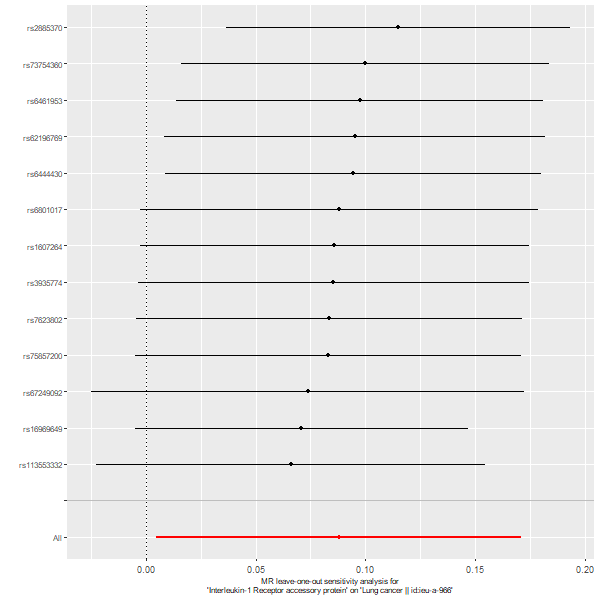


# **Figure S3.** Leave-one-out analyses on causal estimates of genetically predicted IL-1Racp with lung cancer based on random-effects inverse-variance weighting approach


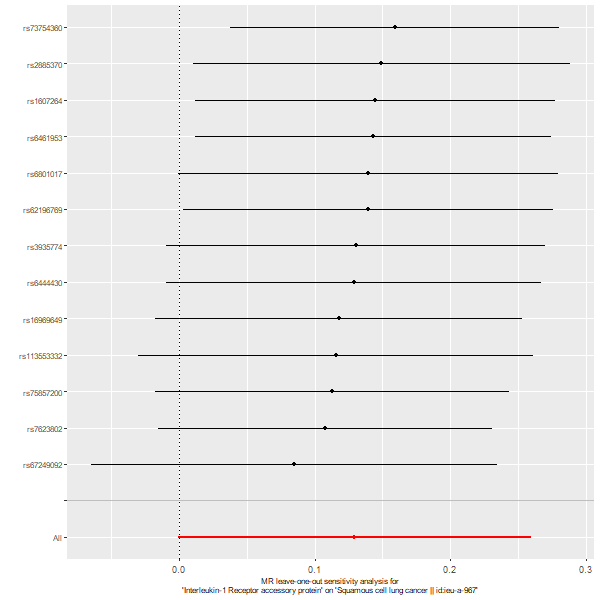


# **Figure S4.** Leave-one-out analyses on causal estimates of genetically predicted IL-1Racp with lung squamous cell lung cancer based on random-effects inverse-variance weighting approach


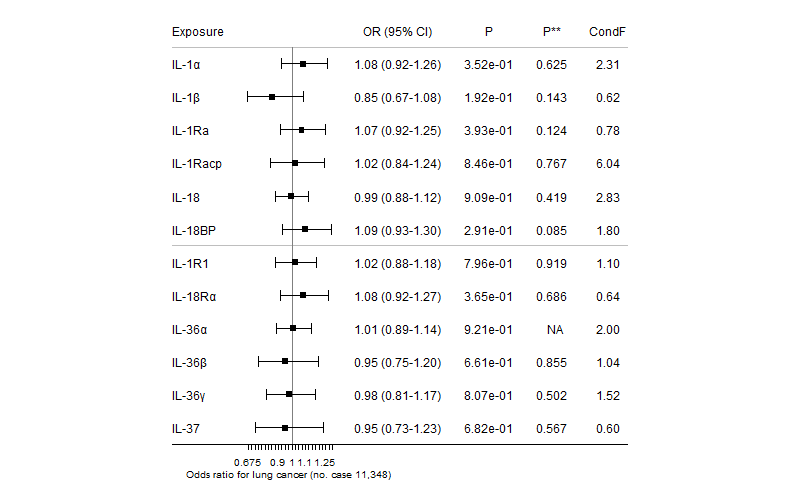


# **Figure S5.** Direct causal estimates of genetically predicted IL-1 family members/receptors with lung cancer using robust multivariable MR analyses. *Cis*-pQTLs is *cis* protein quantitative loci. *P*** indicates a pleiotropy test based on the MVMR Egger. ConF indicates the overall conditional F-statistic. The modified Q-statistic is 76.15 with *P*=0.078.


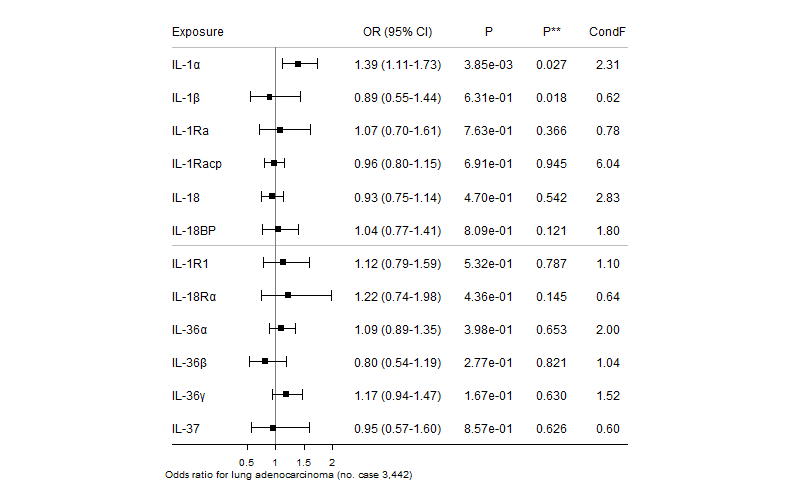


# **Figure S6.** Direct causal estimates of genetically predicted IL-1 family members/receptors with lung adenocarcinoma using robust multivariable MR analyses. *Cis*-pQTLs is *cis* protein quantitative loci. *P*** indicates a pleiotropy test based on the MVMR Egger. ConF indicates the overall conditional F-statistic. The modified Q-statistic is 49.55 with *P*=0.830.


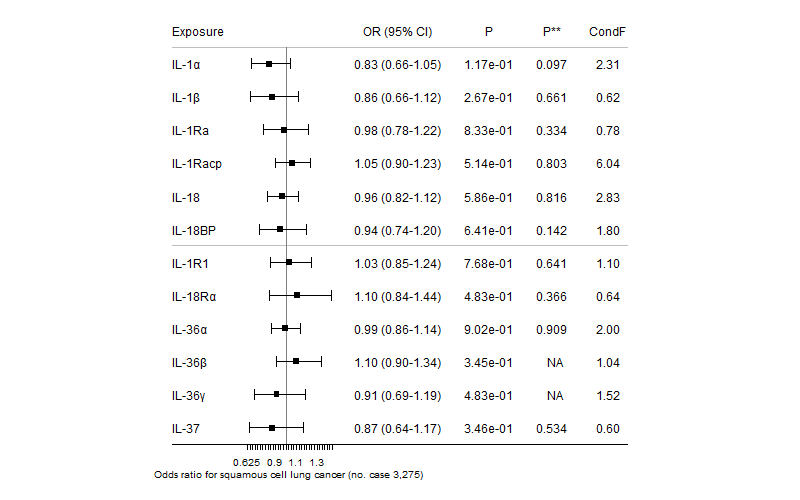


# **Figure S7.** Direct causal estimates of genetically predicted IL-1 family members/receptors with squamous cell lung cancer using robust multivariable MR analyses. *Cis*-pQTLs is *cis* protein quantitative loci. *P*** indicates a pleiotropy test based on the MVMR Egger. ConF indicates the overall conditional F-statistic. The modified Q-statistic is 64.87 with *P*=0.311.
