# Supplementary material for: Mendelian randomization study of interleukin (IL)-1 family and lung cancer
Source: Sci Rep. 2021 Sep 2;11:17606. doi: 10.1038/s41598-021-97099-5 (PMC8413403; doi:10.1038/s41598-021-97099-5)
Supplement: Supplementary file 2 — Supplementary Information. [file 41598_2021_97099_MOESM2_ESM.docx]

**Supplementary materials of “Mendelian randomization study of interleukin (IL)-1 family and lung cancer”**

Zhao Yang, C. Mary Schooling, Man Ki Kwok

**Equally weighted polygenic risk score (PRS) based on summary-level estimates**

Suppose a dataset with information on the risk factor X, outcome Y, and the independent instrumental variants IVs ($G_{1},\ldots,G_{K}, k=1,\ldots,K$). Let $\hat{\beta}_{Xk}$ and $\sigma_{Xk}$ denote the genetic association and its standard error between the risk factor X and IVs, and let $\hat{\beta}_{Yk}$ and $\sigma_{Xk}$ be the genetic association and its standard error between the outcome Y and IVs. The weighted PRS can be constructed as follows, $Z=\sum_{k} w_{k}G_{k}$, where $w_{k}$ is the weight and $G_{k}$ is the allele score. Based on the results derived by Burgess et al.^1^, the estimate ($\hat{\beta}_{XY}$) of the weighted PRS on the outcome Y based on the two-sample summary-level data is

$$\hat{\beta}_{XY}=\frac{cov\left( Y,Z \right)}{cov\left( X,Z \right)}=\frac{\sum_{k} {w_{k}\hat{\beta}_{Yk}}/{\sigma_{Yk}^{2}}}{\sum_{k} {w_{k}\hat{\beta}_{Xk}}/{\sigma_{Yk}^{2}}}\mathrm{and}$$

$$\mathrm{se}\left( \hat{\beta}_{XY} \right)\approx\sqrt{\frac{\sum w_{k}^{2}/\sigma_{Yk}^{2}}{\left( \sum w_{k}\hat{\beta}_{Xk}/\sigma_{Yk}^{2} \right)^{2}}+\frac{\left( \sum w_{k}\hat{\beta}_{Yk}/\sigma_{Yk}^{2} \right)^{2}\left( \sum w_{k}^{2}/\sigma_{Yk}^{2} \right)}{\left( \sum w_{k}\hat{\beta}_{Xk}/\sigma_{Yk}^{2} \right)^{4}}}$$

Herein, the variance of $G_{k}$ is approximately proportional to $\sigma_{Yk}^{-2}$. When the equal weights are used, the $\hat{\beta}_{XY}$ and $\mathrm{se}\left( \hat{\beta}_{XY} \right)$ reduce to be the inverse-variance weighted estimates.

**References:**

1. Burgess S, Dudbridge F, Thompson SG. Combining information on multiple instrumental variables in Mendelian randomization: comparison of allele score and summarized data methods. *Stat Med* 2016;35(11):1880-906. doi: 10.1002/sim.6835 [published Online First: 2015/12/15]
