# Supplementary material for: Mendelian randomization study of interleukin (IL)-1 family and lung cancer
Source: Sci Rep. 2021 Sep 2;11:17606. doi: 10.1038/s41598-021-97099-5 (PMC8413403; doi:10.1038/s41598-021-97099-5)
Supplement: Supplementary file 3 — Supplementary Tables. [file 41598_2021_97099_MOESM3_ESM.docx]

**Supplementary Tables of “Mendelian randomization study of interleukin (IL)-1 family and lung cancer”**

Zhao Yang, C. Mary Schooling, Man Ki Kwok

Contents

**Table S1.** Identified protein quantitative trait locus for IL-1 family members/receptors in the two genome-wide association studies for proteomics 2

**Table S2.** Drugs approved Food and Drug Administration for treatment of lung cancer and the related targeted biomarkers 16

**Table S3.** *Cis*-pQTLs and associations with genetically predicted interleukin (IL)-1 family members/receptors with lung cancer and its subtypes 17

**Table S4.** Excluded pQTLs that were ill-annotated in RegulomeDB database in terms of allele frequency in European descendants 30

**Table S5.** Excluded *tran*-pQLTs that were associated with expression quantitative trait loci (eQTLs) in RegulomeDB database 31

**Table S6.** Excluded *trans*-pQTLs that were associated with expression quantitative trait loci (eQTLs) in PhenoScanner database 39

**Table S7.** Excluded *cis*-pQTLs that were associated with competing events related deaths 58

**Table S8.** Sensitivity analysis for effects of the genetically predicted high circulating interleukin (IL)-1 family members/receptors on lung cancer, lung adenocarcinoma, and squamous cell lung cancer 59

**Table S9.** Conditional F-statistic for testing instrument strength in the robust multivariable Mendelian randomization analysis via Models 1, 2, 3, and 4 67

**Table S10.** Robust multivariable Mendelian randomization estimates for the genetically predicted high circulating interleukin (IL)-1 family members/receptors on lung cancer, lung adenocarcinoma, and squamous cell lung cancer after removing interleukin-1 receptor antagonist (IL-1β), interleukin-36α (IL-36α), and interleukin-37 (IL-37) 68

**Table S11.** The IL-1Ra-rheumatoid arthritis associations using the inverse weighted methods based on the latest GWAS datasets

**Supplementary Tables**

# **Table S1.** Identified protein quantitative trait locus for IL-1 family members/receptors in the two genome-wide association studies for proteomics

| IL-1 family members / receptors | SNP | chr_hg19 | pos_hg19 | Effect allele | Other allele | beta | se | *P*-value | EAF | Gene | Type | Study |
| --- | --- | --- | --- | --- | --- | --- | --- | --- | --- | --- | --- | --- |
| IL-18 | rs385076 | 2 | 32489851 | C | T | 0.2432 | 0.0248 | 1.66E-22 | 0.64 | NLRC4 | trans-pQTL | Ahola-Olli et al. 2017 |
| IL-18 | rs1656939 | 3 | 186432744 | A | T | 0.1132 | 0.0236 | 1.56E-06 | 0.49 | KNG1 | cis-pQTL | Ahola-Olli et al. 2017 |
| IL-18 | rs4482818 | 4 | 65928497 | A | G | 0.1286 | 0.0244 | 1.45E-07 | 0.64 | RP11-707A18.1 | cis-pQTL | Ahola-Olli et al. 2017 |
| IL-18 | rs4414903 | 4 | 178470496 | G | C | 0.1161 | 0.025 | 3.31E-06 | 0.63 | RP11-130F10.1 | cis-pQTL | Ahola-Olli et al. 2017 |
| IL-18 | rs116383510 | 5 | 2545650 | C | A | 0.5426 | 0.1056 | 3.00E-07 | 0.02 | RP11-129I19.2 | cis-pQTL | Ahola-Olli et al. 2017 |
| IL-18 | rs116656892 | 5 | 68186028 | T | C | 0.5298 | 0.0925 | 1.05E-08 | 0.98 | CTC-340D7.1 | cis-pQTL | Ahola-Olli et al. 2017 |
| IL-18 | rs150005227 | 5 | 68224443 | T | C | 0.4349 | 0.0889 | 8.89E-07 | 0.01 | CTC-340D7.1 | cis-pQTL | Ahola-Olli et al. 2017 |
| IL-18 | rs115267715 | 5 | 68535015 | T | C | 0.4508 | 0.08 | 1.72E-08 | 0.02 | CDK7 | cis-pQTL | Ahola-Olli et al. 2017 |
| IL-18 | rs17229943 | 5 | 68682536 | C | A | 0.312 | 0.0463 | 1.62E-11 | 0.05 | RAD17 | trans-pQTL | Ahola-Olli et al. 2017 |
| IL-18 | rs658805 | 6 | 70909073 | A | G | 0.1226 | 0.0244 | 4.94E-07 | 0.32 | COL19A1 | cis-pQTL | Ahola-Olli et al. 2017 |
| IL-18 | rs117266781 | 7 | 41301020 | T | C | 0.6841 | 0.1468 | 3.15E-06 | 0.01 | AC005022.1 | cis-pQTL | Ahola-Olli et al. 2017 |
| IL-18 | rs1852105 | 7 | 63725595 | T | C | 0.3036 | 0.0661 | 4.32E-06 | 0.05 | ZNF679 | trans-pQTL | Ahola-Olli et al. 2017 |
| IL-18 | rs78623212 | 7 | 103307627 | T | C | 0.8705 | 0.1778 | 6.71E-07 | 0.03 | RELN | cis-pQTL | Ahola-Olli et al. 2017 |
| IL-18 | rs144841621 | 10 | 71681557 | T | C | 0.518 | 0.1141 | 3.81E-06 | 0.01 | COL13A1 | cis-pQTL | Ahola-Olli et al. 2017 |
| IL-18 | rs2729385 | 11 | 57262993 | A | G | 0.1231 | 0.0262 | 3.79E-06 | 0.33 | SLC43A1 | trans-pQTL | Ahola-Olli et al. 2017 |
| IL-18 | rs71478720 | 11 | 112009605 | C | T | 0.2669 | 0.0276 | 3.71E-22 | 0.78 | AP002884.2 | trans-pQTL | Ahola-Olli et al. 2017 |
| IL-18 | rs11214140 | 11 | 112086191 | C | T | 0.1531 | 0.0302 | 3.68E-07 | 0.18 | BCO2 | cis-pQTL | Ahola-Olli et al. 2017 |
| IL-18 | rs117903455 | 11 | 112271496 | G | A | 0.3626 | 0.0754 | 1.80E-06 | 0.97 | RP11-65M17.3 | cis-pQTL | Ahola-Olli et al. 2017 |
| IL-18 | rs1979967 | 15 | 79659613 | T | C | 0.1402 | 0.0286 | 9.45E-07 | 0.27 | TMED3 | trans-pQTL | Ahola-Olli et al. 2017 |
| IL-18 | rs143370787 | 16 | 74951514 | G | C | 0.3116 | 0.066 | 2.35E-06 | 0.08 | WDR59 | cis-pQTL | Ahola-Olli et al. 2017 |
| IL-18 | rs10414578 | 19 | 55146070 | C | T | 0.1771 | 0.035 | 4.16E-07 | 0.88 | LILRB1 | trans-pQTL | Ahola-Olli et al. 2017 |
| IL-18 | rs78716465 | 20 | 40643726 | A | G | 0.3265 | 0.0682 | 1.63E-06 | 0.04 | RP5-1121H13.3 | cis-pQTL | Ahola-Olli et al. 2017 |
| IL-18 | rs11700536 | 21 | 44558687 | T | C | 0.1156 | 0.025 | 4.21E-06 | 0.36 | AP001631.9 | trans-pQTL | Ahola-Olli et al. 2017 |
| IL-1β | rs115242021 | 1 | 145389965 | A | C | 0.2326 | 0.0414 | 5.07E-07 | 0.09 | U1 | trans-pQTL | Ahola-Olli et al. 2017 |
| IL-1β | rs143319329 | 7 | 128139459 | T | C | 0.2801 | 0.0715 | 2.00E-06 | 0.03 | METTL2B | cis-pQTL | Ahola-Olli et al. 2017 |
| IL-1β | rs61335305 | 15 | 66453074 | A | C | 0.2966 | 0.0724 | 1.90E-06 | 0.01 | MEGF11 | cis-pQTL | Ahola-Olli et al. 2017 |
| IL-1β | rs4786740 | 16 | 5675198 | A | C | 0.0845 | 0.0202 | 4.67E-06 | 0.41 | RP11-420N3.2 | cis-pQTL | Ahola-Olli et al. 2017 |
| IL-1β | rs62015704 | 16 | 7467907 | A | G | 0.1082 | 0.0283 | 2.09E-06 | 0.88 | RBFOX1 | cis-pQTL | Ahola-Olli et al. 2017 |
| IL-1β | rs9898641 | 17 | 57571033 | C | T | 0.2032 | 0.0454 | 3.59E-06 | 0.10 | RP11-567L7.6 | cis-pQTL | Ahola-Olli et al. 2017 |
| IL-1β | rs1942793 | 18 | 71104371 | T | G | 0.0717 | 0.0187 | 4.98E-06 | 0.47 | CTD-2354A18.1 | cis-pQTL | Ahola-Olli et al. 2017 |
| IL-1Ra | rs12121840 | 1 | 165541642 | T | C | 0.2692 | 0.0571 | 2.43E-06 | 0.08 | RP11-280O1.2 | trans-pQTL | Ahola-Olli et al. 2017 |
| IL-1Ra | rs56134659 | 3 | 129058985 | G | A | 0.1117 | 0.0237 | 2.44E-06 | 0.40 | H1FX-AS1 | cis-pQTL | Ahola-Olli et al. 2017 |
| IL-1Ra | rs1054402 | 9 | 119163509 | T | C | 0.1311 | 0.027 | 1.13E-06 | 0.26 | PAPPA | cis-pQTL | Ahola-Olli et al. 2017 |
| IL-1Ra | rs2809154 | 13 | 84727524 | C | T | 0.1786 | 0.0388 | 3.74E-06 | 0.82 | SLITRK1 | cis-pQTL | Ahola-Olli et al. 2017 |
| IL-1Ra | rs11627423 | 14 | 33200623 | A | C | 0.1171 | 0.0247 | 2.12E-06 | 0.61 | AKAP6 | cis-pQTL | Ahola-Olli et al. 2017 |
| IL-1Ra | rs61335305 | 15 | 66453074 | A | C | 0.4453 | 0.0908 | 1.00E-06 | 0.01 | MEGF11 | cis-pQTL | Ahola-Olli et al. 2017 |
| IL-1Ra | rs11869294 | 17 | 43857223 | G | C | 0.2274 | 0.0474 | 1.50E-06 | 0.03 | CRHR1 | cis-pQTL | Ahola-Olli et al. 2017 |
| IL-1Ra | rs147747784 | 19 | 55118193 | C | G | 0.3651 | 0.0757 | 1.46E-06 | 0.02 | LILRB1 | cis-pQTL | Ahola-Olli et al. 2017 |
| IL-1Ra | rs187166731 | 22 | 42666026 | C | T | 0.2325 | 0.0508 | 4.70E-06 | 0.98 | Z83851.3 | cis-pQTL | Ahola-Olli et al. 2017 |
| IL-1Ra | rs9623661 | 22 | 43093376 | C | T | 0.1966 | 0.0426 | 3.86E-06 | 0.91 | A4GALT | cis-pQTL | Ahola-Olli et al. 2017 |
| IL-18BP | rs200821392 | 10 | 5822184 | A | C | -0.1535 | 0.0318 | 1.35E-06 | 0.23 | GDI2 | cis-pQTL | Sun et al. 2018 |
| IL-18BP | rs11265492 | 1 | 160799274 | A | G | -0.14 | 0.0302 | 3.63E-06 | 0.21 | CD244 | trans-pQTL | Sun et al. 2018 |
| IL-18BP | rs6151432 | 15 | 59981647 | G | A | 0.232 | 0.0493 | 2.57E-06 | 0.08 | BNIP2 | cis-pQTL | Sun et al. 2018 |
| IL-18BP | rs16823361 | 2 | 144711507 | C | G | 0.2948 | 0.0576 | 3.09E-07 | 0.05 | AC016910.1 | cis-pQTL | Sun et al. 2018 |
| IL-18BP | rs11203320 | 1 | 17497738 | A | C | 0.1481 | 0.0306 | 1.32E-06 | 0.26 | Y_RNA | cis-pQTL | Sun et al. 2018 |
| IL-18BP | rs1600079 | 8 | 54359861 | T | C | -0.1337 | 0.0283 | 2.40E-06 | 0.74 | RP11-1081M5.1 | cis-pQTL | Sun et al. 2018 |
| IL-18BP | rs34396416 | 10 | 27856579 | T | C | -0.4187 | 0.0898 | 3.16E-06 | 0.02 | RAB18 | cis-pQTL | Sun et al. 2018 |
| IL-18BP | rs76726938 | 15 | 47767550 | T | G | 0.3713 | 0.0737 | 4.68E-07 | 0.03 | SEMA6D | cis-pQTL | Sun et al. 2018 |
| IL-18BP | rs146084600 | 14 | 43574439 | A | C | -0.5333 | 0.114 | 2.88E-06 | 0.01 | CTD-2307P3.1 | cis-pQTL | Sun et al. 2018 |
| IL-18BP | rs148844907 | 6 | 31628397 | A | T | -0.5208 | 0.114 | 4.90E-06 | 0.01 | C6orf47 | cis-pQTL | Sun et al. 2018 |
| IL-18BP | rs4806509 | 19 | 54337096 | T | G | 0.1463 | 0.0288 | 3.63E-07 | 0.67 | NLRP12 | cis-pQTL | Sun et al. 2018 |
| IL-18BP | rs62211692 | 21 | 21057339 | G | A | 0.146 | 0.0306 | 1.91E-06 | 0.23 | AP000946.2 | cis-pQTL | Sun et al. 2018 |
| IL-18BP | rs73402996 | 7 | 98426701 | A | G | -0.3306 | 0.0717 | 4.07E-06 | 0.03 | TMEM130 | trans-pQTL | Sun et al. 2018 |
| IL-18BP | rs12455835 | 18 | 49952739 | A | C | 0.3451 | 0.0743 | 3.47E-06 | 0.03 | DCC | cis-pQTL | Sun et al. 2018 |
| IL-18BP | rs6139149 | 20 | 3518137 | T | G | 0.2155 | 0.0445 | 1.32E-06 | 0.10 | ATRN | cis-pQTL | Sun et al. 2018 |
| IL-18Rα | rs61929078 | 12 | 94637905 | A | G | 0.6031 | 0.1319 | 4.79E-06 | 0.01 | PLXNC1 | cis-pQTL | Sun et al. 2018 |
| IL-18Rα | rs1801689 | 17 | 64210580 | C | A | 0.384 | 0.0713 | 7.24E-08 | 0.03 | APOH | cis-pQTL | Sun et al. 2018 |
| IL-18Rα | rs139519085 | 5 | 22461024 | G | T | 0.3389 | 0.0728 | 3.24E-06 | 0.04 | CDH12 | cis-pQTL | Sun et al. 2018 |
| IL-18Rα | rs9272100 | 6 | 32599814 | T | C | 0.1291 | 0.0281 | 4.47E-06 | 0.26 | HLA-DQA1 | cis-pQTL | Sun et al. 2018 |
| IL-18Rα | rs13014004 | 2 | 102840667 | T | C | -0.2623 | 0.0359 | 2.69E-13 | 0.14 | IL1RL2 | cis-pQTL | Sun et al. 2018 |
| IL-18Rα | rs13008334 | 2 | 103167031 | G | A | -0.224 | 0.0406 | 3.47E-08 | 0.11 | SLC9A4 | trans-pQTL | Sun et al. 2018 |
| IL-18Rα | rs141601545 | 2 | 102688642 | G | C | 0.4618 | 0.0695 | 3.09E-11 | 0.04 | IL1R1 | cis-pQTL | Sun et al. 2018 |
| IL-18Rα | rs58514453 | 10 | 112639605 | C | T | -0.3248 | 0.0705 | 3.98E-06 | 0.03 | PDCD4 | cis-pQTL | Sun et al. 2018 |
| IL-18Rα | rs58445015 | 7 | 98420178 | T | C | -0.3608 | 0.0717 | 4.90E-07 | 0.03 | TMEM130 | trans-pQTL | Sun et al. 2018 |
| IL-18Rα | rs34988270 | 5 | 31060384 | T | C | 0.2364 | 0.0507 | 3.09E-06 | 0.07 | RP11-152K4.2 | cis-pQTL | Sun et al. 2018 |
| IL-18Rα | rs2429175 | 12 | 2045085 | A | G | 0.1444 | 0.0303 | 1.86E-06 | 0.22 | RP5-1096D14.2 | cis-pQTL | Sun et al. 2018 |
| IL-18Rα | rs61929901 | 12 | 83513554 | G | C | 0.3968 | 0.0832 | 1.82E-06 | 0.03 | TMTC2 | cis-pQTL | Sun et al. 2018 |
| IL-18Rα | rs116932667 | 8 | 3450431 | T | A | 0.5713 | 0.1193 | 1.70E-06 | 0.01 | CSMD1 | cis-pQTL | Sun et al. 2018 |
| IL-18Rα | rs1420106 | 2 | 103035044 | G | A | -0.9085 | 0.0257 | 1.10E-273 | 0.78 | IL18RAP | trans-pQTL | Sun et al. 2018 |
| IL-18Rα | rs75968506 | 2 | 102809055 | A | T | 0.6676 | 0.0809 | 1.55E-16 | 0.03 | IL1RL2 | cis-pQTL | Sun et al. 2018 |
| IL-18Rα | rs181634856 | 2 | 102925950 | G | C | 0.9035 | 0.1121 | 7.76E-16 | 0.01 | IL1RL1 | cis-pQTL | Sun et al. 2018 |
| IL-18Rα | rs111686801 | 17 | 75047218 | A | G | 0.177 | 0.038 | 3.31E-06 | 0.13 | LINC00338 | cis-pQTL | Sun et al. 2018 |
| IL-18Rα | rs76972877 | 10 | 36725682 | G | C | 0.4252 | 0.089 | 1.78E-06 | 0.02 | RP11-92J19.3 | cis-pQTL | Sun et al. 2018 |
| IL-18Rα | rs149943125 | 2 | 102826146 | C | A | 0.6599 | 0.1287 | 2.95E-07 | 0.01 | IL1RL2 | cis-pQTL | Sun et al. 2018 |
| IL-18Rα | rs151254748 | 2 | 103107770 | T | C | 0.3534 | 0.0741 | 1.82E-06 | 0.04 | SLC9A4 | cis-pQTL | Sun et al. 2018 |
| IL-18Rα | rs6814269 | 4 | 117292394 | G | A | 0.1261 | 0.0259 | 1.12E-06 | 0.35 | RP11-659O3.1 | cis-pQTL | Sun et al. 2018 |
| IL-18Rα | rs10935033 | 3 | 132559591 | T | G | -0.1505 | 0.0328 | 4.47E-06 | 0.17 | NPHP3-AS1 | cis-pQTL | Sun et al. 2018 |
| IL-18Rα | rs12532129 | 7 | 129907555 | A | T | 0.1272 | 0.0268 | 2.09E-06 | 0.30 | CPA2 | cis-pQTL | Sun et al. 2018 |
| IL-18Rα | rs13014644 | 2 | 102971363 | T | G | -0.5629 | 0.0394 | 3.24E-46 | 0.10 | IL18R1 | trans-pQTL | Sun et al. 2018 |
| IL-18Rα | rs78123896 | 2 | 103075417 | C | T | -0.3866 | 0.0349 | 1.41E-28 | 0.14 | IL18RAP | trans-pQTL | Sun et al. 2018 |
| IL-18Rα | rs10167431 | 2 | 102852802 | C | T | -0.2075 | 0.0248 | 6.46E-17 | 0.55 | IL1RL2 | trans-pQTL | Sun et al. 2018 |
| IL-18Rα | rs11165484 | 1 | 96241684 | T | C | -0.14 | 0.0283 | 7.76E-07 | 0.26 | RP11-286B14.1 | cis-pQTL | Sun et al. 2018 |
| IL-18Rα | rs4140836 | 2 | 103268511 | G | A | -0.248 | 0.0332 | 7.76E-14 | 0.81 | SLC9A2 | trans-pQTL | Sun et al. 2018 |
| IL-18Rα | rs190750383 | 5 | 20849558 | G | A | -0.3895 | 0.0744 | 1.66E-07 | 0.04 | RP11-774D14.1 | cis-pQTL | Sun et al. 2018 |
| IL-18Rα | rs79171645 | 10 | 24971221 | T | G | -0.2894 | 0.0588 | 8.71E-07 | 0.05 | ARHGAP21 | cis-pQTL | Sun et al. 2018 |
| IL-18Rα | rs17833274 | 2 | 103319666 | T | C | 0.3646 | 0.0609 | 2.19E-09 | 0.06 | SLC9A2 | cis-pQTL | Sun et al. 2018 |
| IL-18Rα | rs114003263 | 2 | 103002194 | A | G | 0.7564 | 0.1064 | 1.20E-12 | 0.02 | IL18R1 | cis-pQTL | Sun et al. 2018 |
| IL-18Rα | rs75778877 | 2 | 103174669 | C | T | 0.6355 | 0.1369 | 3.47E-06 | 0.01 | SLC9A4 | cis-pQTL | Sun et al. 2018 |
| IL-18Rα | rs4801396 | 19 | 57406829 | A | T | -0.1178 | 0.0255 | 3.72E-06 | 0.62 | ZIM2 | cis-pQTL | Sun et al. 2018 |
| IL-1α | rs13170249 | 5 | 102337155 | T | C | 0.3527 | 0.0768 | 4.37E-06 | 0.03 | PAM | cis-pQTL | Sun et al. 2018 |
| IL-1α | rs78084692 | 7 | 26444836 | G | A | 0.8742 | 0.1817 | 1.51E-06 | 0.01 | AC004540.5 | cis-pQTL | Sun et al. 2018 |
| IL-1α | rs7148359 | 14 | 28296716 | T | C | -0.1354 | 0.0286 | 2.14E-06 | 0.27 | AL445384.1 | cis-pQTL | Sun et al. 2018 |
| IL-1α | rs4867603 | 5 | 169884465 | C | T | -0.1305 | 0.0267 | 1.05E-06 | 0.55 | KCNIP1 | cis-pQTL | Sun et al. 2018 |
| IL-1α | rs10418046 | 19 | 54327869 | G | T | 0.1529 | 0.0299 | 3.09E-07 | 0.22 | NLRP12 | cis-pQTL | Sun et al. 2018 |
| IL-1α | rs6549977 | 3 | 30259240 | A | G | -0.1196 | 0.0259 | 3.89E-06 | 0.38 | AC137674.2 | cis-pQTL | Sun et al. 2018 |
| IL-1α | rs8107140 | 19 | 57410200 | A | C | 0.1147 | 0.0248 | 3.63E-06 | 0.59 | ZIM2 | cis-pQTL | Sun et al. 2018 |
| IL-1α | rs28895307 | 15 | 31888842 | G | T | 0.3649 | 0.0721 | 4.27E-07 | 0.03 | OTUD7A | cis-pQTL | Sun et al. 2018 |
| IL-1α | rs60010354 | 11 | 108513088 | G | A | 0.1142 | 0.0248 | 4.17E-06 | 0.42 | DDX10 | cis-pQTL | Sun et al. 2018 |
| IL-1α | rs41386050 | 18 | 47095675 | T | G | 0.3175 | 0.0694 | 4.79E-06 | 0.03 | LIPG | cis-pQTL | Sun et al. 2018 |
| IL-1α | rs2734376 | 3 | 60417339 | T | C | 0.1397 | 0.0278 | 4.90E-07 | 0.69 | FHIT | cis-pQTL | Sun et al. 2018 |
| IL-1α | rs2749675 | 6 | 157795506 | G | A | -0.1716 | 0.0374 | 4.37E-06 | 0.87 | ZDHHC14 | trans-pQTL | Sun et al. 2018 |
| IL-1α | rs77869959 | 13 | 110353686 | A | C | -0.2402 | 0.0523 | 4.47E-06 | 0.06 | RP11-313L9.1 | cis-pQTL | Sun et al. 2018 |
| IL-1α | rs6033085 | 20 | 11258339 | T | A | -0.1796 | 0.0393 | 4.90E-06 | 0.89 | RP4-734C18.1 | cis-pQTL | Sun et al. 2018 |
| IL-1α | rs72647025 | 1 | 20605845 | G | C | 0.1338 | 0.0281 | 1.86E-06 | 0.39 | VWA5B1 | cis-pQTL | Sun et al. 2018 |
| IL-1α | rs11922046 | 3 | 137247176 | T | C | 0.1392 | 0.0287 | 1.20E-06 | 0.26 | RN5S142 | cis-pQTL | Sun et al. 2018 |
| IL-1α | rs139711468 | 12 | 20091985 | A | G | -0.347 | 0.0728 | 1.86E-06 | 0.03 | RP11-405A12.2 | cis-pQTL | Sun et al. 2018 |
| IL-1α | rs4757027 | 11 | 20085107 | C | G | -0.1425 | 0.0279 | 3.39E-07 | 0.68 | NAV2 | cis-pQTL | Sun et al. 2018 |
| IL-1α | rs192996211 | 10 | 8834680 | A | G | -0.4686 | 0.1026 | 4.90E-06 | 0.02 | RP11-428L9.1 | cis-pQTL | Sun et al. 2018 |
| IL-1R1 | rs1652049 | 14 | 21050409 | G | T | 0.1284 | 0.0268 | 1.62E-06 | 0.46 | RNASE11 | cis-pQTL | Sun et al. 2018 |
| IL-1R1 | rs71358078 | 18 | 4960876 | T | C | -0.4061 | 0.0852 | 1.86E-06 | 0.02 | RP11-172F10.1 | cis-pQTL | Sun et al. 2018 |
| IL-1R1 | rs112081515 | 18 | 46617494 | G | A | -0.2861 | 0.0617 | 3.55E-06 | 0.04 | DYM | cis-pQTL | Sun et al. 2018 |
| IL-1R1 | rs10208542 | 2 | 102732315 | C | G | -0.1852 | 0.0372 | 6.46E-07 | 0.13 | IL1R1 | cis-pQTL | Sun et al. 2018 |
| IL-1R1 | rs77451959 | 11 | 30383235 | T | G | 0.3996 | 0.0855 | 2.95E-06 | 0.02 | MPPED2 | cis-pQTL | Sun et al. 2018 |
| IL-1R1 | rs12493830 | 3 | 98406794 | C | T | -0.1378 | 0.0254 | 6.17E-08 | 0.45 | ST3GAL6-AS1 | trans-pQTL | Sun et al. 2018 |
| IL-1R1 | rs76053345 | 9 | 112078767 | C | T | -0.7564 | 0.1567 | 1.38E-06 | 0.01 | EPB41L4B | cis-pQTL | Sun et al. 2018 |
| IL-1R1 | rs140225543 | 19 | 55734200 | A | G | -0.5534 | 0.1192 | 3.47E-06 | 0.01 | TMEM86B | cis-pQTL | Sun et al. 2018 |
| IL-1R1 | rs72771293 | 5 | 82948816 | G | A | 0.1504 | 0.031 | 1.17E-06 | 0.22 | HAPLN1 | cis-pQTL | Sun et al. 2018 |
| IL-1R1 | rs78801677 | 10 | 68721659 | A | G | 0.206 | 0.0443 | 3.31E-06 | 0.09 | LRRTM3 | cis-pQTL | Sun et al. 2018 |
| IL-1R1 | rs144792666 | 4 | 44581976 | T | C | 0.4847 | 0.1006 | 1.45E-06 | 0.02 | YIPF7 | cis-pQTL | Sun et al. 2018 |
| IL-1R1 | rs35583440 | 11 | 32075291 | C | G | 0.8739 | 0.191 | 4.79E-06 | 0.01 | RCN1 | cis-pQTL | Sun et al. 2018 |
| IL-1R1 | rs11888059 | 2 | 102665086 | G | A | 0.1153 | 0.0247 | 3.02E-06 | 0.50 | IL1R1 | trans-pQTL | Sun et al. 2018 |
| IL-1R1 | rs145533598 | 3 | 12373486 | A | G | 0.4698 | 0.0965 | 1.12E-06 | 0.02 | PPARG | cis-pQTL | Sun et al. 2018 |
| IL-1R1 | rs10424405 | 19 | 54321933 | G | A | -0.1463 | 0.0302 | 1.32E-06 | 0.22 | NLRP12 | cis-pQTL | Sun et al. 2018 |
| IL-1R1 | rs55923630 | 3 | 118806101 | T | A | -0.1494 | 0.0263 | 1.35E-08 | 0.30 | IGSF11 | cis-pQTL | Sun et al. 2018 |
| IL-1R1 | rs150889493 | 16 | 6804488 | C | T | -0.4382 | 0.0888 | 7.94E-07 | 0.02 | RP11-420N3.2 | cis-pQTL | Sun et al. 2018 |
| IL-1R1 | rs4690014 | 4 | 3436062 | A | G | 0.1325 | 0.0258 | 2.69E-07 | 0.39 | RGS12 | trans-pQTL | Sun et al. 2018 |
| IL-1R1 | rs143365592 | 21 | 31766693 | G | T | 0.5682 | 0.1242 | 4.79E-06 | 0.01 | KRTAP13-1 | cis-pQTL | Sun et al. 2018 |
| IL-1R1 | rs10972130 | 9 | 34469858 | A | G | 0.1465 | 0.031 | 2.29E-06 | 0.20 | DNAI1 | cis-pQTL | Sun et al. 2018 |
| IL-1Racp | rs1988743 | 3 | 190267900 | C | G | 0.1353 | 0.0281 | 1.51E-06 | 0.72 | IL1RAP | trans-pQTL | Sun et al. 2018 |
| IL-1Racp | rs2885370 | 3 | 190276995 | T | G | -0.2086 | 0.0306 | 8.91E-12 | 0.74 | IL1RAP | cis-pQTL | Sun et al. 2018 |
| IL-1Racp | rs41268633 | 3 | 190327020 | G | A | -0.2514 | 0.0504 | 6.03E-07 | 0.09 | IL1RAP | cis-pQTL | Sun et al. 2018 |
| IL-1Racp | rs113553332 | 3 | 190521288 | T | C | 0.6851 | 0.0563 | 4.68E-34 | 0.05 | GMNC | cis-pQTL | Sun et al. 2018 |
| IL-1Racp | rs148996090 | 3 | 190347247 | A | C | 1.1107 | 0.1365 | 3.98E-16 | 0.01 | IL1RAP | cis-pQTL | Sun et al. 2018 |
| IL-1Racp | rs67249092 | 3 | 190357571 | G | A | -0.379 | 0.0345 | 5.25E-28 | 0.15 | IL1RAP | cis-pQTL | Sun et al. 2018 |
| IL-1Racp | rs6444435 | 3 | 190303805 | G | A | -0.1705 | 0.026 | 5.37E-11 | 0.35 | IL1RAP | trans-pQTL | Sun et al. 2018 |
| IL-1Racp | rs1024949 | 3 | 190347732 | C | T | -0.4479 | 0.0399 | 2.95E-29 | 0.13 | IL1RAP | trans-pQTL | Sun et al. 2018 |
| IL-1Racp | rs3935774 | 3 | 190360571 | T | C | 0.7411 | 0.1119 | 3.55E-11 | 0.02 | IL1RAP | cis-pQTL | Sun et al. 2018 |
| IL-1Racp | rs13084868 | 3 | 190515941 | C | T | 0.1782 | 0.0388 | 4.27E-06 | 0.12 | GMNC | trans-pQTL | Sun et al. 2018 |
| IL-1Racp | rs1607264 | 2 | 180367104 | C | A | -0.1455 | 0.0311 | 2.82E-06 | 0.80 | ZNF385B | cis-pQTL | Sun et al. 2018 |
| IL-1Racp | rs62196769 | 2 | 195921640 | C | A | -0.2046 | 0.0439 | 3.09E-06 | 0.09 | AC010983.1 | cis-pQTL | Sun et al. 2018 |
| IL-1Racp | rs4815878 | 20 | 647736 | A | G | 0.1551 | 0.0298 | 1.95E-07 | 0.76 | SRXN1 | trans-pQTL | Sun et al. 2018 |
| IL-1Racp | rs6461953 | 7 | 3292985 | C | T | -0.1459 | 0.0319 | 4.68E-06 | 0.18 | AC073316.1 | cis-pQTL | Sun et al. 2018 |
| IL-1Racp | rs4687154 | 3 | 190304172 | C | G | -0.581 | 0.0396 | 7.94E-49 | 0.89 | IL1RAP | cis-pQTL | Sun et al. 2018 |
| IL-1Racp | rs6796131 | 3 | 190312132 | C | G | -0.2436 | 0.0262 | 1.51E-20 | 0.33 | IL1RAP | cis-pQTL | Sun et al. 2018 |
| IL-1Racp | rs4624606 | 3 | 190354246 | T | A | -0.9248 | 0.0248 | 9.12E-304 | 0.75 | IL1RAP | cis-pQTL | Sun et al. 2018 |
| IL-1Racp | rs6444430 | 3 | 190223191 | A | G | 0.1543 | 0.0326 | 2.29E-06 | 0.79 | IL1RAP | cis-pQTL | Sun et al. 2018 |
| IL-1Racp | rs16969649 | 18 | 35657401 | T | C | -0.14 | 0.0306 | 4.90E-06 | 0.21 | MIR4318 | cis-pQTL | Sun et al. 2018 |
| IL-1Racp | rs7623802 | 3 | 81009014 | T | C | -0.129 | 0.0267 | 1.41E-06 | 0.66 | RP11-6B4.1 | cis-pQTL | Sun et al. 2018 |
| IL-1Racp | rs62116908 | 2 | 4478023 | C | T | -0.2937 | 0.0635 | 3.72E-06 | 0.04 | AC022311.1 | cis-pQTL | Sun et al. 2018 |
| IL-1Racp | rs61492644 | 6 | 13387898 | A | G | -0.124 | 0.0251 | 7.41E-07 | 0.52 | GFOD1 | cis-pQTL | Sun et al. 2018 |
| IL-1Racp | rs6801017 | 3 | 190295730 | G | A | -0.2023 | 0.0306 | 3.80E-11 | 0.21 | IL1RAP | cis-pQTL | Sun et al. 2018 |
| IL-1Racp | rs78129851 | 3 | 190374933 | A | G | 0.7929 | 0.1167 | 1.07E-11 | 0.01 | IL1RAP | cis-pQTL | Sun et al. 2018 |
| IL-1Racp | rs5753690 | 22 | 31950005 | T | C | 0.1493 | 0.0291 | 2.95E-07 | 0.23 | SFI1 | trans-pQTL | Sun et al. 2018 |
| IL-1Racp | rs13059920 | 3 | 190357293 | G | A | 0.4199 | 0.0377 | 9.33E-29 | 0.13 | IL1RAP | trans-pQTL | Sun et al. 2018 |
| IL-1Racp | rs73754360 | 6 | 93437809 | G | T | 0.3836 | 0.0803 | 1.78E-06 | 0.03 | RP11-127B16.1 | cis-pQTL | Sun et al. 2018 |
| IL-1Racp | rs75857200 | 14 | 28735179 | A | C | 0.1985 | 0.042 | 2.34E-06 | 0.10 | CTD-2591A6.2 | cis-pQTL | Sun et al. 2018 |
| IL-36α | rs2072162 | 7 | 105636458 | A | G | -0.1193 | 0.0259 | 4.07E-06 | 0.39 | CDHR3 | cis-pQTL | Sun et al. 2018 |
| IL-36α | rs142498034 | 2 | 5561902 | T | C | -0.53 | 0.1104 | 1.58E-06 | 0.02 | AC107057.1 | cis-pQTL | Sun et al. 2018 |
| IL-36α | rs9273410 | 6 | 32627250 | A | C | 0.1247 | 0.0253 | 8.51E-07 | 0.56 | HLA-DQB1 | cis-pQTL | Sun et al. 2018 |
| IL-36α | rs62517456 | 8 | 134697396 | G | A | 0.219 | 0.0457 | 1.66E-06 | 0.08 | SNORA40 | cis-pQTL | Sun et al. 2018 |
| IL-36α | rs704 | 17 | 26694861 | A | G | 0.3353 | 0.024 | 2.24E-44 | 0.47 | SEBOX | trans-pQTL | Sun et al. 2018 |
| IL-36α | rs4239214 | 17 | 26327892 | A | G | 0.1968 | 0.0413 | 1.95E-06 | 0.10 | SCARNA20 | trans-pQTL | Sun et al. 2018 |
| IL-36α | rs62065286 | 17 | 26748521 | G | A | -0.1307 | 0.0257 | 3.80E-07 | 0.64 | SLC46A1 | trans-pQTL | Sun et al. 2018 |
| IL-36α | rs74480769 | 5 | 40972211 | G | A | -0.494 | 0.0733 | 1.55E-11 | 0.03 | C7 | trans-pQTL | Sun et al. 2018 |
| IL-36α | rs10922094 | 1 | 196661505 | C | G | -0.1468 | 0.0254 | 7.24E-09 | 0.61 | CFH | trans-pQTL | Sun et al. 2018 |
| IL-36α | rs141026778 | 4 | 5412936 | A | G | -0.2671 | 0.0579 | 3.98E-06 | 0.05 | STK32B | cis-pQTL | Sun et al. 2018 |
| IL-36α | rs6767738 | 3 | 96785335 | G | A | -0.1287 | 0.0277 | 3.55E-06 | 0.28 | EPHA6 | cis-pQTL | Sun et al. 2018 |
| IL-36α | rs749331 | 2 | 23882347 | A | G | -0.1147 | 0.0246 | 3.09E-06 | 0.45 | KLHL29 | trans-pQTL | Sun et al. 2018 |
| IL-36α | rs2280484 | 9 | 138904840 | G | C | 0.2013 | 0.0426 | 2.29E-06 | 0.10 | NACC2 | cis-pQTL | Sun et al. 2018 |
| IL-36α | rs35995719 | 17 | 26612545 | A | G | -0.2934 | 0.0537 | 4.57E-08 | 0.06 | AC061975.8 | cis-pQTL | Sun et al. 2018 |
| IL-36α | rs10756815 | 9 | 16789081 | A | G | 0.1788 | 0.035 | 3.24E-07 | 0.85 | BNC2 | cis-pQTL | Sun et al. 2018 |
| IL-36α | rs72785161 | 16 | 68751154 | T | C | -0.2233 | 0.0483 | 3.89E-06 | 0.07 | CDH3 | cis-pQTL | Sun et al. 2018 |
| IL-36α | rs12194933 | 6 | 159641706 | A | G | 0.1755 | 0.0366 | 1.66E-06 | 0.14 | FNDC1 | trans-pQTL | Sun et al. 2018 |
| IL-36β | rs78917327 | 2 | 124361393 | A | G | 0.5562 | 0.1151 | 1.35E-06 | 0.01 | AC092646.3 | cis-pQTL | Sun et al. 2018 |
| IL-36β | rs1905676 | 12 | 30722838 | T | C | -0.1265 | 0.0274 | 4.07E-06 | 0.38 | IPO8 | cis-pQTL | Sun et al. 2018 |
| IL-36β | rs76528994 | 3 | 194230925 | G | A | 0.3323 | 0.0665 | 5.89E-07 | 0.04 | AC046143.7 | cis-pQTL | Sun et al. 2018 |
| IL-36β | rs199789755 | 5 | 128647865 | G | A | -0.1958 | 0.0426 | 4.27E-06 | 0.10 | MIR4460 | cis-pQTL | Sun et al. 2018 |
| IL-36β | rs62143194 | 19 | 54319624 | G | C | -0.1471 | 0.0306 | 1.58E-06 | 0.22 | NLRP12 | cis-pQTL | Sun et al. 2018 |
| IL-36β | rs1026519 | 18 | 74801678 | A | G | -0.3426 | 0.0694 | 7.94E-07 | 0.03 | MBP | trans-pQTL | Sun et al. 2018 |
| IL-36β | rs139281982 | 4 | 76184203 | T | C | -0.4304 | 0.0931 | 3.80E-06 | 0.02 | RP11-542G1.1 | cis-pQTL | Sun et al. 2018 |
| IL-36β | rs71430365 | 2 | 242530419 | G | A | -0.1467 | 0.0318 | 4.07E-06 | 0.22 | THAP4 | cis-pQTL | Sun et al. 2018 |
| IL-36β | rs74882099 | 11 | 69865438 | A | G | 0.3168 | 0.0659 | 1.51E-06 | 0.04 | RP11-626H12.2 | cis-pQTL | Sun et al. 2018 |
| IL-36β | rs16880182 | 8 | 32820097 | C | T | -0.269 | 0.0565 | 1.95E-06 | 0.05 | RP11-11N9.4 | cis-pQTL | Sun et al. 2018 |
| IL-36β | rs188013949 | 2 | 10591995 | A | G | 0.5876 | 0.1254 | 2.75E-06 | 0.01 | AC007249.3 | cis-pQTL | Sun et al. 2018 |
| IL-36β | rs149622390 | 3 | 105085939 | C | G | 0.4816 | 0.1033 | 3.09E-06 | 0.02 | ALCAM | cis-pQTL | Sun et al. 2018 |
| IL-36β | rs9345080 | 6 | 91758073 | T | A | -0.4483 | 0.0958 | 2.88E-06 | 0.02 | RP1-177I10.1 | cis-pQTL | Sun et al. 2018 |
| IL-36β | rs11002612 | 10 | 80380967 | C | T | -0.2157 | 0.0466 | 3.72E-06 | 0.08 | RP11-90J7.3 | cis-pQTL | Sun et al. 2018 |
| IL-36β | rs184204067 | 4 | 41493801 | T | C | 0.4742 | 0.0952 | 6.31E-07 | 0.02 | LIMCH1 | cis-pQTL | Sun et al. 2018 |
| IL-36β | rs11042951 | 11 | 10773477 | G | A | -0.449 | 0.098 | 4.68E-06 | 0.02 | CTR9 | cis-pQTL | Sun et al. 2018 |
| IL-36β | rs56208304 | 4 | 8184543 | C | T | 0.1366 | 0.028 | 1.07E-06 | 0.32 | SH3TC1 | trans-pQTL | Sun et al. 2018 |
| IL-36β | rs12191382 | 6 | 67403169 | C | T | 0.1309 | 0.0263 | 6.61E-07 | 0.32 | RNU7-66P | cis-pQTL | Sun et al. 2018 |
| IL-36β | rs170187 | 5 | 162062313 | C | T | -0.1327 | 0.029 | 4.90E-06 | 0.75 | RP11-167P20.1 | cis-pQTL | Sun et al. 2018 |
| IL-36γ | rs6704506 | 1 | 61131599 | C | T | -0.1321 | 0.0251 | 1.45E-07 | 0.60 | RP11-436K8.1 | cis-pQTL | Sun et al. 2018 |
| IL-36γ | rs548416824 |  | 5979820 | C | A | -0.3782 | 0.0772 | 9.55E-07 | 0.04 |  | cis-pQTL | Sun et al. 2018 |
| IL-36γ | rs1421799 | 5 | 97437976 | C | A | 0.1645 | 0.0354 | 3.39E-06 | 0.14 | AC008834.1 | cis-pQTL | Sun et al. 2018 |
| IL-36γ | rs17625605 | 1 | 177628819 | A | G | -0.2852 | 0.0624 | 4.90E-06 | 0.04 | RP11-63B19.1 | cis-pQTL | Sun et al. 2018 |
| IL-36γ | rs13075826 | 3 | 134464916 | T | C | 0.34 | 0.0637 | 9.55E-08 | 0.04 | EPHB1 | cis-pQTL | Sun et al. 2018 |
| IL-36γ | rs188628536 | 7 | 94595961 | T | A | -0.3981 | 0.0769 | 2.24E-07 | 0.03 | PPP1R9A | cis-pQTL | Sun et al. 2018 |
| IL-36γ | rs75882899 | 3 | 178821741 | G | A | -0.5101 | 0.1115 | 4.79E-06 | 0.01 | RP11-360P21.2 | cis-pQTL | Sun et al. 2018 |
| IL-36γ | rs2761713 | 9 | 9757738 | T | A | 0.1314 | 0.0284 | 3.72E-06 | 0.75 | PTPRD | cis-pQTL | Sun et al. 2018 |
| IL-36γ | rs117038998 | 10 | 133584751 | T | G | 0.2608 | 0.0571 | 4.90E-06 | 0.05 | AL450307.1 | cis-pQTL | Sun et al. 2018 |
| IL-36γ | rs13340573 | 8 | 134680936 | G | A | 0.2238 | 0.0471 | 2.00E-06 | 0.08 | SNORA40 | cis-pQTL | Sun et al. 2018 |
| IL-36γ | rs41315858 | 1 | 237778082 | A | G | -0.3686 | 0.0687 | 8.13E-08 | 0.03 | RYR2 | cis-pQTL | Sun et al. 2018 |
| IL-37 | rs2921184 | 5 | 41247288 | G | A | -0.1781 | 0.0371 | 1.55E-06 | 0.87 | C6 | cis-pQTL | Sun et al. 2018 |
| IL-37 | rs1955055 | 8 | 123305884 | G | A | -0.1495 | 0.0323 | 3.63E-06 | 0.20 | RP11-94A24.1 | cis-pQTL | Sun et al. 2018 |
| IL-37 | rs61191477 | 18 | 76148662 | A | C | 0.2289 | 0.049 | 2.95E-06 | 0.07 | RP11-451L19.1 | cis-pQTL | Sun et al. 2018 |
| IL-37 | rs2332686 | 17 | 54712453 | A | C | 0.1341 | 0.0288 | 3.31E-06 | 0.29 | NOG | cis-pQTL | Sun et al. 2018 |
| IL-37 | rs17819726 | 10 | 107883904 | G | A | 0.3606 | 0.0702 | 2.82E-07 | 0.04 | RP11-298H24.1 | cis-pQTL | Sun et al. 2018 |
| IL-37 | rs117079751 | 12 | 1253080 | G | A | -0.4297 | 0.0919 | 2.95E-06 | 0.02 | ERC1 | cis-pQTL | Sun et al. 2018 |
| IL-37 | rs74480769 | 5 | 40972211 | G | A | -0.5156 | 0.0732 | 1.91E-12 | 0.03 | C7 | trans-pQTL | Sun et al. 2018 |
| IL-37 | rs180881121 | 5 | 40818975 | A | G | -0.5753 | 0.1225 | 2.63E-06 | 0.01 | RPL37 | cis-pQTL | Sun et al. 2018 |
| IL-37 | rs76027472 | 11 | 96609344 | T | C | -0.233 | 0.0475 | 9.12E-07 | 0.08 | RP11-360K13.1 | cis-pQTL | Sun et al. 2018 |
| IL-37 | rs4595126 | 8 | 12687202 | A | T | 0.2216 | 0.0436 | 3.72E-07 | 0.09 | KIAA1456 | cis-pQTL | Sun et al. 2018 |
| IL-37 | rs956142 | 16 | 51091119 | T | C | -0.1214 | 0.0255 | 1.91E-06 | 0.42 | RP11-883G14.4 | cis-pQTL | Sun et al. 2018 |
| IL-37 | rs11259827 | 10 | 47601327 | G | A | 0.2135 | 0.0455 | 2.69E-06 | 0.11 | RP11-292F22.6 | trans-pQTL | Sun et al. 2018 |
| IL-37 | rs28661771 | 12 | 133000203 | C | T | -0.1633 | 0.034 | 1.55E-06 | 0.84 | RP11-503G7.1 | cis-pQTL | Sun et al. 2018 |
| IL-37 | rs6981787 | 8 | 8785880 | G | C | 0.173 | 0.0377 | 4.47E-06 | 0.12 | U6 | trans-pQTL | Sun et al. 2018 |
| IL-37 | rs737952 | 22 | 30978073 | T | C | -0.2743 | 0.0582 | 2.51E-06 | 0.05 | PES1 | cis-pQTL | Sun et al. 2018 |
| IL-37 | rs7539005 | 1 | 196667252 | T | A | -0.1696 | 0.0254 | 2.57E-11 | 0.61 | CFH | trans-pQTL | Sun et al. 2018 |
| IL-37 | rs62410788 | 4 | 14884053 | T | C | -0.1941 | 0.0419 | 3.55E-06 | 0.10 | LINC00504 | cis-pQTL | Sun et al. 2018 |

# **Table S2.** Drugs approved Food and Drug Administration for treatment of lung cancer and the related targeted biomarkers

| Target biomarkers | Drugs | Conditions | Clinical trials information |
| --- | --- | --- | --- |
| Serine/threonine-protein kinase | Dabrafemib mesylate | Patients with metastasized non-small cell lung cancer and a certain mutation in the BRAF gene | https://www.cancer.gov/about-cancer/treatment/clinical-trials/intervention/dabrafenib-mesylate?redirect=true |
| Mitogen-activated protein kinase/MAPK | Mechlorethamine hydrochloride | Patients with bronchogenic carcinoma | https://www.cancer.gov/publications/dictionaries/cancer-drug/def/mechlorethamine-hydrochloride?redirect=true |
| Programmed cell death protein | Atezolizumab | Patients with metastasized non-small cell lung cancer or small cell lung cancer | https://www.cancer.gov/about-cancer/treatment/clinical-trials/intervention/atezolizumab?redirect=true |
|  | Durvalumab | Patients with stage III non-small cell lung cancer that cannot be removed by surgery and did not get worse after platinum chemotherapy and radiation therapy | https://www.cancer.gov/about-cancer/treatment/clinical-trials/intervention/durvalumab?redirect=true |
|  | Pembrolizamab | Patients with metastasized non-small cell lung cancer or small cell lung cancer | https://www.cancer.gov/about-cancer/treatment/clinical-trials/intervention/pembrolizumab?redirect=true |
|  | Nivolumab | Patients with metastasized non-small cell lung cancer or small cell lung cancer | https://www.cancer.gov/about-cancer/treatment/clinical-trials/intervention/nivolumab?redirect=true |
| Tyrosine-protein kinase or recetor tyrosine kinase | Alunbrig | Patients with metastasized non-small lung cancer that is ALK positive | https://www.cancer.gov/about-cancer/treatment/clinical-trials/intervention/dual-alkegfr-inhibitor-ap26113?redirect=true |
|  | Certinib | Patients with metastasized non-small lung cancer that is ALK positive | https://www.cancer.gov/about-cancer/treatment/clinical-trials/intervention/ceritinib?redirect=true |
|  | Crizotinib | Patients with metastasized non-small lung cancer that is ALK positive or ROS1 positive | https://www.cancer.gov/about-cancer/treatment/clinical-trials/intervention/crizotinib?redirect=true |
|  | Alectinib | Patients with metastasized non-small lung cancer that is ALK positive | https://www.cancer.gov/about-cancer/treatment/clinical-trials/intervention/alectinib?redirect=true |
| Tubulin | Vinorelbine tartrate | Patients with non-small lung cancer | https://www.cancer.gov/about-cancer/treatment/clinical-trials/intervention/vinorelbine-tartrate?redirect=true |

# **Table S3.** *Cis*-pQTLs and associations with genetically predicted interleukin (IL)-1 family members/receptors with lung cancer and its subtypes

| Exposures | Outcomes | *cis*-pQTL | Effect allele | Other allele | EAF (exposure) | Beta (exposure) | Se (exposure) | *P-*value (exposure) | EAF (outcome) | Beta (outcome) | Se (outcome) | *P-*value (outcome) | F-statistics |
| --- | --- | --- | --- | --- | --- | --- | --- | --- | --- | --- | --- | --- | --- |
| IL-18 | Lung cancer | rs11214140 | C | T | 0.18 | 0.1531 | 0.0302 | 3.68E-07 | 0.17 | -0.0299 | 0.0269 | 2.94E-01 | 25.70 |
| IL-18 | Lung cancer | rs116383510 | C | A | 0.02 | 0.5426 | 0.1056 | 3.00E-07 | 0.01 | 0.0772 | 0.0869 | 3.78E-01 | 26.40 |
| IL-18 | Lung cancer | rs117266781 | T | C | 0.01 | 0.6841 | 0.1468 | 3.15E-06 | 0.01 | 0.1902 | 0.0952 | 2.61E-02 | 21.72 |
| IL-18 | Lung cancer | rs144841621 | T | C | 0.01 | 0.5180 | 0.1141 | 3.81E-06 | 0.01 | -0.1344 | 0.0966 | 2.81E-01 | 20.61 |
| IL-18 | Lung cancer | rs150005227 | T | C | 0.01 | 0.4349 | 0.0889 | 8.89E-07 | 0.01 | 0.2240 | 0.1335 | 6.13E-02 | 23.93 |
| IL-18 | Lung cancer | rs4482818 | A | G | 0.64 | 0.1286 | 0.0244 | 1.45E-07 | 0.63 | -0.0097 | 0.0203 | 6.36E-01 | 27.78 |
| IL-18 | Lung cancer | rs658805 | A | G | 0.32 | 0.1226 | 0.0244 | 4.94E-07 | 0.31 | -0.0163 | 0.0189 | 4.06E-01 | 25.25 |
| IL-18 | Lung cancer | rs78716465 | A | G | 0.04 | 0.3265 | 0.0682 | 1.63E-06 | 0.04 | 0.0457 | 0.0526 | 3.88E-01 | 22.92 |
| IL-18 | Lung adenocarcinoma | rs11214140 | C | T | 0.18 | 0.1531 | 0.0302 | 3.68E-07 | 0.17 | -0.0317 | 0.0408 | 4.70E-01 | 25.70 |
| IL-18 | Lung adenocarcinoma | rs116383510 | C | A | 0.02 | 0.5426 | 0.1056 | 3.00E-07 | 0.01 | 0.1223 | 0.1349 | 3.68E-01 | 26.40 |
| IL-18 | Lung adenocarcinoma | rs117266781 | T | C | 0.01 | 0.6841 | 0.1468 | 3.15E-06 | 0.01 | 0.1362 | 0.1357 | 3.12E-01 | 21.72 |
| IL-18 | Lung adenocarcinoma | rs144841621 | T | C | 0.01 | 0.5180 | 0.1141 | 3.81E-06 | 0.01 | -0.3437 | 0.1218 | 1.01E-01 | 20.61 |
| IL-18 | Lung adenocarcinoma | rs150005227 | T | C | 0.01 | 0.4349 | 0.0889 | 8.89E-07 | 0.01 | 0.3785 | 0.2327 | 4.76E-02 | 23.93 |
| IL-18 | Lung adenocarcinoma | rs4482818 | A | G | 0.64 | 0.1286 | 0.0244 | 1.45E-07 | 0.63 | -0.0181 | 0.0315 | 5.70E-01 | 27.78 |
| IL-18 | Lung adenocarcinoma | rs658805 | A | G | 0.32 | 0.1226 | 0.0244 | 4.94E-07 | 0.31 | -0.0299 | 0.0286 | 3.25E-01 | 25.25 |
| IL-18 | Lung adenocarcinoma | rs78716465 | A | G | 0.04 | 0.3265 | 0.0682 | 1.63E-06 | 0.04 | 0.0084 | 0.0766 | 9.19E-01 | 22.92 |
| IL-18 | Squamous cell lung cancer | rs11214140 | C | T | 0.18 | 0.1531 | 0.0302 | 3.68E-07 | 0.17 | -0.0388 | 0.0407 | 3.80E-01 | 25.70 |
| IL-18 | Squamous cell lung cancer | rs116383510 | C | A | 0.02 | 0.5426 | 0.1056 | 3.00E-07 | 0.01 | 0.1346 | 0.1380 | 3.28E-01 | 26.40 |
| IL-18 | Squamous cell lung cancer | rs117266781 | T | C | 0.01 | 0.6841 | 0.1468 | 3.15E-06 | 0.01 | 0.1322 | 0.1392 | 3.43E-01 | 21.72 |
| IL-18 | Squamous cell lung cancer | rs144841621 | T | C | 0.01 | 0.5180 | 0.1141 | 3.81E-06 | 0.01 | 0.0334 | 0.1578 | 8.54E-01 | 20.61 |
| IL-18 | Squamous cell lung cancer | rs150005227 | T | C | 0.01 | 0.4349 | 0.0889 | 8.89E-07 | 0.01 | 0.1427 | 0.1764 | 4.33E-01 | 23.93 |
| IL-18 | Squamous cell lung cancer | rs4482818 | A | G | 0.64 | 0.1286 | 0.0244 | 1.45E-07 | 0.63 | -0.0104 | 0.0312 | 7.44E-01 | 27.78 |
| IL-18 | Squamous cell lung cancer | rs658805 | A | G | 0.32 | 0.1226 | 0.0244 | 4.94E-07 | 0.31 | -0.0422 | 0.0283 | 1.66E-01 | 25.25 |
| IL-18 | Squamous cell lung cancer | rs78716465 | A | G | 0.04 | 0.3265 | 0.0682 | 1.63E-06 | 0.04 | 0.1080 | 0.0841 | 1.86E-01 | 22.92 |
| IL-1β | Lung cancer | rs1942793 | T | G | 0.47 | 0.0717 | 0.0187 | 4.98E-06 | 0.44 | 0.0305 | 0.0185 | 9.45E-02 | 14.70 |
| IL-1β | Lung cancer | rs4786740 | A | C | 0.41 | 0.0845 | 0.0202 | 4.67E-06 | 0.44 | -0.0313 | 0.0199 | 1.35E-01 | 17.50 |
| IL-1β | Lung cancer | rs62015704 | A | G | 0.88 | 0.1082 | 0.0283 | 2.09E-06 | 0.88 | -0.0237 | 0.0298 | 4.28E-01 | 14.62 |
| IL-1β | Lung adenocarcinoma | rs1942793 | T | G | 0.47 | 0.0717 | 0.0187 | 4.98E-06 | 0.44 | 0.0521 | 0.0288 | 6.41E-02 | 14.70 |
| IL-1β | Lung adenocarcinoma | rs4786740 | A | C | 0.41 | 0.0845 | 0.0202 | 4.67E-06 | 0.44 | -0.0059 | 0.0314 | 8.56E-01 | 17.50 |
| IL-1β | Lung adenocarcinoma | rs62015704 | A | G | 0.88 | 0.1082 | 0.0283 | 2.09E-06 | 0.88 | -0.0718 | 0.0479 | 1.23E-01 | 14.62 |
| IL-1β | Squamous cell lung cancer | rs1942793 | T | G | 0.47 | 0.0717 | 0.0187 | 4.98E-06 | 0.44 | 0.0482 | 0.0287 | 8.67E-02 | 14.70 |
| IL-1β | Squamous cell lung cancer | rs4786740 | A | C | 0.41 | 0.0845 | 0.0202 | 4.67E-06 | 0.44 | -0.0694 | 0.0296 | 3.40E-02 | 17.50 |
| IL-1β | Squamous cell lung cancer | rs62015704 | A | G | 0.88 | 0.1082 | 0.0283 | 2.09E-06 | 0.88 | -0.0494 | 0.0465 | 2.87E-01 | 14.62 |
| IL-1Ra | Lung cancer | rs1054402 | T | C | 0.26 | 0.1311 | 0.0270 | 1.13E-06 | 0.26 | -0.0029 | 0.0205 | 8.91E-01 | 23.58 |
| IL-1Ra | Lung cancer | rs11627423 | A | C | 0.61 | 0.1171 | 0.0247 | 2.12E-06 | 0.60 | -0.0004 | 0.0181 | 9.83E-01 | 22.48 |
| IL-1Ra | Lung cancer | rs2809154 | C | T | 0.82 | 0.1786 | 0.0388 | 3.74E-06 | 0.82 | 0.0029 | 0.0241 | 9.08E-01 | 21.19 |
| IL-1Ra | Lung adenocarcinoma | rs1054402 | T | C | 0.26 | 0.1311 | 0.0270 | 1.13E-06 | 0.26 | 0.0220 | 0.0321 | 4.96E-01 | 23.58 |
| IL-1Ra | Lung adenocarcinoma | rs11627423 | A | C | 0.61 | 0.1171 | 0.0247 | 2.12E-06 | 0.60 | 0.0222 | 0.0272 | 4.37E-01 | 22.48 |
| IL-1Ra | Lung adenocarcinoma | rs2809154 | C | T | 0.82 | 0.1786 | 0.0388 | 3.74E-06 | 0.82 | -0.0415 | 0.0376 | 2.68E-01 | 21.19 |
| IL-1Ra | Squamous cell lung cancer | rs1054402 | T | C | 0.26 | 0.1311 | 0.0270 | 1.13E-06 | 0.26 | -0.0164 | 0.0312 | 6.16E-01 | 23.58 |
| IL-1Ra | Squamous cell lung cancer | rs11627423 | A | C | 0.61 | 0.1171 | 0.0247 | 2.12E-06 | 0.60 | -0.0397 | 0.0289 | 1.65E-01 | 22.48 |
| IL-1Ra | Squamous cell lung cancer | rs2809154 | C | T | 0.82 | 0.1786 | 0.0388 | 3.74E-06 | 0.83 | 0.0338 | 0.0364 | 3.87E-01 | 21.19 |
| IL-18BP | Lung cancer | rs12455835 | A | C | 0.02817 | 0.3451 | 0.0743 | 3.47E-06 | 0.03 | 0.0043 | 0.0539 | 9.39E-01 | 21.57 |
| IL-18BP | Lung cancer | rs146084600 | A | C | 0.01285 | -0.5333 | 0.1140 | 2.88E-06 | 0.01 | 0.0731 | 0.0958 | 4.55E-01 | 21.88 |
| IL-18BP | Lung cancer | rs1600079 | T | C | 0.74166 | -0.1337 | 0.0283 | 2.40E-06 | 0.74 | -0.0347 | 0.0214 | 9.89E-02 | 22.32 |
| IL-18BP | Lung cancer | rs34396416 | T | C | 0.01998 | -0.4187 | 0.0898 | 3.16E-06 | 0.02 | 0.2258 | 0.0956 | 6.30E-03 | 21.74 |
| IL-18BP | Lung cancer | rs62211692 | G | A | 0.23371 | 0.1460 | 0.0306 | 1.91E-06 | 0.23 | -0.0041 | 0.0232 | 8.64E-01 | 22.76 |
| IL-18BP | Lung cancer | rs76726938 | T | G | 0.03042 | 0.3713 | 0.0737 | 4.68E-07 | 0.02 | -0.0944 | 0.0584 | 1.69E-01 | 25.38 |
| IL-18BP | Lung adenocarcinoma | rs12455835 | A | C | 0.02817 | 0.3451 | 0.0743 | 3.47E-06 | 0.03 | -0.0833 | 0.0740 | 3.41E-01 | 21.57 |
| IL-18BP | Lung adenocarcinoma | rs146084600 | A | C | 0.01285 | -0.5333 | 0.1140 | 2.88E-06 | 0.01 | 0.0031 | 0.1298 | 9.83E-01 | 21.88 |
| IL-18BP | Lung adenocarcinoma | rs1600079 | T | C | 0.74166 | -0.1337 | 0.0283 | 2.40E-06 | 0.74 | -0.0071 | 0.0320 | 8.27E-01 | 22.32 |
| IL-18BP | Lung adenocarcinoma | rs34396416 | T | C | 0.01998 | -0.4187 | 0.0898 | 3.16E-06 | 0.02 | 0.2709 | 0.1501 | 3.66E-02 | 21.74 |
| IL-18BP | Lung adenocarcinoma | rs62211692 | G | A | 0.23371 | 0.1460 | 0.0306 | 1.91E-06 | 0.23 | 0.0160 | 0.0358 | 6.61E-01 | 22.76 |
| IL-18BP | Lung adenocarcinoma | rs76726938 | T | G | 0.03042 | 0.3713 | 0.0737 | 4.68E-07 | 0.02 | -0.0374 | 0.0887 | 7.12E-01 | 25.38 |
| IL-18BP | Squamous cell lung cancer | rs12455835 | A | C | 0.02817 | 0.3451 | 0.0743 | 3.47E-06 | 0.03 | -0.1505 | 0.0711 | 9.51E-02 | 21.57 |
| IL-18BP | Squamous cell lung cancer | rs146084600 | A | C | 0.01285 | -0.5333 | 0.1140 | 2.88E-06 | 0.01 | 0.0624 | 0.1445 | 6.93E-01 | 21.88 |
| IL-18BP | Squamous cell lung cancer | rs1600079 | T | C | 0.74166 | -0.1337 | 0.0283 | 2.40E-06 | 0.74 | -0.0450 | 0.0330 | 1.67E-01 | 22.32 |
| IL-18BP | Squamous cell lung cancer | rs34396416 | T | C | 0.01998 | -0.4187 | 0.0898 | 3.16E-06 | 0.02 | 0.1159 | 0.1297 | 3.76E-01 | 21.74 |
| IL-18BP | Squamous cell lung cancer | rs62211692 | G | A | 0.23371 | 0.1460 | 0.0306 | 1.91E-06 | 0.22 | -0.0101 | 0.0355 | 7.85E-01 | 22.76 |
| IL-18BP | Squamous cell lung cancer | rs76726938 | T | G | 0.03042 | 0.3713 | 0.0737 | 4.68E-07 | 0.02 | -0.1196 | 0.0857 | 2.64E-01 | 25.38 |
| IL-18Rα | Lung cancer | rs11165484 | T | C | 0.26336 | -0.1400 | 0.0283 | 7.76E-07 | 0.27 | 0.0117 | 0.0202 | 5.67E-01 | 24.47 |
| IL-18Rα | Lung cancer | rs111686801 | A | G | 0.12997 | 0.1770 | 0.0380 | 3.31E-06 | 0.13 | 0.0075 | 0.0278 | 7.91E-01 | 21.70 |
| IL-18Rα | Lung cancer | rs139519085 | G | T | 0.03566 | 0.3389 | 0.0728 | 3.24E-06 | 0.02 | 0.1234 | 0.0764 | 8.87E-02 | 21.67 |
| IL-18Rα | Lung cancer | rs2429175 | A | G | 0.21764 | 0.1444 | 0.0303 | 1.86E-06 | 0.22 | -0.0294 | 0.0207 | 1.76E-01 | 22.71 |
| IL-18Rα | Lung cancer | rs34988270 | T | C | 0.06529 | 0.2364 | 0.0507 | 3.09E-06 | 0.06 | 0.0893 | 0.0461 | 4.21E-02 | 21.74 |
| IL-18Rα | Lung cancer | rs58514453 | C | T | 0.03345 | -0.3248 | 0.0705 | 3.98E-06 | 0.02 | -0.0425 | 0.0588 | 5.15E-01 | 21.23 |
| IL-18Rα | Lung cancer | rs61929078 | A | G | 0.00959 | 0.6031 | 0.1319 | 4.79E-06 | 0.01 | 0.0813 | 0.1221 | 5.23E-01 | 20.91 |
| IL-18Rα | Lung cancer | rs6814269 | G | A | 0.34744 | 0.1261 | 0.0259 | 1.12E-06 | 0.37 | 0.0115 | 0.0194 | 5.56E-01 | 23.70 |
| IL-18Rα | Lung cancer | rs79171645 | T | G | 0.05279 | -0.2894 | 0.0588 | 8.71E-07 | 0.04 | -0.0053 | 0.0521 | 9.23E-01 | 24.22 |
| IL-18Rα | Lung adenocarcinoma | rs11165484 | T | C | 0.26336 | -0.1400 | 0.0283 | 7.76E-07 | 0.27 | 0.0141 | 0.0314 | 6.58E-01 | 24.47 |
| IL-18Rα | Lung adenocarcinoma | rs111686801 | A | G | 0.12997 | 0.1770 | 0.0380 | 3.31E-06 | 0.13 | 0.0353 | 0.0434 | 4.20E-01 | 21.70 |
| IL-18Rα | Lung adenocarcinoma | rs139519085 | G | T | 0.03566 | 0.3389 | 0.0728 | 3.24E-06 | 0.02 | 0.3547 | 0.1409 | 1.25E-03 | 21.67 |
| IL-18Rα | Lung adenocarcinoma | rs2429175 | A | G | 0.21764 | 0.1444 | 0.0303 | 1.86E-06 | 0.22 | -0.0116 | 0.0320 | 7.29E-01 | 22.71 |
| IL-18Rα | Lung adenocarcinoma | rs34988270 | T | C | 0.06529 | 0.2364 | 0.0507 | 3.09E-06 | 0.06 | 0.0668 | 0.0682 | 3.27E-01 | 21.74 |
| IL-18Rα | Lung adenocarcinoma | rs58514453 | C | T | 0.03345 | -0.3248 | 0.0705 | 3.98E-06 | 0.02 | -0.0162 | 0.0895 | 8.72E-01 | 21.23 |
| IL-18Rα | Lung adenocarcinoma | rs61929078 | A | G | 0.00959 | 0.6031 | 0.1319 | 4.79E-06 | 0.01 | 0.3540 | 0.2223 | 5.73E-02 | 20.91 |
| IL-18Rα | Lung adenocarcinoma | rs6814269 | G | A | 0.34744 | 0.1261 | 0.0259 | 1.12E-06 | 0.37 | 0.0029 | 0.0294 | 9.25E-01 | 23.70 |
| IL-18Rα | Lung adenocarcinoma | rs79171645 | T | G | 0.05279 | -0.2894 | 0.0588 | 8.71E-07 | 0.04 | -0.0551 | 0.0752 | 5.23E-01 | 24.22 |
| IL-18Rα | Squamous cell lung cancer | rs11165484 | T | C | 0.26336 | -0.1400 | 0.0283 | 7.76E-07 | 0.27 | 0.0278 | 0.0315 | 3.79E-01 | 24.47 |
| IL-18Rα | Squamous cell lung cancer | rs111686801 | A | G | 0.12997 | 0.1770 | 0.0380 | 3.31E-06 | 0.13 | -0.0028 | 0.0422 | 9.49E-01 | 21.70 |
| IL-18Rα | Squamous cell lung cancer | rs139519085 | G | T | 0.03566 | 0.3389 | 0.0728 | 3.24E-06 | 0.02 | -0.2629 | 0.0827 | 2.96E-02 | 21.67 |
| IL-18Rα | Squamous cell lung cancer | rs2429175 | A | G | 0.21764 | 0.1444 | 0.0303 | 1.86E-06 | 0.22 | -0.0426 | 0.0311 | 2.04E-01 | 22.71 |
| IL-18Rα | Squamous cell lung cancer | rs34988270 | T | C | 0.06529 | 0.2364 | 0.0507 | 3.09E-06 | 0.06 | 0.1999 | 0.0786 | 3.67E-03 | 21.74 |
| IL-18Rα | Squamous cell lung cancer | rs58514453 | C | T | 0.03345 | -0.3248 | 0.0705 | 3.98E-06 | 0.02 | -0.0059 | 0.0936 | 9.55E-01 | 21.23 |
| IL-18Rα | Squamous cell lung cancer | rs61929078 | A | G | 0.00959 | 0.6031 | 0.1319 | 4.79E-06 | 0.01 | -0.0393 | 0.1629 | 8.49E-01 | 20.91 |
| IL-18Rα | Squamous cell lung cancer | rs6814269 | G | A | 0.34744 | 0.1261 | 0.0259 | 1.12E-06 | 0.37 | 0.0437 | 0.0308 | 1.50E-01 | 23.70 |
| IL-18Rα | Squamous cell lung cancer | rs79171645 | T | G | 0.05279 | -0.2894 | 0.0588 | 8.71E-07 | 0.04 | 0.0002 | 0.0796 | 9.98E-01 | 24.22 |
| IL-1α | Lung cancer | rs11922046 | T | C | 0.25896 | 0.1392 | 0.0287 | 1.20E-06 | 0.25 | -0.0120 | 0.0211 | 5.81E-01 | 23.52 |
| IL-1α | Lung cancer | rs139711468 | A | G | 0.0337 | -0.3470 | 0.0728 | 1.86E-06 | 0.02 | 0.1312 | 0.0678 | 3.81E-02 | 22.72 |
| IL-1α | Lung cancer | rs2734376 | T | C | 0.68793 | 0.1397 | 0.0278 | 4.90E-07 | 0.68 | 0.0330 | 0.0186 | 9.33E-02 | 25.25 |
| IL-1α | Lung cancer | rs28895307 | G | T | 0.03048 | 0.3649 | 0.0721 | 4.27E-07 | 0.03 | 0.1752 | 0.0746 | 8.68E-03 | 25.61 |
| IL-1α | Lung cancer | rs41386050 | T | G | 0.03382 | 0.3175 | 0.0694 | 4.79E-06 | 0.02 | 0.0924 | 0.0696 | 1.73E-01 | 20.93 |
| IL-1α | Lung cancer | rs4867603 | C | T | 0.55418 | -0.1305 | 0.0267 | 1.05E-06 | 0.56 | -0.0103 | 0.0207 | 6.24E-01 | 23.89 |
| IL-1α | Lung cancer | rs6549977 | A | G | 0.38247 | -0.1196 | 0.0259 | 3.89E-06 | 0.42 | -0.0085 | 0.0178 | 6.41E-01 | 21.32 |
| IL-1α | Lung cancer | rs7148359 | T | C | 0.26768 | -0.1354 | 0.0286 | 2.14E-06 | 0.26 | 0.0046 | 0.0229 | 8.43E-01 | 22.41 |
| IL-1α | Lung cancer | rs8107140 | A | C | 0.59218 | 0.1147 | 0.0248 | 3.63E-06 | 0.59 | -0.0124 | 0.0188 | 5.14E-01 | 21.39 |
| IL-1α | Lung adenocarcinoma | rs11922046 | T | C | 0.25896 | 0.1392 | 0.0287 | 1.20E-06 | 0.25 | 0.0148 | 0.0331 | 6.60E-01 | 23.52 |
| IL-1α | Lung adenocarcinoma | rs139711468 | A | G | 0.0337 | -0.3470 | 0.0728 | 1.86E-06 | 0.02 | 0.2039 | 0.1094 | 3.77E-02 | 22.72 |
| IL-1α | Lung adenocarcinoma | rs2734376 | T | C | 0.68793 | 0.1397 | 0.0278 | 4.90E-07 | 0.68 | 0.0674 | 0.0274 | 2.57E-02 | 25.25 |
| IL-1α | Lung adenocarcinoma | rs28895307 | G | T | 0.03048 | 0.3649 | 0.0721 | 4.27E-07 | 0.03 | 0.1384 | 0.1128 | 2.05E-01 | 25.61 |
| IL-1α | Lung adenocarcinoma | rs41386050 | T | G | 0.03382 | 0.3175 | 0.0694 | 4.79E-06 | 0.02 | 0.1384 | 0.1112 | 1.97E-01 | 20.93 |
| IL-1α | Lung adenocarcinoma | rs4867603 | C | T | 0.55418 | -0.1305 | 0.0267 | 1.05E-06 | 0.56 | -0.0179 | 0.0321 | 5.82E-01 | 23.89 |
| IL-1α | Lung adenocarcinoma | rs6549977 | A | G | 0.38247 | -0.1196 | 0.0259 | 3.89E-06 | 0.42 | -0.0545 | 0.0261 | 5.43E-02 | 21.32 |
| IL-1α | Lung adenocarcinoma | rs7148359 | T | C | 0.26768 | -0.1354 | 0.0286 | 2.14E-06 | 0.26 | 0.0130 | 0.0354 | 7.21E-01 | 22.41 |
| IL-1α | Lung adenocarcinoma | rs8107140 | A | C | 0.59218 | 0.1147 | 0.0248 | 3.63E-06 | 0.59 | -0.0024 | 0.0287 | 9.36E-01 | 21.39 |
| IL-1α | Squamous cell lung cancer | rs11922046 | T | C | 0.25896 | 0.1392 | 0.0287 | 1.20E-06 | 0.25 | -0.0642 | 0.0309 | 5.92E-02 | 23.52 |
| IL-1α | Squamous cell lung cancer | rs139711468 | A | G | 0.0337 | -0.3470 | 0.0728 | 1.86E-06 | 0.02 | 0.1154 | 0.1021 | 2.49E-01 | 22.72 |
| IL-1α | Squamous cell lung cancer | rs2734376 | T | C | 0.68793 | 0.1397 | 0.0278 | 4.90E-07 | 0.69 | 0.0273 | 0.0290 | 3.75E-01 | 25.25 |
| IL-1α | Squamous cell lung cancer | rs28895307 | G | T | 0.03048 | 0.3649 | 0.0721 | 4.27E-07 | 0.03 | 0.1455 | 0.1106 | 1.69E-01 | 25.61 |
| IL-1α | Squamous cell lung cancer | rs41386050 | T | G | 0.03382 | 0.3175 | 0.0694 | 4.79E-06 | 0.02 | -0.0532 | 0.0939 | 6.29E-01 | 20.93 |
| IL-1α | Squamous cell lung cancer | rs4867603 | C | T | 0.55418 | -0.1305 | 0.0267 | 1.05E-06 | 0.56 | -0.0221 | 0.0325 | 5.00E-01 | 23.89 |
| IL-1α | Squamous cell lung cancer | rs6549977 | A | G | 0.38247 | -0.1196 | 0.0259 | 3.89E-06 | 0.42 | 0.0458 | 0.0288 | 1.06E-01 | 21.32 |
| IL-1α | Squamous cell lung cancer | rs7148359 | T | C | 0.26768 | -0.1354 | 0.0286 | 2.14E-06 | 0.26 | 0.0216 | 0.0360 | 5.54E-01 | 22.41 |
| IL-1α | Squamous cell lung cancer | rs8107140 | A | C | 0.59218 | 0.1147 | 0.0248 | 3.63E-06 | 0.59 | -0.0276 | 0.0295 | 3.51E-01 | 21.39 |
| IL-1R1 | Lung cancer | rs143365592 | G | T | 0.01155 | 0.5682 | 0.1242 | 4.79E-06 | 0.01 | 0.0310 | 0.0944 | 7.58E-01 | 20.93 |
| IL-1R1 | Lung cancer | rs144792666 | T | C | 0.01578 | 0.4847 | 0.1006 | 1.45E-06 | 0.01 | 0.1020 | 0.1001 | 3.05E-01 | 23.21 |
| IL-1R1 | Lung cancer | rs145533598 | A | G | 0.01743 | 0.4698 | 0.0965 | 1.12E-06 | 0.02 | -0.0346 | 0.0753 | 6.83E-01 | 23.70 |
| IL-1R1 | Lung cancer | rs1652049 | G | T | 0.45582 | 0.1284 | 0.0268 | 1.62E-06 | 0.43 | 0.0057 | 0.0217 | 7.97E-01 | 22.95 |
| IL-1R1 | Lung cancer | rs71358078 | T | C | 0.02184 | -0.4061 | 0.0852 | 1.86E-06 | 0.02 | -0.1004 | 0.0564 | 1.31E-01 | 22.72 |
| IL-1R1 | Lung cancer | rs72771293 | G | A | 0.21975 | 0.1504 | 0.0310 | 1.17E-06 | 0.21 | 0.0052 | 0.0231 | 8.25E-01 | 23.54 |
| IL-1R1 | Lung cancer | rs76053345 | C | T | 0.0064 | -0.7564 | 0.1567 | 1.38E-06 | 0.01 | 0.0361 | 0.1237 | 7.90E-01 | 23.30 |
| IL-1R1 | Lung cancer | rs78801677 | A | G | 0.0889 | 0.2060 | 0.0443 | 3.31E-06 | 0.10 | -0.0061 | 0.0362 | 8.72E-01 | 21.62 |
| IL-1R1 | Lung adenocarcinoma | rs143365592 | G | T | 0.01155 | 0.5682 | 0.1242 | 4.79E-06 | 0.01 | -0.1817 | 0.1178 | 2.72E-01 | 20.93 |
| IL-1R1 | Lung adenocarcinoma | rs144792666 | T | C | 0.01578 | 0.4847 | 0.1006 | 1.45E-06 | 0.01 | 0.0451 | 0.1351 | 7.62E-01 | 23.21 |
| IL-1R1 | Lung adenocarcinoma | rs145533598 | A | G | 0.01743 | 0.4698 | 0.0965 | 1.12E-06 | 0.02 | -0.0480 | 0.1124 | 7.20E-01 | 23.70 |
| IL-1R1 | Lung adenocarcinoma | rs1652049 | G | T | 0.45582 | 0.1284 | 0.0268 | 1.62E-06 | 0.43 | 0.0247 | 0.0339 | 4.70E-01 | 22.95 |
| IL-1R1 | Lung adenocarcinoma | rs71358078 | T | C | 0.02184 | -0.4061 | 0.0852 | 1.86E-06 | 0.02 | -0.0534 | 0.0869 | 5.97E-01 | 22.72 |
| IL-1R1 | Lung adenocarcinoma | rs72771293 | G | A | 0.21975 | 0.1504 | 0.0310 | 1.17E-06 | 0.21 | 0.0211 | 0.0357 | 5.61E-01 | 23.54 |
| IL-1R1 | Lung adenocarcinoma | rs76053345 | C | T | 0.0064 | -0.7564 | 0.1567 | 1.38E-06 | 0.01 | 0.0424 | 0.1768 | 8.37E-01 | 23.30 |
| IL-1R1 | Lung adenocarcinoma | rs78801677 | A | G | 0.0889 | 0.2060 | 0.0443 | 3.31E-06 | 0.10 | 0.0423 | 0.0568 | 4.62E-01 | 21.62 |
| IL-1R1 | Squamous cell lung cancer | rs143365592 | G | T | 0.01155 | 0.5682 | 0.1242 | 4.79E-06 | 0.01 | 0.0859 | 0.1428 | 5.71E-01 | 20.93 |
| IL-1R1 | Squamous cell lung cancer | rs144792666 | T | C | 0.01578 | 0.4847 | 0.1006 | 1.45E-06 | 0.01 | 0.0776 | 0.1506 | 6.34E-01 | 23.21 |
| IL-1R1 | Squamous cell lung cancer | rs145533598 | A | G | 0.01743 | 0.4698 | 0.0965 | 1.12E-06 | 0.02 | 0.0593 | 0.1233 | 6.53E-01 | 23.70 |
| IL-1R1 | Squamous cell lung cancer | rs1652049 | G | T | 0.45582 | 0.1284 | 0.0268 | 1.62E-06 | 0.43 | 0.0236 | 0.0341 | 4.94E-01 | 22.95 |
| IL-1R1 | Squamous cell lung cancer | rs71358078 | T | C | 0.02184 | -0.4061 | 0.0852 | 1.86E-06 | 0.02 | -0.1178 | 0.0832 | 2.55E-01 | 22.72 |
| IL-1R1 | Squamous cell lung cancer | rs72771293 | G | A | 0.21975 | 0.1504 | 0.0310 | 1.17E-06 | 0.21 | 0.0171 | 0.0361 | 6.41E-01 | 23.54 |
| IL-1R1 | Squamous cell lung cancer | rs76053345 | C | T | 0.0064 | -0.7564 | 0.1567 | 1.38E-06 | 0.01 | 0.0593 | 0.1861 | 7.83E-01 | 23.30 |
| IL-1R1 | Squamous cell lung cancer | rs78801677 | A | G | 0.0889 | 0.2060 | 0.0443 | 3.31E-06 | 0.09 | -0.0301 | 0.0543 | 6.11E-01 | 21.62 |
| IL-1Racp | Lung cancer | rs113553332 | T | C | 0.04838 | 0.6851 | 0.0563 | 4.68E-34 | 0.04 | 0.1412 | 0.0553 | 5.11E-03 | 148.08 |
| IL-1Racp | Lung cancer | rs1607264 | C | A | 0.8005 | -0.1455 | 0.0311 | 2.82E-06 | 0.80 | -0.0190 | 0.0225 | 4.00E-01 | 21.89 |
| IL-1Racp | Lung cancer | rs16969649 | T | C | 0.20827 | -0.1400 | 0.0306 | 4.90E-06 | 0.20 | -0.0611 | 0.0207 | 6.61E-03 | 20.93 |
| IL-1Racp | Lung cancer | rs2885370 | T | G | 0.74273 | -0.2086 | 0.0306 | 8.91E-12 | 0.75 | 0.0273 | 0.0201 | 1.95E-01 | 46.47 |
| IL-1Racp | Lung cancer | rs3935774 | T | C | 0.01707 | 0.7411 | 0.1119 | 3.55E-11 | 0.01 | 0.0980 | 0.1040 | 3.47E-01 | 43.86 |
| IL-1Racp | Lung cancer | rs62196769 | C | A | 0.08705 | -0.2046 | 0.0439 | 3.09E-06 | 0.09 | 0.0130 | 0.0304 | 6.74E-01 | 21.72 |
| IL-1Racp | Lung cancer | rs6444430 | A | G | 0.78955 | 0.1543 | 0.0326 | 2.29E-06 | 0.79 | -0.0174 | 0.0275 | 5.31E-01 | 22.40 |
| IL-1Racp | Lung cancer | rs6461953 | C | T | 0.18151 | -0.1459 | 0.0319 | 4.68E-06 | 0.18 | 0.0239 | 0.0243 | 3.25E-01 | 20.92 |
| IL-1Racp | Lung cancer | rs67249092 | G | A | 0.15199 | -0.3790 | 0.0345 | 5.25E-28 | 0.16 | -0.0494 | 0.0242 | 5.82E-02 | 120.68 |
| IL-1Racp | Lung cancer | rs6801017 | G | A | 0.21399 | -0.2023 | 0.0306 | 3.80E-11 | 0.20 | -0.0170 | 0.0224 | 4.64E-01 | 43.71 |
| IL-1Racp | Lung cancer | rs73754360 | G | T | 0.02695 | 0.3836 | 0.0803 | 1.78E-06 | 0.03 | -0.0481 | 0.0533 | 4.17E-01 | 22.82 |
| IL-1Racp | Lung cancer | rs75857200 | A | C | 0.09995 | 0.1985 | 0.0420 | 2.34E-06 | 0.12 | 0.0366 | 0.0290 | 2.03E-01 | 22.34 |
| IL-1Racp | Lung cancer | rs7623802 | T | C | 0.66389 | -0.1290 | 0.0267 | 1.41E-06 | 0.68 | -0.0231 | 0.0193 | 2.29E-01 | 23.34 |
| IL-1Racp | Lung adenocarcinoma | rs113553332 | T | C | 0.04838 | 0.6851 | 0.0563 | 4.68E-34 | 0.04 | 0.0387 | 0.0752 | 6.20E-01 | 148.08 |
| IL-1Racp | Lung adenocarcinoma | rs1607264 | C | A | 0.8005 | -0.1455 | 0.0311 | 2.82E-06 | 0.80 | -0.0933 | 0.0367 | 6.93E-03 | 21.89 |
| IL-1Racp | Lung adenocarcinoma | rs16969649 | T | C | 0.20827 | -0.1400 | 0.0306 | 4.90E-06 | 0.21 | -0.0732 | 0.0313 | 3.56E-02 | 20.93 |
| IL-1Racp | Lung adenocarcinoma | rs2885370 | T | G | 0.74273 | -0.2086 | 0.0306 | 8.91E-12 | 0.75 | 0.0190 | 0.0309 | 5.60E-01 | 46.47 |
| IL-1Racp | Lung adenocarcinoma | rs3935774 | T | C | 0.01707 | 0.7411 | 0.1119 | 3.55E-11 | 0.01 | 0.3167 | 0.1845 | 4.24E-02 | 43.86 |
| IL-1Racp | Lung adenocarcinoma | rs62196769 | C | A | 0.08705 | -0.2046 | 0.0439 | 3.09E-06 | 0.09 | 0.0367 | 0.0479 | 4.48E-01 | 21.72 |
| IL-1Racp | Lung adenocarcinoma | rs6444430 | A | G | 0.78955 | 0.1543 | 0.0326 | 2.29E-06 | 0.79 | -0.0514 | 0.0444 | 2.43E-01 | 22.40 |
| IL-1Racp | Lung adenocarcinoma | rs6461953 | C | T | 0.18151 | -0.1459 | 0.0319 | 4.68E-06 | 0.18 | 0.0122 | 0.0366 | 7.46E-01 | 20.92 |
| IL-1Racp | Lung adenocarcinoma | rs67249092 | G | A | 0.15199 | -0.3790 | 0.0345 | 5.25E-28 | 0.16 | -0.0346 | 0.0374 | 3.91E-01 | 120.68 |
| IL-1Racp | Lung adenocarcinoma | rs6801017 | G | A | 0.21399 | -0.2023 | 0.0306 | 3.80E-11 | 0.20 | -0.0330 | 0.0334 | 3.56E-01 | 43.71 |
| IL-1Racp | Lung adenocarcinoma | rs73754360 | G | T | 0.02695 | 0.3836 | 0.0803 | 1.78E-06 | 0.03 | -0.0657 | 0.0769 | 4.63E-01 | 22.82 |
| IL-1Racp | Lung adenocarcinoma | rs75857200 | A | C | 0.09995 | 0.1985 | 0.0420 | 2.34E-06 | 0.12 | 0.0408 | 0.0446 | 3.61E-01 | 22.34 |
| IL-1Racp | Lung adenocarcinoma | rs7623802 | T | C | 0.66389 | -0.1290 | 0.0267 | 1.41E-06 | 0.68 | 0.0454 | 0.0275 | 1.26E-01 | 23.34 |
| IL-1Racp | Squamous cell lung cancer | rs113553332 | T | C | 0.04838 | 0.6851 | 0.0563 | 4.68E-34 | 0.04 | 0.1429 | 0.0859 | 7.58E-02 | 148.08 |
| IL-1Racp | Squamous cell lung cancer | rs1607264 | C | A | 0.8005 | -0.1455 | 0.0311 | 2.82E-06 | 0.80 | 0.0276 | 0.0331 | 4.33E-01 | 21.89 |
| IL-1Racp | Squamous cell lung cancer | rs16969649 | T | C | 0.20827 | -0.1400 | 0.0306 | 4.90E-06 | 0.20 | -0.0526 | 0.0319 | 1.30E-01 | 20.93 |
| IL-1Racp | Squamous cell lung cancer | rs2885370 | T | G | 0.74273 | -0.2086 | 0.0306 | 8.91E-12 | 0.75 | 0.0087 | 0.0312 | 7.89E-01 | 46.47 |
| IL-1Racp | Squamous cell lung cancer | rs3935774 | T | C | 0.01707 | 0.7411 | 0.1119 | 3.55E-11 | 0.01 | 0.0854 | 0.1507 | 5.97E-01 | 43.86 |
| IL-1Racp | Squamous cell lung cancer | rs62196769 | C | A | 0.08705 | -0.2046 | 0.0439 | 3.09E-06 | 0.09 | 0.0159 | 0.0463 | 7.39E-01 | 21.72 |
| IL-1Racp | Squamous cell lung cancer | rs6444430 | A | G | 0.78955 | 0.1543 | 0.0326 | 2.29E-06 | 0.79 | 0.0225 | 0.0404 | 6.01E-01 | 22.40 |
| IL-1Racp | Squamous cell lung cancer | rs6461953 | C | T | 0.18151 | -0.1459 | 0.0319 | 4.68E-06 | 0.18 | 0.0364 | 0.0378 | 3.37E-01 | 20.92 |
| IL-1Racp | Squamous cell lung cancer | rs67249092 | G | A | 0.15199 | -0.3790 | 0.0345 | 5.25E-28 | 0.16 | -0.0963 | 0.0357 | 1.88E-02 | 120.68 |
| IL-1Racp | Squamous cell lung cancer | rs6801017 | G | A | 0.21399 | -0.2023 | 0.0306 | 3.80E-11 | 0.20 | -0.0020 | 0.0349 | 9.55E-01 | 43.71 |
| IL-1Racp | Squamous cell lung cancer | rs73754360 | G | T | 0.02695 | 0.3836 | 0.0803 | 1.78E-06 | 0.03 | -0.1229 | 0.0746 | 1.83E-01 | 22.82 |
| IL-1Racp | Squamous cell lung cancer | rs75857200 | A | C | 0.09995 | 0.1985 | 0.0420 | 2.34E-06 | 0.12 | 0.1021 | 0.0474 | 2.26E-02 | 22.34 |
| IL-1Racp | Squamous cell lung cancer | rs7623802 | T | C | 0.66389 | -0.1290 | 0.0267 | 1.41E-06 | 0.67 | -0.0841 | 0.0313 | 4.47E-03 | 23.34 |
| IL-36α | Lung cancer | rs10756815 | A | G | 0.85177 | 0.1788 | 0.0350 | 3.24E-07 | 0.85 | -0.0008 | 0.0246 | 9.75E-01 | 26.10 |
| IL-36α | Lung cancer | rs2072162 | A | G | 0.39357 | -0.1193 | 0.0259 | 4.07E-06 | 0.39 | 0.0130 | 0.0184 | 4.82E-01 | 21.22 |
| IL-36α | Lung cancer | rs62517456 | G | A | 0.08121 | 0.2190 | 0.0457 | 1.66E-06 | 0.08 | 0.0270 | 0.0348 | 4.40E-01 | 22.96 |
| IL-36α | Lung cancer | rs6767738 | G | A | 0.28427 | -0.1287 | 0.0277 | 3.55E-06 | 0.30 | -0.0204 | 0.0190 | 3.00E-01 | 21.59 |
| IL-36α | Lung adenocarcinoma | rs10756815 | A | G | 0.85177 | 0.1788 | 0.0350 | 3.24E-07 | 0.85 | -0.0425 | 0.0388 | 2.71E-01 | 26.10 |
| IL-36α | Lung adenocarcinoma | rs2072162 | A | G | 0.39357 | -0.1193 | 0.0259 | 4.07E-06 | 0.39 | 0.0058 | 0.0282 | 8.42E-01 | 21.22 |
| IL-36α | Lung adenocarcinoma | rs62517456 | G | A | 0.08121 | 0.2190 | 0.0457 | 1.66E-06 | 0.08 | 0.0570 | 0.0542 | 2.91E-01 | 22.96 |
| IL-36α | Lung adenocarcinoma | rs6767738 | G | A | 0.28427 | -0.1287 | 0.0277 | 3.55E-06 | 0.30 | -0.0187 | 0.0291 | 5.41E-01 | 21.59 |
| IL-36α | Squamous cell lung cancer | rs10756815 | A | G | 0.85177 | 0.1788 | 0.0350 | 3.24E-07 | 0.85 | 0.0051 | 0.0372 | 8.95E-01 | 26.10 |
| IL-36α | Squamous cell lung cancer | rs2072162 | A | G | 0.39357 | -0.1193 | 0.0259 | 4.07E-06 | 0.39 | 0.0186 | 0.0284 | 5.16E-01 | 21.22 |
| IL-36α | Squamous cell lung cancer | rs62517456 | G | A | 0.08121 | 0.2190 | 0.0457 | 1.66E-06 | 0.08 | -0.0018 | 0.0520 | 9.75E-01 | 22.96 |
| IL-36α | Squamous cell lung cancer | rs6767738 | G | A | 0.28427 | -0.1287 | 0.0277 | 3.55E-06 | 0.29 | -0.0458 | 0.0284 | 1.35E-01 | 21.59 |
| IL-36β | Lung cancer | rs11002612 | C | T | 0.07958 | -0.2157 | 0.0466 | 3.72E-06 | 0.07 | 0.0261 | 0.0422 | 5.43E-01 | 21.43 |
| IL-36β | Lung cancer | rs12191382 | C | T | 0.31824 | 0.1309 | 0.0263 | 6.61E-07 | 0.29 | 0.0116 | 0.0198 | 5.60E-01 | 24.77 |
| IL-36β | Lung cancer | rs16880182 | C | T | 0.05237 | -0.2690 | 0.0565 | 1.95E-06 | 0.05 | 0.0044 | 0.0405 | 9.17E-01 | 22.67 |
| IL-36β | Lung cancer | rs170187 | C | T | 0.74954 | -0.1327 | 0.0290 | 4.90E-06 | 0.76 | -0.0238 | 0.0211 | 2.58E-01 | 20.94 |
| IL-36β | Lung cancer | rs184204067 | T | C | 0.01986 | 0.4742 | 0.0952 | 6.31E-07 | 0.04 | -0.0019 | 0.0483 | 9.70E-01 | 24.81 |
| IL-36β | Lung cancer | rs188013949 | A | G | 0.01038 | 0.5876 | 0.1254 | 2.75E-06 | 0.01 | 0.0308 | 0.0966 | 7.66E-01 | 21.96 |
| IL-36β | Lung adenocarcinoma | rs11002612 | C | T | 0.07958 | -0.2157 | 0.0466 | 3.72E-06 | 0.07 | 0.0383 | 0.0649 | 5.66E-01 | 21.43 |
| IL-36β | Lung adenocarcinoma | rs12191382 | C | T | 0.31824 | 0.1309 | 0.0263 | 6.61E-07 | 0.29 | 0.0390 | 0.0311 | 2.05E-01 | 24.77 |
| IL-36β | Lung adenocarcinoma | rs16880182 | C | T | 0.05237 | -0.2690 | 0.0565 | 1.95E-06 | 0.05 | -0.0396 | 0.0582 | 5.39E-01 | 22.67 |
| IL-36β | Lung adenocarcinoma | rs170187 | C | T | 0.74954 | -0.1327 | 0.0290 | 4.90E-06 | 0.76 | -0.0605 | 0.0335 | 6.31E-02 | 20.94 |
| IL-36β | Lung adenocarcinoma | rs184204067 | T | C | 0.01986 | 0.4742 | 0.0952 | 6.31E-07 | 0.04 | 0.0405 | 0.0752 | 6.03E-01 | 24.81 |
| IL-36β | Lung adenocarcinoma | rs188013949 | A | G | 0.01038 | 0.5876 | 0.1254 | 2.75E-06 | 0.01 | 0.0619 | 0.1455 | 6.97E-01 | 21.96 |
| IL-36β | Squamous cell lung cancer | rs11002612 | C | T | 0.07958 | -0.2157 | 0.0466 | 3.72E-06 | 0.06 | -0.0120 | 0.0622 | 8.58E-01 | 21.43 |
| IL-36β | Squamous cell lung cancer | rs12191382 | C | T | 0.31824 | 0.1309 | 0.0263 | 6.61E-07 | 0.29 | 0.0028 | 0.0300 | 9.27E-01 | 24.77 |
| IL-36β | Squamous cell lung cancer | rs16880182 | C | T | 0.05237 | -0.2690 | 0.0565 | 1.95E-06 | 0.05 | 0.0254 | 0.0624 | 6.95E-01 | 22.67 |
| IL-36β | Squamous cell lung cancer | rs170187 | C | T | 0.74954 | -0.1327 | 0.0290 | 4.90E-06 | 0.76 | -0.0048 | 0.0318 | 8.83E-01 | 20.94 |
| IL-36β | Squamous cell lung cancer | rs184204067 | T | C | 0.01986 | 0.4742 | 0.0952 | 6.31E-07 | 0.04 | -0.0029 | 0.0735 | 9.71E-01 | 24.81 |
| IL-36β | Squamous cell lung cancer | rs188013949 | A | G | 0.01038 | 0.5876 | 0.1254 | 2.75E-06 | 0.01 | -0.0530 | 0.1359 | 7.52E-01 | 21.96 |
| IL-36γ | Lung cancer | rs117038998 | T | G | 0.05092 | 0.2608 | 0.0571 | 4.90E-06 | 0.05 | 0.0275 | 0.0504 | 5.95E-01 | 20.86 |
| IL-36γ | Lung cancer | rs13075826 | T | C | 0.0403 | 0.3400 | 0.0637 | 9.55E-08 | 0.04 | -0.1131 | 0.0385 | 1.20E-02 | 28.49 |
| IL-36γ | Lung cancer | rs13340573 | G | A | 0.08216 | 0.2238 | 0.0471 | 2.00E-06 | 0.08 | 0.0155 | 0.0349 | 6.64E-01 | 22.58 |
| IL-36γ | Lung cancer | rs1421799 | C | A | 0.14489 | 0.1645 | 0.0354 | 3.39E-06 | 0.16 | 0.0088 | 0.0252 | 7.32E-01 | 21.59 |
| IL-36γ | Lung cancer | rs17625605 | A | G | 0.04244 | -0.2852 | 0.0624 | 4.90E-06 | 0.04 | -0.0669 | 0.0433 | 1.68E-01 | 20.89 |
| IL-36γ | Lung adenocarcinoma | rs117038998 | T | G | 0.05092 | 0.2608 | 0.0571 | 4.90E-06 | 0.05 | 0.0286 | 0.0762 | 7.21E-01 | 20.86 |
| IL-36γ | Lung adenocarcinoma | rs13075826 | T | C | 0.0403 | 0.3400 | 0.0637 | 9.55E-08 | 0.04 | -0.1444 | 0.0549 | 3.30E-02 | 28.49 |
| IL-36γ | Lung adenocarcinoma | rs13340573 | G | A | 0.08216 | 0.2238 | 0.0471 | 2.00E-06 | 0.08 | 0.0999 | 0.0578 | 7.06E-02 | 22.58 |
| IL-36γ | Lung adenocarcinoma | rs1421799 | C | A | 0.14489 | 0.1645 | 0.0354 | 3.39E-06 | 0.16 | -0.0605 | 0.0360 | 1.28E-01 | 21.59 |
| IL-36γ | Lung adenocarcinoma | rs17625605 | A | G | 0.04244 | -0.2852 | 0.0624 | 4.90E-06 | 0.04 | -0.0480 | 0.0662 | 5.20E-01 | 20.89 |
| IL-36γ | Squamous cell lung cancer | rs117038998 | T | G | 0.05092 | 0.2608 | 0.0571 | 4.90E-06 | 0.05 | -0.0120 | 0.0730 | 8.80E-01 | 20.86 |
| IL-36γ | Squamous cell lung cancer | rs13075826 | T | C | 0.0403 | 0.3400 | 0.0637 | 9.55E-08 | 0.04 | -0.0198 | 0.0635 | 7.75E-01 | 28.49 |
| IL-36γ | Squamous cell lung cancer | rs13340573 | G | A | 0.08216 | 0.2238 | 0.0471 | 2.00E-06 | 0.08 | -0.0351 | 0.0511 | 5.29E-01 | 22.58 |
| IL-36γ | Squamous cell lung cancer | rs1421799 | C | A | 0.14489 | 0.1645 | 0.0354 | 3.39E-06 | 0.16 | 0.0698 | 0.0408 | 7.71E-02 | 21.59 |
| IL-36γ | Squamous cell lung cancer | rs17625605 | A | G | 0.04244 | -0.2852 | 0.0624 | 4.90E-06 | 0.04 | -0.0907 | 0.0651 | 2.37E-01 | 20.89 |
| IL-37 | Lung cancer | rs1955055 | G | A | 0.20173 | -0.1495 | 0.0323 | 3.63E-06 | 0.18 | -0.0088 | 0.0238 | 7.22E-01 | 21.42 |
| IL-37 | Lung cancer | rs2921184 | G | A | 0.87185 | -0.1781 | 0.0371 | 1.55E-06 | 0.88 | 0.0202 | 0.0260 | 4.58E-01 | 23.05 |
| IL-37 | Lung cancer | rs61191477 | A | C | 0.07086 | 0.2289 | 0.0490 | 2.95E-06 | 0.07 | 0.0980 | 0.0445 | 1.97E-02 | 21.82 |
| IL-37 | Lung cancer | rs76027472 | T | C | 0.07502 | -0.2330 | 0.0475 | 9.12E-07 | 0.07 | 0.0504 | 0.0418 | 2.23E-01 | 24.06 |
| IL-37 | Lung cancer | rs956142 | T | C | 0.42198 | -0.1214 | 0.0255 | 1.91E-06 | 0.43 | -0.0139 | 0.0181 | 4.57E-01 | 22.67 |
| IL-37 | Lung adenocarcinoma | rs1955055 | G | A | 0.20173 | -0.1495 | 0.0323 | 3.63E-06 | 0.18 | -0.0179 | 0.0366 | 6.44E-01 | 21.42 |
| IL-37 | Lung adenocarcinoma | rs2921184 | G | A | 0.87185 | -0.1781 | 0.0371 | 1.55E-06 | 0.88 | 0.0083 | 0.0408 | 8.46E-01 | 23.05 |
| IL-37 | Lung adenocarcinoma | rs61191477 | A | C | 0.07086 | 0.2289 | 0.0490 | 2.95E-06 | 0.07 | 0.0105 | 0.0635 | 8.75E-01 | 21.82 |
| IL-37 | Lung adenocarcinoma | rs76027472 | T | C | 0.07502 | -0.2330 | 0.0475 | 9.12E-07 | 0.07 | 0.1036 | 0.0667 | 1.05E-01 | 24.06 |
| IL-37 | Lung adenocarcinoma | rs956142 | T | C | 0.42198 | -0.1214 | 0.0255 | 1.91E-06 | 0.43 | -0.0032 | 0.0281 | 9.13E-01 | 22.67 |
| IL-37 | Squamous cell lung cancer | rs1955055 | G | A | 0.20173 | -0.1495 | 0.0323 | 3.63E-06 | 0.18 | -0.0057 | 0.0366 | 8.81E-01 | 21.42 |
| IL-37 | Squamous cell lung cancer | rs2921184 | G | A | 0.87185 | -0.1781 | 0.0371 | 1.55E-06 | 0.88 | 0.0291 | 0.0394 | 4.91E-01 | 23.05 |
| IL-37 | Squamous cell lung cancer | rs61191477 | A | C | 0.07086 | 0.2289 | 0.0490 | 2.95E-06 | 0.07 | 0.1015 | 0.0675 | 1.18E-01 | 21.82 |
| IL-37 | Squamous cell lung cancer | rs76027472 | T | C | 0.07502 | -0.2330 | 0.0475 | 9.12E-07 | 0.07 | 0.0907 | 0.0671 | 1.65E-01 | 24.06 |
| IL-37 | Squamous cell lung cancer | rs956142 | T | C | 0.42198 | -0.1214 | 0.0255 | 1.91E-06 | 0.43 | -0.0256 | 0.0275 | 3.79E-01 | 22.67 |

# **Table S4.** Excluded pQTLs that were ill-annotated in RegulomeDB database in terms of allele frequency in European descendants

| Biomarkers | SNP | chr_hg19 | po_hg19 | Effect allele | Other allele | beta | se | *P*-value | EAF | RefAllele | AltAllele | EUR | Gene |
| --- | --- | --- | --- | --- | --- | --- | --- | --- | --- | --- | --- | --- | --- |
| IL-1α | rs60010354 | 11 | 108513088 | G | A | 0.1142 | 0.0248 | 4.17E-06 | 0.41651 | A | G | N.A. | DDX10 |
| IL-18BP | rs200821392 | 10 | 5822184 | A | C | -0.1535 | 0.0318 | 1.35E-06 | 0.22748 | C | A | N.A. | GDI2 |
| IL-18Rα | rs9272100 | 6 | 32599814 | T | C | 0.1291 | 0.0281 | 4.47E-06 | 0.26481 | C | T | N.A. | HLA-DQA1 |
| IL-36α | rs9273410 | 6 | 32627250 | A | C | 0.1247 | 0.0253 | 8.51E-07 | 0.55591 | C | A | N.A. | HLA-DQB1 |
| IL-36β | rs199789755 | 5 | 128647865 | G | A | -0.1958 | 0.0426 | 4.27E-06 | 0.09865 | A | G | N.A. | MIR4460 |
| IL-36β | rs71430365 | 2 | 242530419 | G | A | -0.1467 | 0.0318 | 4.07E-06 | 0.21661 | A | G | N.A. | THAP4 |
| IL-36β | rs9345080 | 6 | 91758073 | T | A | -0.4483 | 0.0958 | 2.88E-06 | 0.01936 | A | T | N.A. | RP1-177I10.1 |
| IL-36γ | rs548416824 | 20 | 5979820 | C | A | -0.3782 | 0.0772 | 9.55E-07 | 0.0374 | N.A. | N.A. | N.A. | |
| IL-37 | rs2332686 | 17 | 54712453 | A | C | 0.1341 | 0.0288 | 3.31E-06 | 0.28776 | C | A | N.A. | NOG |

# **Table S5.** Excluded *tran*-pQLTs that were associated with expression quantitative trait loci (eQTLs) in RegulomeDB database

| Biomarkers | SNP | Gene | Expression QTL |
| --- | --- | --- | --- |
| IL-18 | rs385076 | NLRC4 | GTEx2015_v6,Cells_EBV-transformed_lymphocytes,NLRC4,3.52478617699342e-06;GTEx2015_v6,Cells_Transformed_fibroblasts,RP11-563N4.1,2.05448776644257e-06;GTEx2015_v6,Lung,NLRC4,3.45007791625485e-08;GTEx2015_v6,Muscle_Skeletal,SLC30A6,1.62356480906562e-08;Lappalainen2013,Lymphoblastoid_EUR_exonlevel,ENSG00000091106.14_32474676_32476670,1.16031774223387e-16;Lappalainen2013,Lymphoblastoid_EUR_genelevel,NLRC4,9.06070953258018e-14 |
| IL-18 | rs17229943 | RAD17 | GTEx2015_v6,Adipose_Subcutaneous,RP11-1319K7.1,5.12927654811016e-05 |
| IL-18 | rs1852105 | ZNF679 | GTEx2015_v6,Muscle_Skeletal,ZNF736,1.47323946585794e-06 |
| IL-18 | rs2729385 | SLC43A1 | GTEx2015_v6,Adipose_Subcutaneous,SMTNL1,4.206140370132e-13;GTEx2015_v6,Adipose_Visceral_Omentum,SMTNL1,2.39276824367756e-06;GTEx2015_v6,Adrenal_Gland,SMTNL1,2.38471170687107e-07;GTEx2015_v6,Artery_Aorta,SMTNL1,3.66451660844041e-11;GTEx2015_v6,Artery_Tibial,SMTNL1,9.57903096895986e-08;GTEx2015_v6,Brain_Cerebellum,SMTNL1,1.17499922250051e-06;GTEx2015_v6,Breast_Mammary_Tissue,SMTNL1,5.6410097827212e-07;GTEx2015_v6,Cells_EBV-transformed_lymphocytes,SMTNL1,6.01530957377943e-08;GTEx2015_v6,Cells_Transformed_fibroblasts,SMTNL1,6.92149501833806e-13;GTEx2015_v6,Colon_Sigmoid,SMTNL1,7.32073304357493e-06;GTEx2015_v6,Colon_Transverse,SMTNL1,8.03066919101303e-10;GTEx2015_v6,Esophagus_Gastroesophageal_Junction,SMTNL1,3.78741665865681e-07;GTEx2015_v6,Esophagus_Mucosa,SMTNL1,1.90257830494472e-06;GTEx2015_v6,Esophagus_Muscularis,SMTNL1,3.67592158105237e-13;GTEx2015_v6,Lung,SMTNL1,1.23012381063858e-14;GTEx2015_v6,Nerve_Tibial,SMTNL1,1.17611277251475e-09;GTEx2015_v6,Skin_Not_Sun_Exposed_Suprapubic,SMTNL1,1.23530056660372e-05;GTEx2015_v6,Skin_Sun_Exposed_Lower_leg,SMTNL1,5.41134143651669e-13;GTEx2015_v6,Small_Intestine_Terminal_Ileum,SMTNL1,3.64416287189789e-06;GTEx2015_v6,Spleen,SMTNL1,5.42638537398896e-08;GTEx2015_v6,Thyroid,SMTNL1,3.40898130925382e-14;GTEx2015_v6,Whole_Blood,SMTNL1,7.77915426187226e-07;Lappalainen2013,Lymphoblastoid_EUR_exonlevel,ENSG00000214872.4_57310114_57310847,7.16751540278696e-15;Lappalainen2013,Lymphoblastoid_EUR_genelevel,SMTNL1,6.44175079232949e-13;Westra2013,Whole_Blood,MED19,8.345185070076389E-20;Westra2013,Whole_Blood,TIMM10,1.9645714380919488E-185;Westra2013,Whole_Blood,UBE2L6,0.002294516723458952;Westra2013,Whole_Blood,UBE2L6,1.715388241770864E-11 |
| IL-18 | rs71478720 | AP002884.2 | GTEx2015_v6,Lung,IL18,1.36120783543032e-16;GTEx2015_v6,Muscle_Skeletal,IL18,2.68356985060617e-07;GTEx2015_v6,Pancreas,IL18,6.04578605874945e-12;GTEx2015_v6,Skin_Not_Sun_Exposed_Suprapubic,IL18,1.98103159143753e-09;GTEx2015_v6,Skin_Sun_Exposed_Lower_leg,IL18,3.09794353746608e-10;GTEx2015_v6,Thyroid,BCO2,3.58760951999846e-08;GTEx2015_v6,Thyroid,IL18,3.60355099022785e-06;GTEx2015_v6,Thyroid,RP11-356J5.12,9.01907970658234e-06 |
| IL-18 | rs1979967 | TMED3 | Westra2013,Whole_Blood,KIAA1024,4.827841259114473E-9 |
| IL-18 | rs10414578 | LILRB1 | GTEx2015_v6,Cells_EBV-transformed_lymphocytes,LILRB2,1.14621689972183e-12;GTEx2015_v6,Cells_EBV-transformed_lymphocytes,LILRB5,1.19972846004951e-08;GTEx2015_v6,Testis,LILRB1,1.07411304038705e-06;GTEx2015_v6,Whole_Blood,LILRA1,8.83581096750094e-07;Lappalainen2013,Lymphoblastoid_EUR_exonlevel,ENSG00000104972.10_55128612_55128803,1.02461434665338e-07;Lappalainen2013,Lymphoblastoid_EUR_exonlevel,ENSG00000104972.10_55141861_55142558,4.38230816992842e-07;Lappalainen2013,Lymphoblastoid_EUR_exonlevel,ENSG00000104972.10_55143386_55143688,2.58956391817483e-07;Lappalainen2013,Lymphoblastoid_EUR_exonlevel,ENSG00000104972.10_55146092_55146214,6.27225074777673e-14;Lappalainen2013,Lymphoblastoid_EUR_exonlevel,ENSG00000104972.10_55147945_55148103,5.98905529565514e-23;Lappalainen2013,Lymphoblastoid_EUR_exonlevel,ENSG00000104972.10_55148183_55148979,9.67531843315521e-07 |
| IL-18 | rs11700536 | AP001631.9 | Westra2013,Whole_Blood,CBS,9.469404246552941E-5 |
| IL-1β | rs115242021 | U1 | GTEx2015_v6,Skin_Sun_Exposed_Lower_leg,ANKRD35,3.81962827453163e-05;GTEx2015_v6,Thyroid,PDE4DIP,2.07133103437519e-05 |
| IL-18BP | rs11265492 | CD244 | Lappalainen2013,Lymphoblastoid_EUR_exonlevel,ENSG00000122223.8_160799950_160801217,4.06987074655214e-08;Lappalainen2013,Lymphoblastoid_EUR_exonlevel,ENSG00000122223.8_160802309_160802365,2.98964960004544e-06;Lappalainen2013,Lymphoblastoid_EUR_exonlevel,ENSG00000122223.8_160803841_160803906,8.8187456518926e-07;Lappalainen2013,Lymphoblastoid_EUR_exonlevel,ENSG00000122223.8_160805985_160806044,6.13506954443814e-08;Lappalainen2013,Lymphoblastoid_EUR_exonlevel,ENSG00000122223.8_160808241_160808308,1.76733796045819e-08;Lappalainen2013,Lymphoblastoid_EUR_exonlevel,ENSG00000122223.8_160808729_160808839,2.18969202198597e-07;Lappalainen2013,Lymphoblastoid_EUR_exonlevel,ENSG00000122223.8_160811000_160811290,8.69717909247338e-08;Lappalainen2013,Lymphoblastoid_EUR_exonlevel,ENSG00000122223.8_160811374_160811691,2.40832227171036e-07;Lappalainen2013,Lymphoblastoid_EUR_exonlevel,ENSG00000122223.8_160832407_160832692,3.06586020438023e-07;Lappalainen2013,Lymphoblastoid_EUR_genelevel,CD244,5.9937791037272e-07;Westra2013,Whole_Blood,LY9,1.586103850243671E-101;Westra2013,Whole_Blood,LY9,2.8468905014730323E-133 |
| IL-18BP | rs73402996 | TMEM130 | GTEx2015_v6,Artery_Aorta,NPTX2,3.64281867817494e-11 |
| IL-18Rα | rs13008334 | SLC9A4 | Westra2013,Whole_Blood,IL18R1,8.89314655231913E-8;Westra2013,Whole_Blood,IL18RAP,3.406181210567697E-76;Westra2013,Whole_Blood,MFSD9,7.014305741568262E-5 |
| IL-18Rα | rs58445015 | TMEM130 | GTEx2015_v6,Artery_Aorta,NPTX2,5.96839661333206e-10 |
| IL-18Rα | rs1420106 | IL18RAP | GTEx2015_v6,Brain_Hippocampus,IL18R1,1.3948011247318e-06;GTEx2015_v6,Lung,AC007278.3,5.71648379032845e-06;GTEx2015_v6,Lung,IL18R1,1.5632022162979e-06;GTEx2015_v6,Lung,IL1RL1,6.50313048677816e-07;GTEx2015_v6,Muscle_Skeletal,IL18R1,2.05106241919337e-06;GTEx2015_v6,Skin_Sun_Exposed_Lower_leg,IL18R1,1.51986920546878e-08;GTEx2015_v6,Testis,SLC9A4,1.99304175734754e-06;GTEx2015_v6,Whole_Blood,AC007278.2,1.64592686496617e-12;GTEx2015_v6,Whole_Blood,AC007278.3,4.66487491125886e-14;GTEx2015_v6,Whole_Blood,IL18R1,4.81457425619133e-06;GTEx2015_v6,Whole_Blood,IL18RAP,7.41837183269319e-31;Westra2013,Whole_Blood,IL18R1,2.4896343429660337E-33;Westra2013,Whole_Blood,IL18RAP,9.813427854297537E-198 |
| IL-18Rα | rs13014644 | IL18R1 | GTEx2015_v6,Lung,AC007278.3,1.3339677125094e-05;GTEx2015_v6,Whole_Blood,AC007278.3,7.89857188886403e-06 |
| IL-18Rα | rs78123896 | IL18RAP | GTEx2015_v6,Muscle_Skeletal,IL18R1,8.01700453073711e-06;GTEx2015_v6,Whole_Blood,AC007278.3,7.14171253329553e-07 |
| IL-18Rα | rs10167431 | IL1RL2 | GTEx2015_v6,Skin_Not_Sun_Exposed_Suprapubic,IL1RL2,1.10466306622446e-06;GTEx2015_v6,Skin_Sun_Exposed_Lower_leg,IL1RL2,1.43653786631263e-07;GTEx2015_v6,Thyroid,IL1RL2,9.23478852787835e-10;Westra2013,Whole_Blood,IL18RAP,7.485121900894718E-11 |
| IL-18Rα | rs4140836 | SLC9A2 | Westra2013,Whole_Blood,IL18RAP,3.205390190949254E-6 |
| IL-1R1 | rs12493830 | ST3GAL6-AS1 | GTEx2015_v6,Adipose_Subcutaneous,ST3GAL6-AS1,1.97105642749949e-16;GTEx2015_v6,Adipose_Visceral_Omentum,ST3GAL6-AS1,6.32195043027876e-11;GTEx2015_v6,Adrenal_Gland,ST3GAL6-AS1,7.97900124755289e-07;GTEx2015_v6,Artery_Aorta,ST3GAL6-AS1,5.48757850863665e-12;GTEx2015_v6,Artery_Coronary,ST3GAL6-AS1,6.1962327517805e-10;GTEx2015_v6,Artery_Tibial,ST3GAL6,1.13275199287762e-05;GTEx2015_v6,Artery_Tibial,ST3GAL6-AS1,3.83288386701907e-11;GTEx2015_v6,Brain_Anterior_cingulate_cortex_BA24,ST3GAL6-AS1,1.11077909071971e-10;GTEx2015_v6,Brain_Caudate_basal_ganglia,ST3GAL6-AS1,2.41194536741699e-09;GTEx2015_v6,Brain_Cerebellar_Hemisphere,ST3GAL6-AS1,1.04591578768766e-09;GTEx2015_v6,Brain_Cerebellum,ST3GAL6-AS1,7.84982154970915e-08;GTEx2015_v6,Brain_Cortex,ST3GAL6-AS1,2.8840693955692e-09;GTEx2015_v6,Brain_Frontal_Cortex_BA9,ST3GAL6-AS1,1.62103050317129e-10;GTEx2015_v6,Brain_Hippocampus,ST3GAL6-AS1,7.11844867974726e-07;GTEx2015_v6,Brain_Hypothalamus,ST3GAL6-AS1,1.28535324390055e-08;GTEx2015_v6,Brain_Putamen_basal_ganglia,ST3GAL6-AS1,1.52004975473123e-07;GTEx2015_v6,Breast_Mammary_Tissue,ST3GAL6-AS1,2.73313652304005e-13;GTEx2015_v6,Cells_EBV-transformed_lymphocytes,ST3GAL6-AS1,5.85747897380411e-06;GTEx2015_v6,Cells_Transformed_fibroblasts,CPOX,2.62672170047362e-06;GTEx2015_v6,Cells_Transformed_fibroblasts,ST3GAL6,4.03773979978091e-13;GTEx2015_v6,Cells_Transformed_fibroblasts,ST3GAL6-AS1,5.51946730600281e-12;GTEx2015_v6,Colon_Sigmoid,ST3GAL6-AS1,4.45537210074347e-14;GTEx2015_v6,Esophagus_Gastroesophageal_Junction,ST3GAL6-AS1,1.07339347572694e-09;GTEx2015_v6,Esophagus_Mucosa,ST3GAL6,2.94368484773451e-13;GTEx2015_v6,Esophagus_Mucosa,ST3GAL6-AS1,2.6307100043381e-12;GTEx2015_v6,Esophagus_Muscularis,ST3GAL6-AS1,1.23392525650639e-13;GTEx2015_v6,Heart_Atrial_Appendage,ST3GAL6-AS1,2.30191203799069e-24;GTEx2015_v6,Heart_Left_Ventricle,ST3GAL6-AS1,5.10414856522815e-20;GTEx2015_v6,Liver,ST3GAL6-AS1,1.74970624660889e-07;GTEx2015_v6,Lung,ST3GAL6-AS1,6.15087009921118e-09;GTEx2015_v6,Muscle_Skeletal,ST3GAL6-AS1,5.67694930501777e-15;GTEx2015_v6,Nerve_Tibial,ST3GAL6-AS1,2.51034315817959e-18;GTEx2015_v6,Pancreas,ST3GAL6-AS1,6.29027948632179e-07;GTEx2015_v6,Skin_Not_Sun_Exposed_Suprapubic,ST3GAL6-AS1,3.48488126535269e-07;GTEx2015_v6,Skin_Sun_Exposed_Lower_leg,ST3GAL6-AS1,1.78651335513757e-13;GTEx2015_v6,Testis,ST3GAL6-AS1,2.18132516846256e-06;GTEx2015_v6,Thyroid,DCBLD2,5.52471484922727e-06;GTEx2015_v6,Thyroid,ST3GAL6,2.9531247016318e-06;GTEx2015_v6,Thyroid,ST3GAL6-AS1,3.33573611416865e-16 |
| IL-1R1 | rs11888059 | IL1R1 | Westra2013,Whole_Blood,IL1R2,0.0012935811232900004;Westra2013,Whole_Blood,IL1R2,7.262498373801614E-13 |
| IL-1R1 | rs4690014 | RGS12 | Hao2012,Lung,RGS12,8.50E-06 |
| IL-1RA | rs12121840 | RP11-280O1.2 | GTEx2015_v6,Esophagus_Muscularis,LRRC52,3.09675525662452e-08;GTEx2015_v6,Lung,LRRC52,4.34868960863599e-06;GTEx2015_v6,Stomach,LRRC52,1.26740912250005e-09;GTEx2015_v6,Stomach,RP11-280O1.2,2.57011916421612e-07 |
| IL-1Racp | rs1988743 | IL1RAP | Westra2013,Whole_Blood,IL1RAP\|AC108747.5-1,3.559906650111699E-8 |
| IL-1Racp | rs6444435 | IL1RAP | Westra2013,Whole_Blood,IL1RAP\|AC108747.5-1,4.561642351034914E-6 |
| IL-1Racp | rs1024949 | IL1RAP | Fairfax2014,Monocytes_IFN,PUM2,7.34E-10 |
| IL-1Racp | rs13084868 | GMNC | GTEx2015_v6,Heart_Left_Ventricle,GMNC,9.14264275471493e-07 |
| IL-1Racp | rs4815878 | SRXN1 | Lappalainen2013,Lymphoblastoid_EUR_exonlevel,ENSG00000172070.5_627259_629561,7.33039162545634e-06;Lappalainen2013,Lymphoblastoid_EUR_exonlevel,ENSG00000234516.1_628647_629157,7.46622535222143e-06 |
| IL-1Racp | rs5753690 | SFI1 | GTEx2015_v6,Pituitary,SFI1,2.44989135154641e-07;Lappalainen2013,Lymphoblastoid_EUR_exonlevel,ENSG00000198089.9_31998198_31998279,8.01792088704581e-06;Lappalainen2013,Lymphoblastoid_EUR_exonlevel,ENSG00000198089.9_31998593_31998771,2.88520604804765e-08;Lappalainen2013,Lymphoblastoid_EUR_exonlevel,ENSG00000198089.9_31999730_31999805,5.3352074517302e-07;Lappalainen2013,Lymphoblastoid_EUR_exonlevel,ENSG00000198089.9_32000295_32000390,8.37661804920748e-06;Lappalainen2013,Lymphoblastoid_EUR_exonlevel,ENSG00000198089.9_32000855_32000931,7.33929083643046e-07;Lappalainen2013,Lymphoblastoid_EUR_exonlevel,ENSG00000198089.9_32003923_32004019,1.76214801093622e-06;Lappalainen2013,Lymphoblastoid_EUR_exonlevel,ENSG00000198089.9_32007129_32007289,5.61648627630726e-07 |
| IL-1Racp | rs13059920 | IL1RAP | Westra2013,Whole_Blood,IL1RAP\|AC108747.5-1,3.765440759033298E-4 |
| IL-1α | rs2749675 | ZDHHC14 | GTEx2015_v6,Artery_Tibial,RP3-395C13.1,2.26131794162987e-06 |
| IL-36α | rs704 | SEBOX | GTEx2015_v6,Adipose_Subcutaneous,CTD-2350C19.1,2.32352362079435e-05;GTEx2015_v6,Adipose_Subcutaneous,TMEM199,7.84567907215868e-06;GTEx2015_v6,Adipose_Visceral_Omentum,CTB-96E2.3,1.014552077118e-05;GTEx2015_v6,Brain_Anterior_cingulate_cortex_BA24,TMEM199,1.42483173207507e-06;GTEx2015_v6,Brain_Nucleus_accumbens_basal_ganglia,POLDIP2,1.39205901813054e-06;GTEx2015_v6,Esophagus_Muscularis,TMEM199,5.92521360059319e-06;GTEx2015_v6,Lung,CTB-96E2.3,3.55553556447343e-07;GTEx2015_v6,Nerve_Tibial,POLDIP2,1.72547901581911e-05;GTEx2015_v6,Nerve_Tibial,TMEM199,6.12926870569311e-09;GTEx2015_v6,Skin_Not_Sun_Exposed_Suprapubic,CTB-96E2.3,6.52400485669231e-06;GTEx2015_v6,Skin_Sun_Exposed_Lower_leg,TMEM199,3.80090143907547e-06;Lappalainen2013,Lymphoblastoid_EUR_exonlevel,ENSG00000109084.8_26646161_26646391,3.48134951358563e-06;Lappalainen2013,Lymphoblastoid_EUR_exonlevel,ENSG00000109084.8_26652529_26652673,3.71777094549573e-09;Lappalainen2013,Lymphoblastoid_EUR_exonlevel,ENSG00000109084.8_26653560_26655711,3.71214844291925e-11;Lappalainen2013,Lymphoblastoid_EUR_exonlevel,ENSG00000244045.3_26685939_26686012,8.80034639902315e-06;Lappalainen2013,Lymphoblastoid_EUR_exonlevel,ENSG00000244045.3_26686338_26686427,2.76662859551715e-06;Lappalainen2013,Lymphoblastoid_EUR_exonlevel,ENSG00000244045.3_26687552_26687594,9.22219429769034e-08;Lappalainen2013,Lymphoblastoid_EUR_exonlevel,ENSG00000244045.3_26687758_26687870,9.42193841019436e-12;Lappalainen2013,Lymphoblastoid_EUR_genelevel,TMEM97,3.35219063860687e-11;Westra2013,Whole_Blood,C17orf32,1.340653240550376E-7;Westra2013,Whole_Blood,POLDIP2,3.3476088186803387E-4;Westra2013,Whole_Blood,SARM1\|SLC46A1,6.650300176883694E-37;Westra2013,Whole_Blood,TMEM97,1.0879911283915114E-51 |
| IL-36α | rs4239214 | SCARNA20 | Lappalainen2013,Lymphoblastoid_EUR_exonlevel,ENSG00000160606.5_27051366_27051911,1.50964876657378e-05 |
| IL-36α | rs62065286 | SLC46A1 | Lappalainen2013,Lymphoblastoid_EUR_exonlevel,ENSG00000109084.8_26646161_26646391,1.60437421692752e-05;Lappalainen2013,Lymphoblastoid_EUR_exonlevel,ENSG00000109084.8_26652529_26652673,1.50802348713861e-07;Lappalainen2013,Lymphoblastoid_EUR_exonlevel,ENSG00000109084.8_26653560_26655711,4.36695065837435e-07;Lappalainen2013,Lymphoblastoid_EUR_genelevel,TMEM97,6.73006128996424e-07 |
| IL-36α | rs74480769 | C7 | GTEx2015_v6,Artery_Tibial,CARD6,2.69036027207574e-06 |
| IL-36α | rs10922094 | CFH | GTEx2015_v6,Liver,CFHR1,1.24205085579019e-07;GTEx2015_v6,Nerve_Tibial,CFH,1.64304577663586e-05;Westra2013,Whole_Blood,CFH,1.1503047024430609E-5;Westra2013,Whole_Blood,CFH,8.28778998644239E-4 |
| IL-36α | rs749331 | KLHL29 | GTEx2015_v6,Nerve_Tibial,RP11-507M3.1,9.6033407940254e-06;GTEx2015_v6,Whole_Blood,UBXN2A,1.03373412206706e-06 |
| IL-36α | rs12194933 | FNDC1 | GTEx2015_v6,Esophagus_Muscularis,FNDC1,4.65574935413495e-07 |
| IL-36β | rs1026519 | MBP | Westra2013,Whole_Blood,MBP,0.0026159194914398046 |
| IL-36β | rs56208304 | SH3TC1 | Lappalainen2013,Lymphoblastoid_EUR_exonlevel,ENSG00000125089.10_8217838_8217984,1.61626085522094e-06 |
| IL-37 | rs74480769 | C7 | GTEx2015_v6,Artery_Tibial,CARD6,2.69036027207574e-06 |
| IL-37 | rs11259827 | RP11-292F22.6 | GTEx2015_v6,Nerve_Tibial,SYT15,2.68953321339702e-05 |
| IL-37 | rs6981787 | U6 | GTEx2015_v6,Cells_Transformed_fibroblasts,ERI1,4.5471886307061e-06;GTEx2015_v6,Cells_Transformed_fibroblasts,FAM86B3P,2.56473018267627e-05 |
| IL-37 | rs7539005 | CFH | GTEx2015_v6,Liver,CFHR1,7.56238874199553e-08 |

# **Table S6.** Excluded *trans*-pQTLs that were associated with expression quantitative trait loci (eQTLs) in PhenoScanner database

| Biomarkers | snp | trait | pmid | ancestry | tissue | Expression gene |
| --- | --- | --- | --- | --- | --- | --- |
| IL-18 | rs385076 | Exon expression | 27918533 | European | Whole blood | NLRC4 |
| IL-18 | rs385076 | Gene expression | 27918533 | European | Whole blood | YIPF4 |
| IL-18 | rs385076 | Gene expression | 27863251 | European | Monocytes | NLRC4 |
| IL-18 | rs385076 | Exon expression | 24037378 | European | Lymphoblastoid cell lines | NLRC4 |
| IL-18 | rs385076 | Gene expression | eQTLGen | European | Whole blood | NLRC4 |
| IL-18 | rs385076 | Gene expression | eQTLGen | European | Whole blood | YIPF4 |
| IL-18 | rs385076 | Gene expression | 25720628 | European | Liver | NLRC4 |
| IL-18 | rs385076 | Gene expression | 25720628 | European | Atherosclerotic internal thoracic artery | NLRC4 |
| IL-18 | rs1656939 | Gene expression | eQTLGen | European | Whole blood | RFC4 |
| IL-18 | rs116656892 | Gene expression | eQTLGen | European | Whole blood | CCDC125 |
| IL-18 | rs17229943 | Gene expression | eQTLGen | European | Whole blood | RP11-1319K7.1 |
| IL-18 | rs1852105 | Gene expression | 27918533 | European | Whole blood | TRIM60P18 |
| IL-18 | rs1852105 | Gene expression | eQTLGen | European | Whole blood | ERV3-1 |
| IL-18 | rs1852105 | Gene expression | eQTLGen | European | Whole blood | TRIM60P18 |
| IL-18 | rs1852105 | Gene expression | eQTLGen | European | Whole blood | ZNF680 |
| IL-18 | rs1852105 | Gene expression | eQTLGen | European | Whole blood | RP11-460N20.5 |
| IL-18 | rs1852105 | Gene expression | eQTLGen | European | Whole blood | ZNF736 |
| IL-18 | rs1852105 | Gene expression | eQTLGen | European | Whole blood | CCT6P3 |
| IL-18 | rs2729385 | Gene expression | 27918533 | European | Whole blood | SMTNL1 |
| IL-18 | rs2729385 | Gene expression | 27918533 | European | Whole blood | TIMM10 |
| IL-18 | rs2729385 | Gene expression | 27918533 | European | Whole blood | SLC43A1 |
| IL-18 | rs2729385 | Gene expression | 27918533 | European | Whole blood | UBE2L6 |
| IL-18 | rs2729385 | Gene expression | 27863251 | European | Neutrophils | TIMM10 |
| IL-18 | rs2729385 | Gene expression | 21829388 | European | Blood | TIMM10 |
| IL-18 | rs2729385 | Exon expression | 24037378 | European | Lymphoblastoid cell lines | SMTNL1 |
| IL-18 | rs2729385 | Gene expression | 24037378 | European | Lymphoblastoid cell lines | SMTNL1 |
| IL-18 | rs2729385 | Gene expression | 22941192 | European | Adipose subcutaneous | TIMM10 |
| IL-18 | rs2729385 | Gene expression | 22941192 | European | Lymphoblastoid cell lines | TIMM10 |
| IL-18 | rs2729385 | Gene expression | 22941192 | European | Skin | TIMM10 |
| IL-18 | rs2729385 | Gene expression | 24013639 | European | Peripheral blood | TIMM10 |
| IL-18 | rs2729385 | Gene expression | 24013639 | European | Peripheral blood | MED19 |
| IL-18 | rs2729385 | Gene expression | 24013639 | European | Peripheral blood | UBE2L6 |
| IL-18 | rs2729385 | Gene expression | eQTLGen | European | Whole blood | TIMM10 |
| IL-18 | rs2729385 | Gene expression | eQTLGen | European | Whole blood | SMTNL1 |
| IL-18 | rs2729385 | Gene expression | eQTLGen | European | Whole blood | MED19 |
| IL-18 | rs2729385 | Gene expression | eQTLGen | European | Whole blood | UBE2L6 |
| IL-18 | rs2729385 | Gene expression | eQTLGen | European | Whole blood | SLC43A1 |
| IL-18 | rs2729385 | Gene expression | 22446964 | European | B cells | TIMM10 |
| IL-18 | rs2729385 | Gene expression | 22446964 | European | Monocytes | TIMM10 |
| IL-18 | rs2729385 | Gene expression | 24604202 | European | Blood | TIMM10 |
| IL-18 | rs71478720 | Gene expression | 27918533 | European | Whole blood | IL18 |
| IL-18 | rs71478720 | Gene expression | 27863251 | European | Neutrophils | IL18 |
| IL-18 | rs71478720 | Gene expression | eQTLGen | European | Whole blood | IL18 |
| IL-18 | rs71478720 | Gene expression | eQTLGen | European | Whole blood | RP11-356J5.12 |
| IL-18 | rs117903455 | Gene expression | eQTLGen | European | Whole blood | IL18 |
| IL-18 | rs1979967 | Gene expression | 27918533 | European | Whole blood | KIAA1024 |
| IL-18 | rs1979967 | Gene expression | 24013639 | European | Peripheral blood | KIAA1024 |
| IL-18 | rs1979967 | Gene expression | eQTLGen | European | Whole blood | KIAA1024 |
| IL-18 | rs10414578 | Gene expression | 27918533 | European | Whole blood | LILRA1 |
| IL-18 | rs10414578 | Gene expression | 27918533 | European | Whole blood | LILRB1;AC009892.10 |
| IL-18 | rs10414578 | Gene expression | 27918533 | European | Whole blood | AC009892.9 |
| IL-18 | rs10414578 | Gene expression | 27918533 | European | Whole blood | LILRB4;VN1R105P |
| IL-18 | rs10414578 | Gene expression | 27918533 | European | Whole blood | AC011515.2 |
| IL-18 | rs10414578 | Gene expression | 27918533 | European | Whole blood | CTB-83J4.2 |
| IL-18 | rs10414578 | Gene expression | 27918533 | European | Whole blood | LILRA2 |
| IL-18 | rs10414578 | Exon expression | 24037378 | European | Lymphoblastoid cell lines | LILRB1 |
| IL-18 | rs10414578 | Exon expression | 24037378 | European | Lymphoblastoid cell lines | LILRB1 |
| IL-18 | rs10414578 | Gene expression | eQTLGen | European | Whole blood | LILRA1 |
| IL-18 | rs10414578 | Gene expression | eQTLGen | European | Whole blood | LILRB1 |
| IL-18 | rs10414578 | Gene expression | eQTLGen | European | Whole blood | LILRB4 |
| IL-18 | rs10414578 | Gene expression | eQTLGen | European | Whole blood | LILRA2 |
| IL-18 | rs10414578 | Gene expression | eQTLGen | European | Whole blood | CTB-83J4.2 |
| IL-18 | rs11700536 | Gene expression | 27918533 | European | Whole blood | U2AF1 |
| IL-18 | rs11700536 | Gene expression | eQTLGen | European | Whole blood | U2AF1 |
| IL-18 | rs11700536 | Gene expression | eQTLGen | European | Whole blood | CBS |
| IL-1β | rs115242021 | Gene expression | 27918533 | European | Whole blood | ANKRD35 |
| IL-1β | rs115242021 | Gene expression | eQTLGen | European | Whole blood | ANKRD35 |
| IL-1β | rs143319329 | Gene expression | eQTLGen | European | Whole blood | RP11-274B21.4 |
| IL-1β | rs143319329 | Gene expression | eQTLGen | European | Whole blood | RP11-274B21.3 |
| IL-1β | rs143319329 | Gene expression | eQTLGen | European | Whole blood | RP11-274B21.2 |
| IL-1β | rs9898641 | Gene expression | eQTLGen | European | Whole blood | HEATR6 |
| IL-1β | rs9898641 | Gene expression | eQTLGen | European | Whole blood | TUBD1 |
| IL-1Ra | rs12121840 | Gene expression | 27918533 | European | Whole blood | MGST3 |
| IL-1Ra | rs12121840 | Gene expression | eQTLGen | European | Whole blood | MGST3 |
| IL-1Ra | rs12121840 | Gene expression | eQTLGen | European | Whole blood | ALDH9A1 |
| IL-1Ra | rs56134659 | Gene expression | eQTLGen | European | Whole blood | COPG1 |
| IL-1Ra | rs56134659 | Gene expression | eQTLGen | European | Whole blood | H1FX |
| IL-1Ra | rs187166731 | Gene expression | eQTLGen | European | Whole blood | C22orf32 |
| IL-1Ra | rs187166731 | Gene expression | eQTLGen | European | Whole blood | MEI1 |
| IL-1Ra | rs187166731 | Gene expression | eQTLGen | European | Whole blood | NAGA |
| IL-1Ra | rs9623661 | Gene expression | 27918533 | European | Whole blood | A4GALT |
| IL-1Ra | rs9623661 | Gene expression | eQTLGen | European | Whole blood | A4GALT |
| IL-1Ra | rs9623661 | Gene expression | eQTLGen | European | Whole blood | RRP7B |
| IL-18BP | rs11265492 | Exon expression | 24037378 | European | Lymphoblastoid cell lines | CD244 |
| IL-18BP | rs11265492 | Exon expression | 24037378 | European | Lymphoblastoid cell lines | CD244 |
| IL-18BP | rs11265492 | Gene expression | 24013639 | European | Peripheral blood | LY9 |
| IL-18BP | rs11265492 | Gene expression | 24013639 | European | Peripheral blood | LY9 |
| IL-18BP | rs11265492 | Gene expression | eQTLGen | European | Whole blood | LY9 |
| IL-18BP | rs11265492 | Gene expression | eQTLGen | European | Whole blood | CD244 |
| IL-18BP | rs11265492 | Gene expression | eQTLGen | European | Whole blood | ITLN1 |
| IL-18BP | rs11265492 | Gene expression | eQTLGen | European | Whole blood | F11R |
| IL-18BP | rs11203320 | Gene expression | 27918533 | European | Whole blood | PADI2 |
| IL-18BP | rs11203320 | Gene expression | eQTLGen | European | Whole blood | PADI2 |
| IL-18BP | rs11203320 | Gene expression | eQTLGen | European | Whole blood | NECAP2 |
| IL-18BP | rs4806509 | Gene expression | 27918533 | European | Whole blood | NLRP12 |
| IL-18BP | rs4806509 | Gene expression | eQTLGen | European | Whole blood | NLRP12 |
| IL-18BP | rs73402996 | Gene expression | eQTLGen | European | Whole blood | AC004893.10 |
| IL-18BP | rs73402996 | Gene expression | eQTLGen | European | Whole blood | AC004893.11 |
| IL-18BP | rs6139149 | Gene expression | eQTLGen | European | Whole blood | ITPA |
| IL-18Rα | rs1801689 | Gene expression | eQTLGen | European | Whole blood | PRKCA |
| IL-18Rα | rs13014004 | Gene expression | 27918533 | European | Whole blood | AC007278.3 |
| IL-18Rα | rs13014004 | Gene expression | 27918533 | European | Whole blood | IL18RAP |
| IL-18Rα | rs13014004 | Gene expression | 27918533 | European | Whole blood | IL18R1 |
| IL-18Rα | rs13014004 | Gene expression | 27918533 | European | Whole blood | AC007278.2 |
| IL-18Rα | rs13014004 | Gene expression | 27918533 | European | Whole blood | MIR4772 |
| IL-18Rα | rs13014004 | Gene expression | 27863251 | European | Neutrophils | IL18RAP |
| IL-18Rα | rs13014004 | Gene expression | 27863251 | European | Neutrophils | MIR4772 |
| IL-18Rα | rs13014004 | Gene expression | eQTLGen | European | Whole blood | IL18RAP |
| IL-18Rα | rs13014004 | Gene expression | eQTLGen | European | Whole blood | IL18R1 |
| IL-18Rα | rs13014004 | Gene expression | eQTLGen | European | Whole blood | AC007278.3 |
| IL-18Rα | rs13014004 | Gene expression | eQTLGen | European | Whole blood | AC007278.2 |
| IL-18Rα | rs13014004 | Gene expression | eQTLGen | European | Whole blood | IL1R1 |
| IL-18Rα | rs13014004 | Gene expression | eQTLGen | European | Whole blood | MIR4772 |
| IL-18Rα | rs13008334 | Gene expression | 27918533 | European | Whole blood | AC007278.3 |
| IL-18Rα | rs13008334 | Gene expression | 27918533 | European | Whole blood | IL18RAP |
| IL-18Rα | rs13008334 | Gene expression | 27918533 | European | Whole blood | MFSD9 |
| IL-18Rα | rs13008334 | Gene expression | 27918533 | European | Whole blood | AC007278.2 |
| IL-18Rα | rs13008334 | Gene expression | 24013639 | European | Peripheral blood | IL18RAP |
| IL-18Rα | rs13008334 | Gene expression | eQTLGen | European | Whole blood | IL18RAP |
| IL-18Rα | rs13008334 | Gene expression | eQTLGen | European | Whole blood | AC007278.3 |
| IL-18Rα | rs13008334 | Gene expression | eQTLGen | European | Whole blood | MFSD9 |
| IL-18Rα | rs13008334 | Gene expression | eQTLGen | European | Whole blood | IL18R1 |
| IL-18Rα | rs13008334 | Gene expression | eQTLGen | European | Whole blood | AC007278.2 |
| IL-18Rα | rs13008334 | Gene expression | eQTLGen | European | Whole blood | IL1RL1 |
| IL-18Rα | rs141601545 | Gene expression | eQTLGen | European | Whole blood | IL18RAP |
| IL-18Rα | rs141601545 | Gene expression | eQTLGen | European | Whole blood | IL1RL1 |
| IL-18Rα | rs58445015 | Gene expression | eQTLGen | European | Whole blood | AC004893.10 |
| IL-18Rα | rs58445015 | Gene expression | eQTLGen | European | Whole blood | AC004893.11 |
| IL-18Rα | rs1420106 | Gene expression | 27918533 | European | Whole blood | IL18RAP |
| IL-18Rα | rs1420106 | Gene expression | 27918533 | European | Whole blood | AC007278.2 |
| IL-18Rα | rs1420106 | Gene expression | 27918533 | European | Whole blood | AC007278.3 |
| IL-18Rα | rs1420106 | Gene expression | 27918533 | European | Whole blood | IL1RL1 |
| IL-18Rα | rs1420106 | Gene expression | 27918533 | European | Whole blood | IL18R1 |
| IL-18Rα | rs1420106 | Gene expression | 27918533 | European | Whole blood | MIR4772 |
| IL-18Rα | rs1420106 | Gene expression | 27863251 | European | Neutrophils | IL18RAP |
| IL-18Rα | rs1420106 | Gene expression | 27863251 | European | Neutrophils | - |
| IL-18Rα | rs1420106 | Gene expression | 27863251 | European | Neutrophils | - |
| IL-18Rα | rs1420106 | Gene expression | 27863251 | European | Neutrophils | MIR4772 |
| IL-18Rα | rs1420106 | Gene expression | 22692066 | European | Whole blood | IL18RAP |
| IL-18Rα | rs1420106 | Gene expression | 24013639 | European | Peripheral blood | IL18RAP |
| IL-18Rα | rs1420106 | Gene expression | 24013639 | European | Peripheral blood | IL18R1 |
| IL-18Rα | rs1420106 | Gene expression | eQTLGen | European | Whole blood | IL18RAP |
| IL-18Rα | rs1420106 | Gene expression | eQTLGen | European | Whole blood | AC007278.2 |
| IL-18Rα | rs1420106 | Gene expression | eQTLGen | European | Whole blood | AC007278.3 |
| IL-18Rα | rs1420106 | Gene expression | eQTLGen | European | Whole blood | IL18R1 |
| IL-18Rα | rs1420106 | Gene expression | eQTLGen | European | Whole blood | IL1RL1 |
| IL-18Rα | rs1420106 | Gene expression | eQTLGen | European | Whole blood | MIR4772 |
| IL-18Rα | rs75968506 | Gene expression | eQTLGen | European | Whole blood | IL18RAP |
| IL-18Rα | rs149943125 | Gene expression | eQTLGen | European | Whole blood | IL18RAP |
| IL-18Rα | rs151254748 | Gene expression | eQTLGen | European | Whole blood | IL18RAP |
| IL-18Rα | rs151254748 | Gene expression | eQTLGen | European | Whole blood | IL18R1 |
| IL-18Rα | rs10935033 | Gene expression | eQTLGen | European | Whole blood | ACPP |
| IL-18Rα | rs10935033 | Gene expression | eQTLGen | European | Whole blood | NPHP3 |
| IL-18Rα | rs13014644 | Exon expression | 27918533 | European | Whole blood | IL18R1 |
| IL-18Rα | rs13014644 | Gene expression | 27918533 | European | Whole blood | IL18RAP |
| IL-18Rα | rs13014644 | Gene expression | 27918533 | European | Whole blood | AC007278.3 |
| IL-18Rα | rs13014644 | Gene expression | 27918533 | European | Whole blood | IL18R1 |
| IL-18Rα | rs13014644 | Gene expression | 27918533 | European | Whole blood | AC007278.2 |
| IL-18Rα | rs13014644 | Gene expression | 27863251 | European | Neutrophils | IL18R1 |
| IL-18Rα | rs13014644 | Gene expression | 27863251 | European | Neutrophils | IL18RAP |
| IL-18Rα | rs13014644 | Gene expression | 27863251 | European | Neutrophils | - |
| IL-18Rα | rs13014644 | Gene expression | 27863251 | European | Neutrophils | - |
| IL-18Rα | rs13014644 | Gene expression | 27863251 | European | Neutrophils | MIR4772 |
| IL-18Rα | rs13014644 | Gene expression | eQTLGen | European | Whole blood | IL18RAP |
| IL-18Rα | rs13014644 | Gene expression | eQTLGen | European | Whole blood | IL18R1 |
| IL-18Rα | rs13014644 | Gene expression | eQTLGen | European | Whole blood | AC007278.3 |
| IL-18Rα | rs13014644 | Gene expression | eQTLGen | European | Whole blood | AC007278.2 |
| IL-18Rα | rs13014644 | Gene expression | eQTLGen | European | Whole blood | MIR4772 |
| IL-18Rα | rs78123896 | Gene expression | 27918533 | European | Whole blood | AC007278.3 |
| IL-18Rα | rs78123896 | Gene expression | 27918533 | European | Whole blood | IL18R1 |
| IL-18Rα | rs78123896 | Gene expression | 27918533 | European | Whole blood | IL18RAP |
| IL-18Rα | rs78123896 | Gene expression | 27918533 | European | Whole blood | AC007278.2 |
| IL-18Rα | rs78123896 | Gene expression | eQTLGen | European | Whole blood | IL18RAP |
| IL-18Rα | rs78123896 | Gene expression | eQTLGen | European | Whole blood | IL18R1 |
| IL-18Rα | rs78123896 | Gene expression | eQTLGen | European | Whole blood | AC007278.3 |
| IL-18Rα | rs78123896 | Gene expression | eQTLGen | European | Whole blood | AC007278.2 |
| IL-18Rα | rs78123896 | Gene expression | eQTLGen | European | Whole blood | MIR4772 |
| IL-18Rα | rs10167431 | Gene expression | 27918533 | European | Whole blood | AC007278.3 |
| IL-18Rα | rs10167431 | Gene expression | 24013639 | European | Peripheral blood | IL18RAP |
| IL-18Rα | rs10167431 | Gene expression | eQTLGen | European | Whole blood | AC007278.3 |
| IL-18Rα | rs10167431 | Gene expression | eQTLGen | European | Whole blood | IL18RAP |
| IL-18Rα | rs10167431 | Gene expression | eQTLGen | European | Whole blood | MFSD9 |
| IL-18Rα | rs4140836 | Gene expression | eQTLGen | European | Whole blood | IL18RAP |
| IL-18Rα | rs4140836 | Gene expression | eQTLGen | European | Whole blood | IL1RL1 |
| IL-18Rα | rs4140836 | Gene expression | eQTLGen | European | Whole blood | AC007278.2 |
| IL-18Rα | rs17833274 | Gene expression | eQTLGen | European | Whole blood | IL18RAP |
| IL-18Rα | rs17833274 | Gene expression | eQTLGen | European | Whole blood | MFSD9 |
| IL-18Rα | rs114003263 | Gene expression | eQTLGen | European | Whole blood | IL18RAP |
| IL-18Rα | rs114003263 | Gene expression | eQTLGen | European | Whole blood | AC007278.3 |
| IL-18Rα | rs114003263 | Gene expression | eQTLGen | European | Whole blood | AC007278.2 |
| IL-18Rα | rs114003263 | Gene expression | eQTLGen | European | Whole blood | IL1RL1 |
| IL-18Rα | rs75778877 | Gene expression | eQTLGen | European | Whole blood | IL18RAP |
| IL-18Rα | rs75778877 | Gene expression | eQTLGen | European | Whole blood | AC007278.2 |
| IL-1α | rs13170249 | Gene expression | eQTLGen | European | Whole blood | PAM |
| IL-1α | rs13170249 | Gene expression | eQTLGen | European | Whole blood | PPIP5K2 |
| IL-1α | rs10418046 | Gene expression | 27918533 | European | Whole blood | NLRP12 |
| IL-1α | rs10418046 | Gene expression | eQTLGen | European | Whole blood | NLRP12 |
| IL-1R1 | rs112081515 | Gene expression | 27863251 | European | Neutrophils | DYM |
| IL-1R1 | rs112081515 | Gene expression | eQTLGen | European | Whole blood | DYM |
| IL-1R1 | rs112081515 | Gene expression | eQTLGen | European | Whole blood | RPL17 |
| IL-1R1 | rs10208542 | Gene expression | eQTLGen | European | Whole blood | IL18R1 |
| IL-1R1 | rs77451959 | Gene expression | eQTLGen | European | Whole blood | ARL14EP |
| IL-1R1 | rs12493830 | Gene expression | 27918533 | European | Whole blood | ST3GAL6-AS1 |
| IL-1R1 | rs12493830 | Gene expression | 27918533 | European | Whole blood | DCBLD2 |
| IL-1R1 | rs12493830 | Gene expression | 27918533 | European | Whole blood | ST3GAL6;RP11-319J24.3 |
| IL-1R1 | rs12493830 | Gene expression | eQTLGen | European | Whole blood | CPOX |
| IL-1R1 | rs12493830 | Gene expression | eQTLGen | European | Whole blood | DCBLD2 |
| IL-1R1 | rs12493830 | Gene expression | eQTLGen | European | Whole blood | RP11-319J24.3 |
| IL-1R1 | rs12493830 | Gene expression | eQTLGen | European | Whole blood | CLDND1 |
| IL-1R1 | rs11888059 | Gene expression | 27918533 | European | Whole blood | IL1R2 |
| IL-1R1 | rs11888059 | Gene expression | 27918533 | European | Whole blood | IL1R1 |
| IL-1R1 | rs11888059 | Gene expression | 24013639 | European | Peripheral blood | IL1R2 |
| IL-1R1 | rs11888059 | Gene expression | eQTLGen | European | Whole blood | IL1R2 |
| IL-1R1 | rs11888059 | Gene expression | eQTLGen | European | Whole blood | IL1R1 |
| IL-1R1 | rs10424405 | Gene expression | 27918533 | European | Whole blood | NLRP12 |
| IL-1R1 | rs10424405 | Gene expression | eQTLGen | European | Whole blood | NLRP12 |
| IL-1R1 | rs4690014 | Gene expression | eQTLGen | European | Whole blood | RP11-529E10.6 |
| IL-1R1 | rs4690014 | Gene expression | eQTLGen | European | Whole blood | HTT-AS1 |
| IL-1R1 | rs10972130 | Gene expression | 27918533 | European | Whole blood | NUDT2 |
| IL-1R1 | rs10972130 | Gene expression | 27918533 | European | Whole blood | SIGMAR1 |
| IL-1R1 | rs10972130 | Gene expression | eQTLGen | European | Whole blood | NUDT2 |
| IL-1R1 | rs10972130 | Gene expression | eQTLGen | European | Whole blood | SIGMAR1 |
| IL-1R1 | rs10972130 | Gene expression | eQTLGen | European | Whole blood | RP11-195F19.9 |
| IL-1Racp | rs1988743 | Gene expression | 27918533 | European | Whole blood | IL1RAP |
| IL-1Racp | rs1988743 | Gene expression | 24013639 | European | Peripheral blood | IL1RAP;AC108747.5-1 |
| IL-1Racp | rs6444435 | Gene expression | 27918533 | European | Whole blood | IL1RAP |
| IL-1Racp | rs6444435 | Gene expression | eQTLGen | European | Whole blood | IL1RAP |
| IL-1Racp | rs4815878 | Gene expression | 27918533 | European | Whole blood | SRXN1;PS1TP5 |
| IL-1Racp | rs4815878 | Gene expression | 27918533 | European | Whole blood | SRXN1 |
| IL-1Racp | rs4815878 | Gene expression | 27918533 | European | Whole blood | SCRT2 |
| IL-1Racp | rs4815878 | Gene expression | eQTLGen | European | Whole blood | SRXN1 |
| IL-1Racp | rs4815878 | Gene expression | eQTLGen | European | Whole blood | SCRT2 |
| IL-1Racp | rs61492644 | Gene expression | eQTLGen | European | Whole blood | TBC1D7 |
| IL-1Racp | rs5753690 | Gene expression | 27918533 | European | Whole blood | PISD |
| IL-1Racp | rs5753690 | Gene expression | 27918533 | European | Whole blood | SFI1 |
| IL-1Racp | rs5753690 | Gene expression | 27918533 | European | Whole blood | SFI1;PISD |
| IL-1Racp | rs5753690 | Gene expression | 27863251 | European | T cells | SFI1 |
| IL-1Racp | rs5753690 | Exon expression | 24037378 | European | Lymphoblastoid cell lines | SFI1 |
| IL-1Racp | rs5753690 | Gene expression | eQTLGen | European | Whole blood | PISD |
| IL-1Racp | rs5753690 | Gene expression | eQTLGen | European | Whole blood | PIK3IP1 |
| IL-1Racp | rs5753690 | Gene expression | eQTLGen | European | Whole blood | SFI1 |
| IL-1Racp | rs5753690 | Gene expression | eQTLGen | European | Whole blood | EIF4ENIF1 |
| IL-1Racp | rs5753690 | Gene expression | eQTLGen | European | Whole blood | YWHAH |
| IL-1Racp | rs5753690 | Gene expression | eQTLGen | European | Whole blood | RNF185 |
| IL-36α | rs704 | Gene expression | 27918533 | European | Whole blood | TMEM199;CTB-96E2.7 |
| IL-36α | rs704 | Gene expression | 27918533 | European | Whole blood | TMEM199;CTB-96E2.3 |
| IL-36α | rs704 | Gene expression | 27918533 | European | Whole blood | SLC46A1;CTD-2350C19.1 |
| IL-36α | rs704 | Gene expression | 27918533 | European | Whole blood | TMEM199;CTB-96E2.3;MIR4723 |
| IL-36α | rs704 | Gene expression | 27918533 | European | Whole blood | POLDIP2 |
| IL-36α | rs704 | Gene expression | 27918533 | European | Whole blood | TMEM97 |
| IL-36α | rs704 | Gene expression | 27918533 | European | Whole blood | POLDIP2;TNFAIP1 |
| IL-36α | rs704 | Gene expression | 27918533 | European | Whole blood | IFT20;TMEM97 |
| IL-36α | rs704 | Gene expression | 27863251 | European | Monocytes | TMEM97 |
| IL-36α | rs704 | Gene expression | 27863251 | European | Monocytes | TMEM199 |
| IL-36α | rs704 | Gene expression | 27863251 | European | T cells | TMEM97 |
| IL-36α | rs704 | Exon expression | 24037378 | European | Lymphoblastoid cell lines | TMEM97 |
| IL-36α | rs704 | Exon expression | 24037378 | European | Lymphoblastoid cell lines | TMEM97 |
| IL-36α | rs704 | Exon expression | 24037378 | European | Lymphoblastoid cell lines | TMEM199 |
| IL-36α | rs704 | Gene expression | 24037378 | European | Lymphoblastoid cell lines | TMEM97 |
| IL-36α | rs704 | Gene expression | 22941192 | European | Adipose subcutaneous | SARM1 |
| IL-36α | rs704 | Gene expression | 22941192 | European | Adipose subcutaneous | TMEM97 |
| IL-36α | rs704 | Gene expression | 22941192 | European | Lymphoblastoid cell lines | SARM1 |
| IL-36α | rs704 | Gene expression | 22941192 | European | Lymphoblastoid cell lines | TMEM97 |
| IL-36α | rs704 | Gene expression | 24013639 | European | Peripheral blood | TMEM97 |
| IL-36α | rs704 | Gene expression | 24013639 | European | Peripheral blood | SARM1;SLC46A1 |
| IL-36α | rs704 | Gene expression | eQTLGen | European | Whole blood | TMEM97 |
| IL-36α | rs704 | Gene expression | eQTLGen | European | Whole blood | SARM1 |
| IL-36α | rs704 | Gene expression | eQTLGen | European | Whole blood | CTD-2350C19.1 |
| IL-36α | rs704 | Gene expression | eQTLGen | European | Whole blood | TMEM199 |
| IL-36α | rs704 | Gene expression | eQTLGen | European | Whole blood | LGALS9 |
| IL-36α | rs704 | Gene expression | 25720628 | European | Visceral abdominal fat | TMEM199 |
| IL-36α | rs4239214 | Gene expression | eQTLGen | European | Whole blood | TMEM97 |
| IL-36α | rs4239214 | Gene expression | eQTLGen | European | Whole blood | SARM1 |
| IL-36α | rs62065286 | Gene expression | 27918533 | European | Whole blood | TMEM199;CTB-96E2.7 |
| IL-36α | rs62065286 | Gene expression | eQTLGen | European | Whole blood | TMEM97 |
| IL-36α | rs62065286 | Gene expression | eQTLGen | European | Whole blood | POLDIP2 |
| IL-36α | rs62065286 | Gene expression | eQTLGen | European | Whole blood | LGALS9 |
| IL-36α | rs74480769 | Gene expression | eQTLGen | European | Whole blood | PTGER4 |
| IL-36α | rs74480769 | Gene expression | 25720628 | European | Liver | C7 |
| IL-36α | rs74480769 | Gene expression | 25720628 | European | Atherosclerotic internal thoracic artery | C7 |
| IL-36α | rs74480769 | Gene expression | 25720628 | European | Visceral abdominal fat | C7 |
| IL-36α | rs10922094 | Gene expression | 27918533 | European | Whole blood | CFHR3 |
| IL-36α | rs10922094 | Gene expression | 27918533 | European | Whole blood | CFH |
| IL-36α | rs10922094 | Gene expression | eQTLGen | European | Whole blood | CFH |
| IL-36α | rs749331 | Gene expression | eQTLGen | European | Whole blood | UBXN2A |
| IL-36α | rs749331 | Gene expression | eQTLGen | European | Whole blood | TP53I3 |
| IL-36α | rs35995719 | Gene expression | eQTLGen | European | Whole blood | TMEM97 |
| IL-36α | rs72785161 | Gene expression | eQTLGen | European | Whole blood | SLC7A6 |
| IL-36α | rs72785161 | Gene expression | eQTLGen | European | Whole blood | DUS2L |
| IL-36α | rs72785161 | Gene expression | eQTLGen | European | Whole blood | DPEP2 |
| IL-36β | rs1905676 | Gene expression | eQTLGen | European | Whole blood | IPO8 |
| IL-36β | rs62143194 | Gene expression | 27918533 | European | Whole blood | NLRP12 |
| IL-36β | rs62143194 | Gene expression | eQTLGen | European | Whole blood | NLRP12 |
| IL-36β | rs139281982 | Gene expression | eQTLGen | European | Whole blood | RCHY1 |
| IL-36β | rs139281982 | Gene expression | eQTLGen | European | Whole blood | NAAA |
| IL-36β | rs56208304 | Gene expression | 27918533 | European | Whole blood | SH3TC1 |
| IL-36β | rs56208304 | Gene expression | 27918533 | European | Whole blood | SH3TC1;AC104650.2 |
| IL-36β | rs56208304 | Gene expression | eQTLGen | European | Whole blood | SH3TC1 |
| IL-36β | rs56208304 | Gene expression | eQTLGen | European | Whole blood | AC104650.2 |
| IL-36β | rs6704506 | Gene expression | eQTLGen | European | Whole blood | C1orf87 |
| IL-37 | rs117079751 | Gene expression | eQTLGen | European | Whole blood | ERC1 |
| IL-37 | rs74480769 | Gene expression | eQTLGen | European | Whole blood | PTGER4 |
| IL-37 | rs74480769 | Gene expression | 25720628 | European | Liver | C7 |
| IL-37 | rs74480769 | Gene expression | 25720628 | European | Atherosclerotic internal thoracic artery | C7 |
| IL-37 | rs74480769 | Gene expression | 25720628 | European | Visceral abdominal fat | C7 |
| IL-37 | rs11259827 | Gene expression | eQTLGen | European | Whole blood | CTGLF11P |
| IL-37 | rs6981787 | Gene expression | 27918533 | European | Whole blood | RP11-62H7.2 |
| IL-37 | rs6981787 | Gene expression | eQTLGen | European | Whole blood | RP11-10A14.5 |
| IL-37 | rs6981787 | Gene expression | eQTLGen | European | Whole blood | MFHAS1 |
| IL-37 | rs6981787 | Gene expression | eQTLGen | European | Whole blood | RP11-62H7.2 |
| IL-37 | rs737952 | Gene expression | eQTLGen | European | Whole blood | TCN2 |
| IL-37 | rs737952 | Gene expression | eQTLGen | European | Whole blood | DUSP18 |
| IL-37 | rs7539005 | Gene expression | 27918533 | European | Whole blood | CFHR3 |
| IL-37 | rs7539005 | Gene expression | 27918533 | European | Whole blood | CFH |
| IL-37 | rs7539005 | Gene expression | eQTLGen | European | Whole blood | CFH |
| IL-37 | rs62410788 | Gene expression | eQTLGen | European | Whole blood | CPEB2 |

# **Table S7.** Excluded *cis*-pQTLs that were associated with competing events related deaths

| Biomarkers | SNP | trait | Study | pmid | Ancestry | Year | beta | se | *P*-value |
| --- | --- | --- | --- | --- | --- | --- | --- | --- | --- |
| IL-18 | rs78623212 | Cause of death: appendix | Neale B | UKBB | European | 2017 | -0.007275 | 0.001313 | 3.08E-08 |
| IL-36β | rs78917327 | Cause of death: acute vascular disorders of intestine | Neale B | UKBB | European | 2017 | 0.01657 | 0.0028 | 3.40E-09 |
| IL-36β | rs76528994 | Cause of death: oropharynx, unspecified | Neale B | UKBB | European | 2017 | -0.009708 | 0.001544 | 3.40E-10 |

# **Table S8.** Sensitivity analysis for effects of the genetically predicted high circulating interleukin (IL)-1 family members/receptors on lung cancer, lung adenocarcinoma, and squamous cell lung cancer

| Exposures | Outcomes | Methods | No. of *cis*-pQTLs | OR | 95% CI | *P-* value | Q-statistic | *P* value for Q-statistic |
| --- | --- | --- | --- | --- | --- | --- | --- | --- |
| IL-1α | Lung cancer | MR IVW (fixed Effect) | 9 | 1.06 | 0.95 - 1.18 | 0.313 | 14.407 | 0.0718 |
|  |  | MR IVW (random Effect) | 9 | 1.06 | 0.91 - 1.22 | 0.452 |  |  |
|  |  | MR Weight Median | 9 | 1.06 | 0.9 - 1.25 | 0.465 |  |  |
|  |  | MR Egger regression | 9 | 1.19 | 0.71 - 2.01 | 0.526 |  |  |
|  |  | MR Egger Intercept (Pleiotropy) | 9 | -0.02 | -0.09 - 0.06 | 0.647 |  |  |
|  | Lung adenocarcinoma | MR IVW (fixed Effect) | 9 | 1.20 | 1.02 - 1.42 | 0.027 | 12.719 | 0.122 |
|  |  | MR IVW (random Effect) | 9 | 1.20 | 0.98 - 1.48 | 0.080 |  |  |
|  |  | MR Weight Median | 9 | 1.15 | 0.9 - 1.45 | 0.261 |  |  |
|  |  | MR Egger regression | 9 | 0.99 | 0.46 - 2.13 | 0.983 |  |  |
|  |  | MR Egger Intercept (Pleiotropy) | 9 | 0.03 | -0.08 - 0.14 | 0.618 |  |  |
|  | Squamous cell lung cancer | MR IVW (fixed Effect) | 9 | 0.89 | 0.76 - 1.05 | 0.176 | 10.926 | 0.206 |
|  |  | MR IVW (random Effect) | 9 | 0.89 | 0.73 - 1.08 | 0.247 |  |  |
|  |  | MR Weight Median | 9 | 0.85 | 0.67 - 1.08 | 0.178 |  |  |
|  |  | MR Egger regression | 9 | 1.08 | 0.56 - 2.08 | 0.829 |  |  |
|  |  | MR Egger Intercept (Pleiotropy) | 9 | -0.03 | -0.13 - 0.07 | 0.572 |  |  |
| IL-1β | Lung cancer | MR IVW (fixed Effect) | 3 | 0.93 | 0.7 - 1.25 | 0.642 | 5.617 | 0.0603 |
|  |  | MR IVW (random Effect) | 3 | 0.93 | 0.58 - 1.51 | 0.781 |  |  |
|  |  | MR Weight Median | 3 | 0.79 | 0.52 - 1.21 | 0.276 |  |  |
|  |  | MR Egger regression | 3 | 0.19 | 0.01 - 4.47 | 0.492 |  |  |
|  |  | MR Egger Intercept (Pleiotropy) | 3 | 0.13 | -0.13 - 0.4 | 0.501 |  |  |
|  | Lung adenocarcinoma | MR IVW (fixed Effect) | 3 | 1.03 | 0.65 - 1.63 | 0.891 | 5.532 | 0.0629 |
|  |  | MR IVW (random Effect) | 3 | 1.03 | 0.48 - 2.2 | 0.934 |  |  |
|  |  | MR Weight Median | 3 | 0.97 | 0.5 - 1.89 | 0.932 |  |  |
|  |  | MR Egger regression | 3 | 0.03 | 0 - 0.62 | 0.264 |  |  |
|  |  | MR Egger Intercept (Pleiotropy) | 3 | 0.30 | 0.05 - 0.54 | 0.259 |  |  |
|  | Squamous cell lung cancer | MR IVW (fixed Effect) | 3 | 0.78 | 0.5 - 1.21 | 0.262 | 8.193 | 0.0166 |
|  |  | MR IVW (random Effect) | 3 | 0.78 | 0.32 - 1.9 | 0.580 |  |  |
|  |  | MR Weight Median | 3 | 0.61 | 0.31 - 1.22 | 0.165 |  |  |
|  |  | MR Egger regression | 3 | 0.05 | 0 - 26.93 | 0.521 |  |  |
|  |  | MR Egger Intercept (Pleiotropy) | 3 | 0.23 | -0.29 - 0.76 | 0.545 |  |  |
| IL-1Ra | Lung cancer | MR IVW (fixed Effect) | 3 | 1.00 | 0.85 - 1.18 | 0.989 | 0.034 | 0.983 |
|  |  | MR IVW (random Effect) | 3 | 1.00 | 0.85 - 1.18 | 0.989 |  |  |
|  |  | MR Weight Median | 3 | 1.00 | 0.82 - 1.22 | 0.996 |  |  |
|  |  | MR Egger regression | 3 | 1.06 | 0.41 - 2.74 | 0.918 |  |  |
|  |  | MR Egger Intercept (Pleiotropy) | 3 | -0.01 | -0.14 - 0.12 | 0.915 |  |  |
|  | Lung adenocarcinoma | MR IVW (fixed Effect) | 3 | 1.02 | 0.79 - 1.32 | 0.886 | 2.334 | 0.311 |
|  |  | MR IVW (random Effect) | 3 | 1.02 | 0.77 - 1.35 | 0.895 |  |  |
|  |  | MR Weight Median | 3 | 1.14 | 0.82 - 1.59 | 0.433 |  |  |
|  |  | MR Egger regression | 3 | 0.34 | 0.08 - 1.48 | 0.387 |  |  |
|  |  | MR Egger Intercept (Pleiotropy) | 3 | 0.15 | -0.05 - 0.36 | 0.378 |  |  |
|  | Squamous cell lung cancer | MR IVW (fixed Effect) | 3 | 0.95 | 0.73 - 1.22 | 0.674 | 2.848 | 0.241 |
|  |  | MR IVW (random Effect) | 3 | 0.95 | 0.7 - 1.29 | 0.725 |  |  |
|  |  | MR Weight Median | 3 | 0.94 | 0.67 - 1.31 | 0.710 |  |  |
|  |  | MR Egger regression | 3 | 3.23 | 0.75 - 13.83 | 0.359 |  |  |
|  |  | MR Egger Intercept (Pleiotropy) | 3 | -0.17 | -0.38 - 0.03 | 0.342 |  |  |
| IL-1Racp | Lung cancer | MR IVW (fixed Effect) | 13 | 1.09 | 1.03 - 1.16 | 0.006 | 21.249 | 0.0469 |
|  |  | MR IVW (random Effect) | 13 | 1.09 | 1 - 1.19 | 0.039 |  |  |
|  |  | MR Weight Median | 13 | 1.14 | 1.04 - 1.25 | 0.006 |  |  |
|  |  | MR Egger regression | 13 | 1.14 | 0.94 - 1.37 | 0.205 |  |  |
|  |  | MR Egger Intercept (Pleiotropy) | 13 | -0.01 | -0.05 - 0.03 | 0.638 |  |  |
|  | Lung adenocarcinoma | MR IVW (fixed Effect) | 13 | 1.06 | 0.97 - 1.17 | 0.198 | 22.026 | 0.0372 |
|  |  | MR IVW (random Effect) | 13 | 1.06 | 0.94 - 1.21 | 0.342 |  |  |
|  |  | MR Weight Median | 13 | 1.07 | 0.94 - 1.22 | 0.298 |  |  |
|  |  | MR Egger regression | 13 | 1.09 | 0.82 - 1.45 | 0.570 |  |  |
|  |  | MR Egger Intercept (Pleiotropy) | 13 | -0.01 | -0.07 - 0.06 | 0.861 |  |  |
|  | Squamous cell lung cancer | MR IVW (fixed Effect) | 13 | 1.14 | 1.04 - 1.25 | 0.008 | 22.636 | 0.031 |
|  |  | MR IVW (random Effect) | 13 | 1.14 | 1 - 1.3 | 0.052 |  |  |
|  |  | MR Weight Median | 13 | 1.19 | 1.03 - 1.37 | 0.015 |  |  |
|  |  | MR Egger regression | 13 | 1.15 | 0.86 - 1.55 | 0.366 |  |  |
|  |  | MR Egger Intercept (Pleiotropy) | 13 | 0.00 | -0.07 - 0.06 | 0.925 |  |  |
| IL-18 | Lung cancer | MR IVW (fixed Effect) | 8 | 1.03 | 0.92 - 1.16 | 0.619 | 12.241 | 0.0929 |
|  |  | MR IVW (random Effect) | 8 | 1.03 | 0.88 - 1.2 | 0.707 |  |  |
|  |  | MR Weight Median | 8 | 1.01 | 0.85 - 1.19 | 0.916 |  |  |
|  |  | MR Egger regression | 8 | 1.25 | 0.98 - 1.6 | 0.116 |  |  |
|  |  | MR Egger Intercept (Pleiotropy) | 8 | -0.05 | -0.09 - 0 | 0.106 |  |  |
|  | Lung adenocarcinoma | MR IVW (fixed Effect) | 8 | 0.93 | 0.78 - 1.1 | 0.404 | 13.781 | 0.0552 |
|  |  | MR IVW (random Effect) | 8 | 0.93 | 0.73 - 1.18 | 0.552 |  |  |
|  |  | MR Weight Median | 8 | 0.94 | 0.75 - 1.19 | 0.617 |  |  |
|  |  | MR Egger regression | 8 | 1.08 | 0.69 - 1.68 | 0.756 |  |  |
|  |  | MR Egger Intercept (Pleiotropy) | 8 | -0.04 | -0.13 - 0.05 | 0.467 |  |  |
|  | Squamous cell lung cancer | MR IVW (fixed Effect) | 8 | 1.04 | 0.87 - 1.24 | 0.665 | 7.251 | 0.403 |
|  |  | MR IVW (random Effect) | 8 | 1.04 | 0.87 - 1.25 | 0.670 |  |  |
|  |  | MR Weight Median | 8 | 1.11 | 0.88 - 1.4 | 0.384 |  |  |
|  |  | MR Egger regression | 8 | 1.44 | 1.04 - 2 | 0.070 |  |  |
|  |  | MR Egger Intercept (Pleiotropy) | 8 | -0.08 | -0.14 - -0.01 | 0.058 |  |  |
| IL-18BP | Lung cancer | MR IVW (fixed Effect) | 6 | 0.93 | 0.81 - 1.06 | 0.292 | 10.348 | 0.066 |
|  |  | MR IVW (random Effect) | 6 | 0.93 | 0.77 - 1.13 | 0.464 |  |  |
|  |  | MR Weight Median | 6 | 0.96 | 0.79 - 1.18 | 0.716 |  |  |
|  |  | MR Egger regression | 6 | 0.69 | 0.5 - 0.96 | 0.094 |  |  |
|  |  | MR Egger Intercept (Pleiotropy) | 6 | 0.07 | 0 - 0.14 | 0.118 |  |  |
|  | Lung adenocarcinoma | MR IVW (fixed Effect) | 6 | 0.91 | 0.75 - 1.11 | 0.343 | 4.055 | 0.542 |
|  |  | MR IVW (random Effect) | 6 | 0.91 | 0.75 - 1.11 | 0.343 |  |  |
|  |  | MR Weight Median | 6 | 0.96 | 0.75 - 1.24 | 0.781 |  |  |
|  |  | MR Egger regression | 6 | 0.73 | 0.48 - 1.11 | 0.213 |  |  |
|  |  | MR Egger Intercept (Pleiotropy) | 6 | 0.05 | -0.04 - 0.14 | 0.307 |  |  |
|  | Squamous cell lung cancer | MR IVW (fixed Effect) | 6 | 0.85 | 0.7 - 1.03 | 0.100 | 6.648 | 0.248 |
|  |  | MR IVW (random Effect) | 6 | 0.85 | 0.68 - 1.06 | 0.153 |  |  |
|  |  | MR Weight Median | 6 | 0.80 | 0.62 - 1.04 | 0.094 |  |  |
|  |  | MR Egger regression | 6 | 0.58 | 0.38 - 0.88 | 0.062 |  |  |
|  |  | MR Egger Intercept (Pleiotropy) | 6 | 0.09 | 0 - 0.18 | 0.112 |  |  |
| IL-1R1 | Lung cancer | MR IVW (fixed Effect) | 8 | 1.06 | 0.95 - 1.19 | 0.321 | 3.772 | 0.806 |
|  |  | MR IVW (random Effect) | 8 | 1.06 | 0.95 - 1.19 | 0.321 |  |  |
|  |  | MR Weight Median | 8 | 1.04 | 0.89 - 1.21 | 0.620 |  |  |
|  |  | MR Egger regression | 8 | 1.08 | 0.87 - 1.34 | 0.504 |  |  |
|  |  | MR Egger Intercept (Pleiotropy) | 8 | -0.01 | -0.05 - 0.04 | 0.838 |  |  |
|  | Lung adenocarcinoma | MR IVW (fixed Effect) | 8 | 1.01 | 0.86 - 1.2 | 0.881 | 4.519 | 0.718 |
|  |  | MR IVW (random Effect) | 8 | 1.01 | 0.86 - 1.2 | 0.881 |  |  |
|  |  | MR Weight Median | 8 | 1.11 | 0.88 - 1.4 | 0.377 |  |  |
|  |  | MR Egger regression | 8 | 0.85 | 0.63 - 1.15 | 0.328 |  |  |
|  |  | MR Egger Intercept (Pleiotropy) | 8 | 0.05 | -0.02 - 0.13 | 0.219 |  |  |
|  | Squamous cell lung cancer | MR IVW (fixed Effect) | 8 | 1.12 | 0.94 - 1.33 | 0.215 | 2.436 | 0.932 |
|  |  | MR IVW (random Effect) | 8 | 1.12 | 0.94 - 1.33 | 0.215 |  |  |
|  |  | MR Weight Median | 8 | 1.15 | 0.93 - 1.42 | 0.199 |  |  |
|  |  | MR Egger regression | 8 | 1.12 | 0.81 - 1.55 | 0.528 |  |  |
|  |  | MR Egger Intercept (Pleiotropy) | 8 | 0.00 | -0.08 - 0.08 | 0.995 |  |  |
| IL-36α | Lung cancer | MR IVW (fixed Effect) | 4 | 1.04 | 0.9 - 1.21 | 0.582 | 1.962 | 0.58 |
|  |  | MR IVW (random Effect) | 4 | 1.04 | 0.9 - 1.21 | 0.582 |  |  |
|  |  | MR Weight Median | 4 | 1.05 | 0.88 - 1.26 | 0.562 |  |  |
|  |  | MR Egger regression | 4 | 1.20 | 0.63 - 2.3 | 0.632 |  |  |
|  |  | MR Egger Intercept (Pleiotropy) | 4 | -0.02 | -0.12 - 0.07 | 0.698 |  |  |
|  | Lung adenocarcinoma | MR IVW (fixed Effect) | 4 | 1.02 | 0.81 - 1.27 | 0.891 | 2.741 | 0.433 |
|  |  | MR IVW (random Effect) | 4 | 1.02 | 0.81 - 1.27 | 0.891 |  |  |
|  |  | MR Weight Median | 4 | 1.03 | 0.78 - 1.36 | 0.847 |  |  |
|  |  | MR Egger regression | 4 | 1.09 | 0.33 - 3.53 | 0.902 |  |  |
|  |  | MR Egger Intercept (Pleiotropy) | 4 | -0.01 | -0.18 - 0.16 | 0.918 |  |  |
|  | Squamous cell lung cancer | MR IVW (fixed Effect) | 4 | 1.07 | 0.86 - 1.33 | 0.567 | 2.727 | 0.436 |
|  |  | MR IVW (random Effect) | 4 | 1.07 | 0.86 - 1.33 | 0.567 |  |  |
|  |  | MR Weight Median | 4 | 1.01 | 0.77 - 1.33 | 0.921 |  |  |
|  |  | MR Egger regression | 4 | 0.94 | 0.3 - 2.91 | 0.922 |  |  |
|  |  | MR Egger Intercept (Pleiotropy) | 4 | 0.02 | -0.15 - 0.19 | 0.841 |  |  |
| IL-36β | Lung cancer | MR IVW (fixed Effect) | 6 | 1.03 | 0.92 - 1.16 | 0.610 | 1.852 | 0.869 |
|  |  | MR IVW (random Effect) | 6 | 1.03 | 0.92 - 1.16 | 0.610 |  |  |
|  |  | MR Weight Median | 6 | 1.02 | 0.87 - 1.18 | 0.835 |  |  |
|  |  | MR Egger regression | 6 | 0.95 | 0.75 - 1.2 | 0.679 |  |  |
|  |  | MR Egger Intercept (Pleiotropy) | 6 | 0.02 | -0.03 - 0.07 | 0.464 |  |  |
|  | Lung adenocarcinoma | MR IVW (fixed Effect) | 6 | 1.17 | 0.98 - 1.39 | 0.090 | 3.238 | 0.663 |
|  |  | MR IVW (random Effect) | 6 | 1.17 | 0.98 - 1.39 | 0.090 |  |  |
|  |  | MR Weight Median | 6 | 1.11 | 0.88 - 1.41 | 0.373 |  |  |
|  |  | MR Egger regression | 6 | 0.97 | 0.68 - 1.38 | 0.860 |  |  |
|  |  | MR Egger Intercept (Pleiotropy) | 6 | 0.05 | -0.03 - 0.12 | 0.299 |  |  |
|  | Squamous cell lung cancer | MR IVW (fixed Effect) | 6 | 0.98 | 0.83 - 1.17 | 0.861 | 0.358 | 0.996 |
|  |  | MR IVW (random Effect) | 6 | 0.98 | 0.83 - 1.17 | 0.861 |  |  |
|  |  | MR Weight Median | 6 | 1.00 | 0.81 - 1.23 | 0.994 |  |  |
|  |  | MR Egger regression | 6 | 0.93 | 0.66 - 1.32 | 0.720 |  |  |
|  |  | MR Egger Intercept (Pleiotropy) | 6 | 0.01 | -0.06 - 0.09 | 0.749 |  |  |
| IL-36γ | Lung cancer | MR IVW (fixed Effect) | 5 | 0.97 | 0.85 - 1.1 | 0.605 | 11.363 | 0.0228 |
|  |  | MR IVW (random Effect) | 5 | 0.97 | 0.78 - 1.2 | 0.759 |  |  |
|  |  | MR Weight Median | 5 | 1.06 | 0.88 - 1.29 | 0.527 |  |  |
|  |  | MR Egger regression | 5 | 0.66 | 0.29 - 1.48 | 0.386 |  |  |
|  |  | MR Egger Intercept (Pleiotropy) | 5 | 0.10 | -0.1 - 0.29 | 0.406 |  |  |
|  | Lung adenocarcinoma | MR IVW (fixed Effect) | 5 | 0.88 | 0.73 - 1.07 | 0.207 | 11.805 | 0.0189 |
|  |  | MR IVW (random Effect) | 5 | 0.88 | 0.64 - 1.23 | 0.463 |  |  |
|  |  | MR Weight Median | 5 | 0.84 | 0.64 - 1.11 | 0.211 |  |  |
|  |  | MR Egger regression | 5 | 0.82 | 0.21 - 3.2 | 0.798 |  |  |
|  |  | MR Egger Intercept (Pleiotropy) | 5 | 0.02 | -0.31 - 0.34 | 0.923 |  |  |
|  | Squamous cell lung cancer | MR IVW (fixed Effect) | 5 | 1.08 | 0.89 - 1.32 | 0.430 | 4.851 | 0.303 |
|  |  | MR IVW (random Effect) | 5 | 1.08 | 0.87 - 1.35 | 0.473 |  |  |
|  |  | MR Weight Median | 5 | 0.95 | 0.73 - 1.24 | 0.711 |  |  |
|  |  | MR Egger regression | 5 | 0.71 | 0.31 - 1.66 | 0.487 |  |  |
|  |  | MR Egger Intercept (Pleiotropy) | 5 | 0.11 | -0.1 - 0.31 | 0.386 |  |  |
| IL-18Rα | Lung cancer | MR IVW (fixed Effect) | 9 | 1.05 | 0.94 - 1.18 | 0.347 | 9.243 | 0.322 |
|  |  | MR IVW (random Effect) | 9 | 1.05 | 0.94 - 1.19 | 0.382 |  |  |
|  |  | MR Weight Median | 9 | 1.05 | 0.9 - 1.23 | 0.530 |  |  |
|  |  | MR Egger regression | 9 | 1.36 | 1 - 1.85 | 0.092 |  |  |
|  |  | MR Egger Intercept (Pleiotropy) | 9 | -0.05 | -0.1 - 0.01 | 0.128 |  |  |
|  | Lung adenocarcinoma | MR IVW (fixed Effect) | 9 | 1.14 | 0.96 - 1.36 | 0.126 | 9.066 | 0.337 |
|  |  | MR IVW (random Effect) | 9 | 1.14 | 0.95 - 1.38 | 0.151 |  |  |
|  |  | MR Weight Median | 9 | 1.05 | 0.84 - 1.31 | 0.693 |  |  |
|  |  | MR Egger regression | 9 | 1.87 | 1.13 - 3.09 | 0.044 |  |  |
|  |  | MR Egger Intercept (Pleiotropy) | 9 | -0.09 | -0.18 - 0 | 0.079 |  |  |
|  | Squamous cell lung cancer | MR IVW (fixed Effect) | 9 | 0.93 | 0.78 - 1.09 | 0.361 | 20.473 | 0.00869 |
|  |  | MR IVW (random Effect) | 9 | 0.93 | 0.71 - 1.21 | 0.568 |  |  |
|  |  | MR Weight Median | 9 | 0.96 | 0.75 - 1.22 | 0.736 |  |  |
|  |  | MR Egger regression | 9 | 0.78 | 0.38 - 1.62 | 0.530 |  |  |
|  |  | MR Egger Intercept (Pleiotropy) | 9 | 0.03 | -0.1 - 0.16 | 0.639 |  |  |
| IL-37 | Lung cancer | MR IVW (fixed Effect) | 5 | 1.04 | 0.9 - 1.2 | 0.624 | 7.401 | 0.116 |
|  |  | MR IVW (random Effect) | 5 | 1.04 | 0.85 - 1.26 | 0.719 |  |  |
|  |  | MR Weight Median | 5 | 1.04 | 0.85 - 1.27 | 0.701 |  |  |
|  |  | MR Egger regression | 5 | 0.93 | 0.36 - 2.37 | 0.881 |  |  |
|  |  | MR Egger Intercept (Pleiotropy) | 5 | 0.02 | -0.13 - 0.17 | 0.823 |  |  |
|  | Lung adenocarcinoma | MR IVW (fixed Effect) | 5 | 0.96 | 0.77 - 1.2 | 0.717 | 2.603 | 0.626 |
|  |  | MR IVW (random Effect) | 5 | 0.96 | 0.77 - 1.2 | 0.717 |  |  |
|  |  | MR Weight Median | 5 | 1.03 | 0.78 - 1.35 | 0.857 |  |  |
|  |  | MR Egger regression | 5 | 0.65 | 0.26 - 1.64 | 0.427 |  |  |
|  |  | MR Egger Intercept (Pleiotropy) | 5 | 0.06 | -0.08 - 0.21 | 0.456 |  |  |
|  | Squamous cell lung cancer | MR IVW (fixed Effect) | 5 | 1.02 | 0.82 - 1.27 | 0.841 | 5.489 | 0.241 |
|  |  | MR IVW (random Effect) | 5 | 1.02 | 0.79 - 1.32 | 0.864 |  |  |
|  |  | MR Weight Median | 5 | 1.03 | 0.76 - 1.39 | 0.861 |  |  |
|  |  | MR Egger regression | 5 | 0.71 | 0.22 - 2.33 | 0.611 |  |  |
|  |  | MR Egger Intercept (Pleiotropy) | 5 | 0.06 | -0.13 - 0.25 | 0.578 |  |  |

# **Table S9.** Conditional F-statistic for testing instrument strength in the robust multivariable Mendelian randomization analysis via Models 1, 2, 3, and 4

| **Model 1** |  | **IL-1α** | **IL-1β** | **IL-1Ra** | **Overall** | - | - | - | - | - | - | - | - | - |
| --- | --- | --- | --- | --- | --- | --- | --- | --- | --- | --- | --- | --- | --- | --- |
|  | **IL-1α** | N.A. | 13.94 | 13.85 | 4.66 | - | - | - | - | - | - | - | - | - |
|  | **IL-1β** | 1.64 | N.A. | 1.10 | 0.68 | - | - | - | - | - | - | - | - | - |
|  | **IL-1Ra** | 6.42 | 2.66 | N.A. | 0.61 | - | - | - | - | - | - | - | - | - |
| **Model 2** |  | **IL-18** | **IL-18BP** | **IL-37** | **Overall** | - | - | - | - | - | - | - | - | - |
|  | **IL-18** | N.A. | 11.91 | 11.89 | 12.14 | - | - | - | - | - | - | - | - | - |
|  | **IL-18BP** | 8.31 | N.A. | 3.68 | 4.67 | - | - | - | - | - | - | - | - | - |
|  | **IL-37** | 8.72 | 3.25 | N.A. | 4.46 | - | - | - | - | - | - | - | - | - |
| **Model 3** |  | **IL-36α** | **IL-36β** | **IL-36γ** | **Overal** | - | - | - | - | - | - | - | - | - |
|  | **IL-36α** | N.A. | 9.13 | 6.80 | 7.27 | - | - | - | - | - | - | - | - | - |
|  | **IL-36β** | 12.42 | N.A. | 8.61 | 9.29 | - | - | - | - | - | - | - | - | - |
|  | **IL-36γ** | 7.77 | 9.13 | N.A. | 7.06 | - | - | - | - | - | - | - | - | - |
| **Model 4** |  | **IL-1α** | **IL-1β** | **IL-1Ra** | **IL-18** | **IL-18BP** | **IL-37** | **IL-36α** | **IL-36β** | **IL-36γ** | **IL-1Racp** | **IL-18Rα** | **IL-1R1** | **Overall** |
|  | **IL-1α** | N.A. | 4.53 | 4.10 | 4.54 | 2.20 | 2.50 | 3.04 | 3.67 | 3.90 | 4.56 | 1.98 | 3.65 | 2.31 |
|  | **IL-1β** | 2.28 | N.A. | 0.55 | 1.87 | 2.01 | 2.00 | 2.13 | 2.05 | 2.04 | 2.03 | 1.97 | 1.98 | 0.62 |
|  | **IL-1Ra** | 3.28 | 0.99 | N.A. | 2.58 | 2.30 | 2.41 | 2.52 | 2.53 | 2.54 | 2.56 | 2.23 | 2.49 | 0.78 |
|  | **IL-18** | 4.07 | 5.69 | 3.91 | N.A. | 3.95 | 3.97 | 3.67 | 3.71 | 3.66 | 3.89 | 3.57 | 3.80 | 6.04 |
|  | **IL-18BP** | 2.16 | 6.09 | 3.53 | 4.02 | N.A. | 1.65 | 2.47 | 3.97 | 3.21 | 4.34 | 1.43 | 2.30 | 2.83 |
|  | **IL-37** | 2.17 | 5.90 | 3.44 | 3.83 | 1.44 | N.A. | 0.59 | 3.98 | 2.04 | 3.94 | 2.31 | 2.04 | 1.80 |
|  | **IL-36α** | 2.41 | 5.94 | 3.67 | 3.34 | 2.02 | 0.56 | N.A. | 3.63 | 2.21 | 3.59 | 3.12 | 2.08 | 1.10 |
|  | **IL-36β** | 2.88 | 4.37 | 2.80 | 2.74 | 3.14 | 3.57 | 3.44 | N.A. | 3.06 | 3.48 | 2.75 | 3.41 | 0.64 |
|  | **IL-36γ** | 3.46 | 5.29 | 3.50 | 3.52 | 2.87 | 2.11 | 2.52 | 3.54 | N.A. | 3.99 | 3.20 | 0.85 | 2.00 |
|  | **IL-1Racp** | 6.08 | 7.58 | 5.06 | 5.05 | 5.93 | 6.06 | 5.98 | 6.04 | 6.07 | N.A. | 6.21 | 6.11 | 1.04 |
|  | **IL-18Rα** | 2.16 | 6.61 | 3.67 | 4.32 | 1.50 | 2.89 | 4.26 | 3.87 | 3.82 | 4.96 | N.A. | 3.47 | 1.52 |
|  | **IL-1R1** | 2.37 | 4.14 | 2.65 | 2.74 | 1.49 | 1.59 | 1.80 | 2.82 | 0.62 | 2.83 | 2.14 | N.A. | 0.60 |

# **Table S10.** Robust multivariable Mendelian randomization estimates for the genetically predicted high circulating interleukin (IL)-1 family members/receptors on lung cancer, lung adenocarcinoma, and squamous cell lung cancer after removing interleukin-1 receptor antagonist (IL-1β), interleukin-36α (IL-36α), and interleukin-37 (IL-37)

| Exposure | # SNPs | Conditional F-statistic* | Adjust for | Lung cancer | | | | | | |
| --- | --- | --- | --- | --- | --- | --- | --- | --- | --- | --- |
|  |  |  |  | Multivariable Mendelian randomization analysis | | |  | Multivariable MR Egger intercept** | | |
|  |  |  |  | Odds ratio | 95% CI | P value |  | beta | se | P value |
| IL-1α (n = 3301) |  |  |  |  |  |  |  |  |  |  |
| Model 1 | 11 | 14.32 | IL-1Ra | 1.07 | 0.96 to 1.20 | 0.217 |  | -0.006 | 0.011 | 0.564 |
| Model 4 | 62 | 2.29 | IL-1 family*** | 1.07 | 0.92 to 1.26 | 0.375 |  | -0.005 | 0.007 | 0.450 |
| IL-1Ra (n = 3309) |  |  |  |  |  |  |  |  |  |  |
| Model 1 | 11 | 7.90 | IL-1α | **0.97** | **0.93 to 1.01** | **0.201** |  | -0.014 | 0.014 | 0.321 |
| Model 4 | 62 | 2.62 | IL-1 family | **0.97** | **0.90 to 1.04** | **0.339** |  | -0.008 | 0.005 | 0.146 |
| IL-1R1 (n = 3301) |  |  |  |  |  |  |  |  |  |  |
| Model 4 | 62 | 1.05 | IL-1 family | 1.03 | 0.91 to 1.17 | 0.670 |  | 0 | 0.006 | 0.996 |
| IL-1Racp (n = 3301) |  |  |  |  |  |  |  |  |  |  |
| Model 4 | 62 | 5.89 | IL-1 family | 1.03 | 0.88 to 1.20 | 0.739 |  | 0 | 0.006 | 0.973 |
| IL-18 (n = 3636) |  |  |  |  |  |  |  |  |  |  |
| Model 2 | 12 | 16.99 | IL-18BP | 1.05 | 0.98 to 1.14 | 0.172 |  | -0.008 | 0.025 | 0.733 |
| Model 4 | 62 | 3.46 | IL-1 family | 0.99 | 0.88 to 1.11 | 0.855 |  | -0.004 | 0.005 | 0.331 |
| IL-18Rα (n = 3301) |  |  |  |  |  |  |  |  |  |  |
| Model 4 | 62 | 1.61 | IL-1 family | 0.98 | 0.84 to 1.14 | 0.763 |  | 0.004 | 0.007 | 0.638 |
| IL-18BP (n = 3301) |  |  |  |  |  |  |  |  |  |  |
| Model 2 | 12 | 9.98 | IL-18BP | 0.94 | 0.85 to 1.04 | 0.232 |  | 0.058 | 0.013 | 0.000 |
| Model 4 | 62 | 1.73 | IL-1 family | 1.08 | 0.90 to 1.29 | 0.389 |  | 0.01 | 0.008 | 0.233 |
| IL-36β (n = 3301) |  |  |  |  |  |  |  |  |  |  |
| Model 3 | 9 | 12.42 | IL-36γ | 0.97 | 0.90 to 1.05 | 0.473 |  | -0.003 | 0.014 | 0.821 |
| Model 4 | 62 | 2.06 | IL-1 family | 1.03 | 0.93 to 1.15 | 0.536 |  | 0.001 | 0.007 | 0.929 |
| IL-36γ (n = 3301) |  |  |  |  |  |  |  |  |  |  |
| Model 3 | 9 | 11.90 | IL-36β | 1.15 | 1.04 to 1.28 | 0.009 |  | 0.03 | NA | NA |
| Model 4 | 62 | 1.00 | IL-1 family | 0.95 | 0.78 to 1.17 | 0.650 |  | -0.003 | NA | NA |
| Exposure | # SNPs | Conditional F-statistic* | Adjust for | Lung adenocarcinoma | | | | | | |
|  |  |  |  | Multivariable Mendelian randomization analysis | | |  | Multivariable MR Egger intercept** | | |
|  |  |  |  | Odds ratio | 95% CI | P value |  | beta | se | P value |
| IL-1α (n = 3301) |  |  |  |  |  |  |  |  |  |  |
| Model 1 | 11 | 14.32 | IL-1β | **1.28** | **1.08 to 1.52** | **0.004** |  | 0.030 | 0.015 | 0.047 |
| Model 4 | 62 | 2.29 | IL-1 family*** | **1.35** | **1.07 to 1.69** | **0.010** |  | 0.015 | 0.009 | 0.113 |
| IL-1Ra (n = 3309) |  |  |  |  |  |  |  |  |  |  |
| Model 1 | 11 | 7.90 | IL-1α | 0.95 | 0.80 to 1.13 | 0.584 |  | -0.028 | 0.020 | 0.156 |
| Model 4 | 62 | 2.62 | IL-1 family | 0.99 | 0.82 to 1.21 | 0.943 |  | -0.008 | 0.009 | 0.340 |
| IL-1R1 (n = 3301) |  |  |  |  |  |  |  |  |  |  |
| Model 4 | 62 | 1.05 | IL-1 family | 1.14 | 0.86 to 1.51 | 0.367 |  | -0.003 | 0.008 | 0.701 |
| IL-1Racp (n = 3301) |  |  |  |  |  |  |  |  |  |  |
| Model 4 | 62 | 5.89 | IL-1 family | 0.96 | 0.82 to 1.13 | 0.632 |  | 0.004 | 0.007 | 0.583 |
| IL-18 (n = 3636) |  |  |  |  |  |  |  |  |  |  |
| Model 2 | 12 | 16.99 | IL-18BP | 1.00 | 0.88 to 1.14 | 0.991 |  | 0.980 | 0.85 to 1.13 | 0.821 |
| Model 4 | 62 | 3.46 | IL-1 family | 0.95 | 0.78 to 1.14 | 0.558 |  | 0.940 | 0.82 to 1.09 | 0.447 |
| IL-18R1 (n = 3301) |  |  |  |  |  |  |  |  |  |  |
| Model 4 | 62 | 1.61 | IL-1 family | 1.17 | 0.93 to 1.46 | 0.176 |  | 0.013 | 0.018 | 0.456 |
| IL-18BP (n = 3301) |  |  |  |  |  |  |  |  |  |  |
| Model 2 | 12 | 9.98 | IL-18BP | 0.92 | 0.80 to 1.05 | 0.201 |  | 0.064 | 0.025 | 0.011 |
| Model 4 | 62 | 1.73 | IL-1 family | 1.06 | 0.81 to 1.40 | 0.661 |  | 0.020 | 0.015 | 0.178 |
| IL-36β (n = 3301) |  |  |  |  |  |  |  |  |  |  |
| Model 3 | 9 | 12.42 | IL-36γ | 1.13 | 0.95 to 1.34 | 0.163 |  | -0.060 | 0.043 | 0.166 |
| Model 4 | 62 | 2.06 | IL-1 family | 1.14 | 0.97 to 1.34 | 0.111 |  | 0.003 | 0.010 | 0.726 |
| IL-36γ (n = 3301) |  |  |  |  |  |  |  |  |  |  |
| Model 3 | 9 | 11.90 | IL-36β | 0.84 | 0.56 to 1.25 | 0.389 |  | 0.068 | 0.041 | 0.099 |
| Model 4 | 62 | 1.00 | IL-1 family | 0.84 | 0.56 to 1.26 | 0.410 |  | 0.003 | 0.011 | 0.758 |
| Exposure | # SNPs | Conditional F-statistic* | Adjust for | Squamous cell lung cancer | | | | | | |
|  |  |  |  | Multivariable Mendelian randomization analysis | | |  | Multivariable MR Egger intercept** | | |
|  |  |  |  | Odds ratio | 95% CI | P value |  | beta | se | P value |
| IL-1α (n = 3301) |  |  |  |  |  |  |  |  |  |  |
| Model 1 | 11 | 14.32 | IL-1β | 0.89 | 0.74 to 1.05 | 0.168 |  | -0.032 | 0.010 | 0.002 |
| Model 4 | 62 | 2.29 | IL-1 family*** | 0.84 | 0.66 to 1.06 | 0.144 |  | -0.019 | 0.012 | 0.104 |
| IL-1Ra (n = 3309) |  |  |  |  |  |  |  |  |  |  |
| Model 1 | 11 | 7.90 | IL-1α | 0.95 | 0.80 to 1.13 | 0.588 |  | -0.007 | 0.023 | 0.767 |
| Model 4 | 62 | 2.62 | IL-1 family | **0.89** | **0.78 to 1.00** | **0.053** |  | -0.009 | 0.009 | 0.359 |
| IL-1R1 (n = 3301) |  |  |  |  |  |  |  |  |  |  |
| Model 4 | 62 | 1.05 | IL-1 family | 1.06 | 0.88 to 1.27 | 0.565 |  | 0.004 | 0.009 | 0.638 |
| IL-1Racp (n = 3301) |  |  |  |  |  |  |  |  |  |  |
| Model 4 | 62 | 5.89 | IL-1 family | 1.05 | 0.90 to 1.23 | 0.534 |  | 0.003 | 0.009 | 0.732 |
| IL-18 (n = 3636) |  |  |  |  |  |  |  |  |  |  |
| Model 2 | 12 | 16.99 | IL-18BP | 0.98 | 0.85 to 1.13 | 0.821 |  | -0.092 | 0.015 | 0.000 |
| Model 4 | 62 | 3.46 | IL-1 family | 0.94 | 0.82 to 1.09 | 0.447 |  | 0.002 | 0.009 | 0.807 |
| IL-18R1 (n = 3301) |  |  |  |  |  |  |  |  |  |  |
| Model 4 | 62 | 1.61 | IL-1 family | 0.88 | 0.68 to 1.14 | 0.334 |  | 0.010 | 0.012 | 0.411 |
| IL-18BP (n = 3301) |  |  |  |  |  |  |  |  |  |  |
| Model 2 | 12 | 9.98 | IL-18BP | **0.84** | **0.71 to 1.00** | **0.045** |  | 0.072 | 0.017 | 0.000 |
| Model 4 | 62 | 1.73 | IL-1 family | 0.91 | 0.73 to 1.15 | 0.435 |  | 0.012 | 0.012 | 0.291 |
| IL-36β (n = 3301) |  |  |  |  |  |  |  |  |  |  |
| Model 3 | 9 | 12.42 | IL-36γ | 0.95 | 0.72 to 1.26 | 0.715 |  | 0.014 | 0.045 | 0.750 |
| Model 4 | 62 | 2.06 | IL-1 family | 1.00 | 0.88 to 1.14 | 0.999 |  | -0.001 | 0.012 | 0.927 |
| IL-36γ (n = 3301) |  |  |  |  |  |  |  |  |  |  |
| Model 3 | 9 | 11.90 | IL-36β | 1.11 | 0.34 to 3.63 | 0.863 |  | 0.006 | 0.025 | 0.807 |
| Model 4 | 62 | 1.00 | IL-1 family | 1.08 | 0.88 to 1.32 | 0.464 |  | 0.032 | NA | NA |

* The maximum Q statistic for testing the instrument strength for each exposure under different models.

* The multivariable Egger regression analysis is conducted after harmonising the dataset with the allele-coding of exposure of interest as reference.

*** IL-1α: interleukin-1 α; IL-1β: interleukin-1 β; IL-1Ra: interleukin-1 receptor antagonist; IL-18: interleukin-18; IL-18BP: interleukin-18 binding; IL-1R1: interleukin-1 receptor-type 1; IL-18Rα: interleukin-18 receptor 1; IL-36α/β/γ: interleukin-36 α/β/γ; IL-37: interleukin-37.

Note. Lung cancer: Q statistics for testing the instrument validity. Model 1 included IL-1α and IL-1Ra. Q-statistic = 13.95, P value = 0.124; Model 2 included IL-18 and IL-18BP. Q-statistic = 19.79, P value = 0.031; and Model 3 included IL-36β and IL-36γ. Q-statistic = 18.16, P value = 0.011; Model 4 included IL-1α, IL-1Ra, IL-1R1, IL-1Racp, IL-18, IL-18BP, IL-36β, and Il-36γ. Q-statistic = 82.30, P value = 0.0296. Lung adenocarcinoma: Model 1. Q-statistic = 12.64, P value = 0.18; Model 2. Q-statistic = 10.62, P value = 0.387; and Model 3 Q-statistic = 11.17, P value = 0.131; Model 4 Q-statistic = 54.50, P value = 0.676. Squamous cell lung cancer: Model 1. Q-statistic = 13.30, P value = 0.149; Model 2. Q-statistic = 13.63, P value = 0.191; and Model 3 Q-statistic = 3.90, P value = 0.792; Model 4 Q-statistic = 67.22; P value = 0.244.

# **Table S11.** The IL-1Ra-rheumatoid arthritis associations using the inverse weighted methods based on the latest GWAS datasets

| ID in MR-base | Exposure | Outcome | # SNP | Beta | se | *P*-value | No. cases | No. controls | Year |
| --- | --- | --- | --- | --- | --- | --- | --- | --- | --- |
| finn-a-RHEUMA_NOS | IL-1Ra | Other/unspecified rheumatoid arthritis | 3 | -0.480164438 | 0.232555767 | 0.03894873 | 749 | 95244 | 2020 |
| finn-a-RHEUMA_OTHER_WIDE | IL-1Ra | Other (seronegative) rheumatoid arthritis, wide | 3 | -0.406223552 | 0.195923135 | 0.038136719 | 1063 | 95244 | 2020 |
